# A community detection algorithm using network topologies and rule-based hierarchical arc-merging strategies

**DOI:** 10.1371/journal.pone.0187603

**Published:** 2017-11-09

**Authors:** Yu-Hsiang Fu, Chung-Yuan Huang, Chuen-Tsai Sun

**Affiliations:** 1 Department of Computer Science, National Chiao Tung University, Hsinchu, Taiwan; 2 Department of Computer Science and Information Engineering, School of Electrical and Computer Engineering, College of Engineering, Chang Gung University, Taoyuan, Taiwan; Universidad Rey Juan Carlos, SPAIN

## Abstract

The authors use four criteria to examine a novel community detection algorithm: (a) *effectiveness* in terms of producing high values of normalized mutual information (NMI) and modularity, using well-known social networks for testing; (b) *examination*, meaning the ability to examine mitigating resolution limit problems using NMI values and synthetic networks; (c) *correctness*, meaning the ability to identify useful community structure results in terms of NMI values and Lancichinetti-Fortunato-Radicchi (LFR) benchmark networks; and (d) *scalability*, or the ability to produce comparable modularity values with fast execution times when working with large-scale real-world networks. In addition to describing a simple hierarchical arc-merging (HAM) algorithm that uses network topology information, we introduce rule-based arc-merging strategies for identifying community structures. Five well-studied social network datasets and eight sets of LFR benchmark networks were employed to validate the correctness of a ground-truth community, eight large-scale real-world complex networks were used to measure its efficiency, and two synthetic networks were used to determine its susceptibility to two resolution limit problems. Our experimental results indicate that the proposed HAM algorithm exhibited satisfactory performance efficiency, and that HAM-identified and ground-truth communities were comparable in terms of social and LFR benchmark networks, while mitigating resolution limit problems.

## Introduction

Many real-world systems can be expressed as networks consisting of nodes connected by edges [1–3]. In social networks, nodes represent individuals, and edges are used to mark connections such as friendships and family relations. In scientific collaboration networks, nodes and edges respectively represent scientists and collaborations among scientists for published academic papers. In web graphs, nodes and edges respectively correspond to URLs and hyperlinks. Primary properties exhibited by networks include the small-world effect (indicating a high degree of clustering and low degree of separation) [4], long tails (indicating a power-law degree distribution in which a small number of nodes have stronger connections compared to other network nodes) [5], fractality (indicating combined degree distribution and negative assortativity coefficient slopes) [6–7], and community structure (indicating tight connections between nodes with similar features within groups, and loose connections between nodes across multiple groups) [1–2, 8–13].

Network community detection, especially community structure, is currently receiving significant attention from researchers ranging from engineers and computer scientists to business and marketing specialists. The primary goal of community detection is identifying densely connected groups of network nodes and/or graph partitions that satisfy specific criteria such as edge connectivity compactness [1, 8–11]. The community detection problem is a well-studied NP-complete graph partition problem [14–15], and researchers in multiple disciplines have proposed various approaches to approximating community detection problem solutions. In computer science, a large number of solutions involve evolutionary computation [16–30] and artificial intelligence [31–33]. Complex network researchers initially used standard hierarchical clustering algorithms [2, 8–9], but eventually moved toward approaches based on modularity optimization [8, 32, 34–35], label propagation [36–41], data mining [42–44], and information theory [45–46]. Others have used density-based [47–48] and topology-based [49] algorithms.

Modularity [1, 10–11, 32] (a widely used measure for evaluating community structure quality when a network lacks a ground-truth community) involves evaluations of edge densities within and across communities, with higher modularity values indicating stronger community structures or better network partition quality. Thus, modularity can be used as a fitness function in evolutionary computation approaches, or as an objective optimization function for finding optimum community detection solutions. However, care must be taken to identify and respond to resolution limit problems [41, 50] that can arise when a community’s small size makes it a likely candidate for absorption by a larger community. Methods that use modularity for fitness or objective functions tend to experience resolution limit problems.

Normalized mutual information (NMI) [51] is a preferred approach for verifying the correctness of algorithm-identified community structures when a network has a ground-truth community partition for calculating similarities between actual and identified partitions. Since the Lancichinetti-Fortunato-Radicchi (LFR) benchmark model [52–53] generates networks with actual partitions, a combined NMI-LFR benchmark network approach can be used to examine an algorithm’s identification capabilities. Further, NMI can be used to test whether an algorithm mitigates resolution limit problems according to predefined synthetic networks (e.g., Clique-ring and Clique-pair networks) [41, 50]. However, most real-world networks lack partitions, and networks with ground-truth communities are rare.

Theoretical benchmarks and actual network partitions can be used in combination to determine the ability of an algorithm to produce correct community detection results. One algorithm may be better than another for LFR benchmark networks, but not for real networks. In other cases, an algorithm may successfully mitigate resolution limit problems in one kind of synthetic network, but not in another. We believe that community detection algorithms should satisfy four criteria: (a) *effectiveness* in terms of producing high NMI and modularity values, using well-known social networks for testing; (b) *examination*, meaning the ability to examine resolution limits using NMI values and synthetic networks; (c) *correctness*, meaning the ability to identify useful community structure results in terms of NMI values and LFR benchmark networks; and (d) *scalability*, or the ability to produce comparable modularity values with fast execution times when working with large-scale real-world networks.

In this paper we will apply rule-based strategies to community detection tasks, and offer alternative strategies for identifying network community structures. We will also describe our proposal for a simple hierarchical arc-merging (HAM) algorithm that includes a measure for computing the similarities (weights) of adjacent nodes connected by an edge, and for ranking edges based on these weights. There are at least five advantages to using a rule-based HAM algorithm: strategies are easy to implement because they primarily use if-else code statements; rule structures for tasks such as edge merging are explicit; rule-based programs are efficient because they only need to check rule-defined situations; the HAM algorithms have higher community detection resolution; and they can be extended to node-based methods.

To validate the proposed HAM algorithm according to the four criteria described above, we used five well-studied social network datasets to test community detection (effectiveness criterion), two synthetic networks to examine its susceptibility to resolution limit problems (examination criterion), eight sets of LFR benchmark networks to identify ground-truth community structures (correctness criterion), and eight large-scale real-world complex networks to measure performance (scalability criterion). Our experimental results indicate that the proposed HAM algorithm is capable of producing high NMI and modularity values for identified community structures, and that those structures are similar to ground-truth community structures in social and LFR benchmark networks, thereby reducing the potential for two kinds of resolution limit problems in synthetic networks. At the same time it produces comparable modularity values for identified community structures, and satisfactory performance for large-scale real-world complex networks.

## Background

To represent a network, let an undirected and weighted graph *G* = (*V*,*E*,*W*), where *V* is the node set, *E* the edge set, and *W* the edge weight. |*V*| denotes the number of nodes, |*E*| the number of edges, and |*W*| the sum of all edge weights. Network topology is represented as an adjacency matrix *A* = {*a*_*ij*_} and *a*_*ij*_ ∈ *R*^*n*^, where *a*_*ij*_ = 1 if an edge *e*_*ij*_ exists between nodes *i* and *j*, otherwise *a*_*ij*_ = 0. *w*_*ij*_ = *w*_*ji*_ denotes the weight of an edge *e*_*ij*_, where *w*_*ij*_ = 1 if nodes *i* and *j* in a network are identical and *a*_*ij*_ = 1, otherwise *w*_*ij*_ = 0.

### Similarity measures

To capture network topology information for weighted networks for community detection tasks, similarity measures are generally used to determine edge weights and network characteristics for the purpose of identifying dense structures [54]. The most common approach for determining weight *w*_*ij*_ of an edge *e*_*ij*_ is to calculate the number of common neighbors—that is, *w*_*ij*_ = *w*_*ji*_ = *S*_*cn*_(*i*,*j*), as in (1). A high weight indicates a high degree of similarity and structural equivalence (i.e., connected nodes sharing large numbers of common neighbors). *S*_*cn*_ can be extended to various similarity measures by dividing different denominator forms such as cosine similarity, the Jaccard index, and minimum similarity, respectively defined as

$$S_{cn}(i,j)=|\Gamma (i)\cap \Gamma (j)|$$ (1)

$$S_{cosine}(i,j)=\frac{\left|\Gamma (i)\cap \Gamma (j)\right|}{\sqrt{\left|\Gamma (i)||\Gamma (j)\right|}}$$ (2)

$$S_{Jaccard}(i,j)=\frac{\left|\Gamma (i)\cap \Gamma (j)\right|}{\left|\Gamma (i)\cup \Gamma (j)\right|}$$ (3)

$$S_{min}(i,j)=\frac{\left|\Gamma (i)\cap \Gamma (j)\right|}{min(\left|\Gamma (i)|,|\Gamma (j)\right|)}$$ (4)

where *Γ*(*i*) is the neighbor set of node *i*, *Γ*(*j*) the neighbor set of node *j*, |*Γ*(*i*)| the neighbor number of node *i*, |*Γ*(*j*)| the neighbor number of node *j*, and *min*(*x*,*y*) a minimum-value retrieval function.

### Community detection approaches

As mentioned above, researchers in many disciplines have proposed approaches for finding approximate solutions for community detection problems. Computer scientists have offered evolutionary computation approaches such as single-objective (e.g., Meme-Net, MIGA and TPEF) [16–17, 27, 29] and multiple-objective evolutionary algorithms (EAs) (e.g., GANet, MOGA-Net, MOEA/D-Net and APMOEA) [18–20, 23–26, 28], ant colony optimization (e.g., ACCFP) [21–22], and particle swarm optimization (e.g., MODPSO) [19, 30]. Proposed artificial intelligence approaches include greedy algorithms [32] and simulated annealing (SA) [31]. All of these methods have been used to address community detection problems.

In complex network research, Girvan–Newman (GN) [2] and Fast–Newman (FN) [32] algorithms were initially applied to common community detection problems using hierarchical clustering and greedy searches. This was followed by several modularity optimization approaches (e.g., CNM [8] and Louvain method [34]) to finding approximate solutions by merging pairs of nodes (or communities) according to the maximum Q of a modularity measure or modularity density [55]. Some researchers then proposed label propagation approaches (e.g., LPA [36], LPAm [37], LPAm+ [38], sub-community integration [39], CenLP [40], LPW [49] and Core-Nodes based LAP [56]) in which node labels are propagated throughout entire networks, with nodes assigned to communities based on the maximum number of neighboring labels, and with community structures identified until a steady level of label propagation is achieved.

Data mining approaches have been adapted to handle non-overlapping (e.g., *k*-medoids [42]) and overlapping (e.g., fuzzy *c*-means [43] and rough-fuzzy [44]) community detection problems in which certain nodes belong to multiple communities. Infomap [45], an information theory approach, uses random walk and Huffman coding methods to reveal a network’s community structures by minimizing its map equation—that is, its movement entropies between and within modules. Two density-based approaches, DenShrink [47] and ImDS [48], use similarity measures to calculate edge similarities, to extract topology characteristics from a network, and to identify community structures by merging or shrinking pairs of nodes according to degrees of similarity among edges.

### Community detection validation

Accurately measuring network partition quality is an important issue in light of the large number of potential partitions. Modularity and NMI measures depend on the presence or absence of a ground-truth community. When none exists, modularity [1, 10–11, 32] is often used as a fitness or objective function for evaluating community structure quality. A meaningful network partition contains many intra-community edges, but only a small number of inter-community edges. The term “meaningful” indicates that for an identified community and its randomized version, the number of intra-community connections should exceed the expected value of randomized intra-community connections, with both identified and randomized communities having the same degree sequences or numbers of nodes and edges. Thus, a randomized network is often used as a modularity null model. For any given network with *M* communities, modularity *Q* is defined as

$$Q=\sum_{i=1}^{M}\left(\varepsilon_{ii}-\alpha_{i}^{2}\right)=\sum_{i=1}^{M}\left(\frac{l_{i}}{\left|E\right|}-{\left(\frac{d_{i}}{2|E|}\right)}^{2}\right)$$ (5)

where *ε*_*ii*_ is the fraction of edges connected to endpoints in the same community *i*, *α*_*i*_ the fraction of edges connected to endpoints in community *i*, *l*_*i*_ the number of edges with two endpoints within community *i*, and *d*_*i*_ the summed degree of nodes in community *i*. A higher modularity value indicates better community structure quality. Unfortunately, resolution limits are a serious problem inherent to modularity [50]. In modularity optimization algorithms, small community size increases the potential of any community being absorbed into a larger community, thereby increasing the potential for overlooking important network substructures. Researchers who use modularity alone to identify communities should therefore consider ways of avoiding resolution limits.

For cases where ground-truth communities are present, the NMI [51] and LFR benchmark models [52–53] can be used to measure community structure quality associated with an algorithm—that is, they can be used to calculate levels of similarity between actual *A* partitions and identified *B* partitions. Here NMI is defined as

$$NMI(A,B)=\frac{-2\sum_{i=1}^{C_{A}}\sum_{j=1}^{C_{B}}\frac{N_{ij}N}{N_{i.}N_{.j}}}{\sum_{i=1}^{C_{A}}N_{i.}log\frac{N_{i.}}{N}+\sum_{j=1}^{C_{B}}N_{.j}log\frac{N_{.j}}{N}}$$ (6)

where *C*_*A*_ is the number of actual communities, *C*_*B*_ the number of identified communities, *N* a confusion matrix, *N*_*ij*_ the number of nodes shared in common between communities *C*_*A*_ and *C*_*B*_, *N*_*i*._ the sum over row *i* of matrix *N*, and *N*_*i*._ the sum over column *j* of matrix *N*. The NMI value range is between 0 and 1. If *NMI*(*X*,*Y*) = 1, the two partitions are considered identical, otherwise they are considered independent. A combination of NMI and two kinds of predefined synthetic networks (Clique rings and Clique pairs networks [51]) can be used to determine whether an algorithm suffers from a resolution limit problem.

In order to satisfy the four criteria, we developed a four-part process to determine the appropriateness of a community detection algorithm. For the effectiveness criterion, a mix of five social networks, one small-scale LFR benchmark network, and multiple modularity and NMI measures were used to analyze the quality of identified community structures. For the examination criterion, two kinds of synthetic networks and a NMI measure were used to determine the presence of a resolution limit problem. For the correctness criterion, LFR benchmark networks and a NMI measure were used to verify the quality of identified community structures compared to an actual partition. For the scalability criterion, eight large-scale real-world complex networks and a modularity measure were used to analyze community structure quality and performance efficiency (e.g., execution time analysis).

## Method

**Fig 1:**
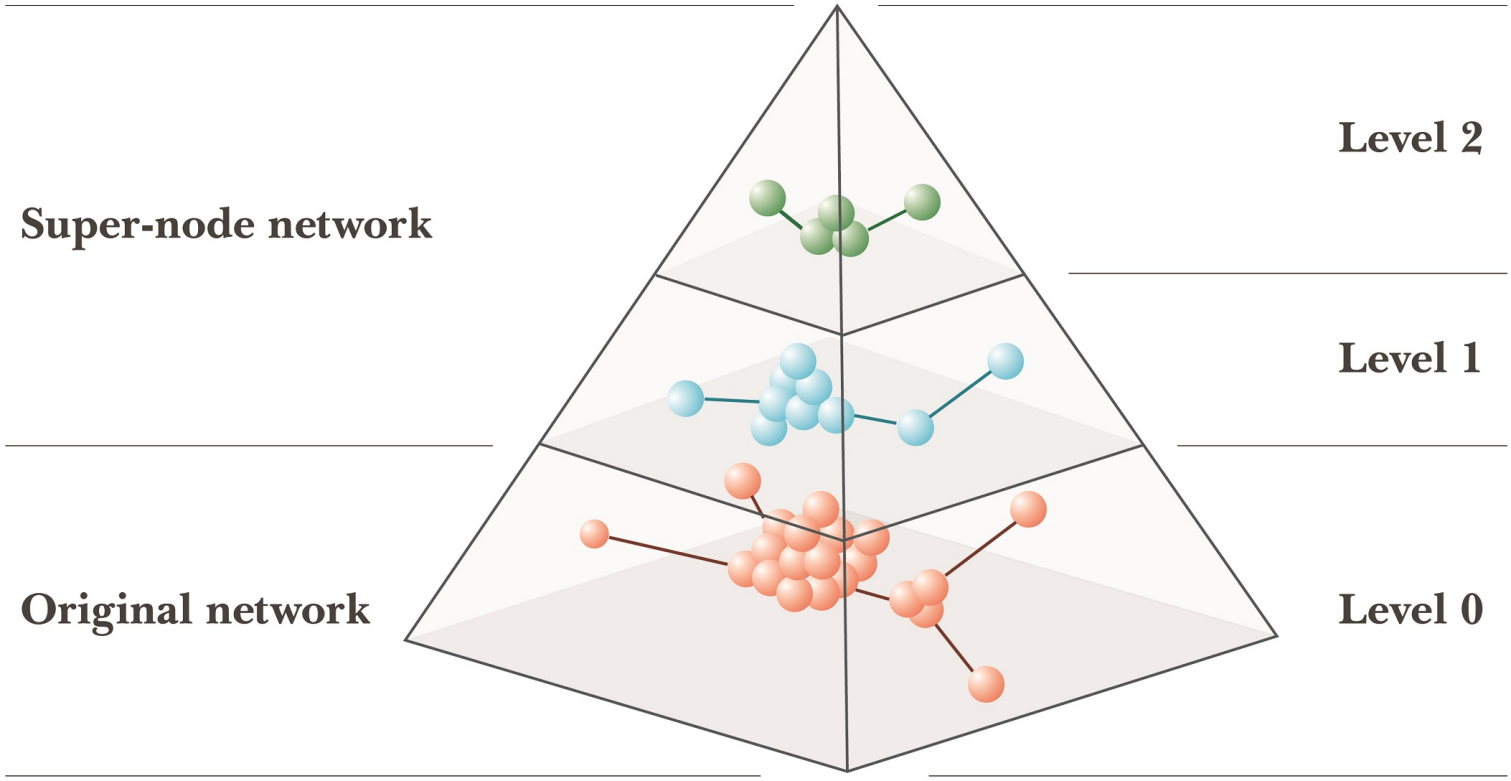
HAM community detection architecture.

Consisting of an original network phase and a super-node network phase (Fig 1), our proposed HAM algorithm uses network topologies and rule-based arc-merging strategies to identify community structures. In the original network phase, a similarity measure is used to calculate edge weights and to obtain network topology information, after which rule-based strategies are used to identify major communities and to preprocess a super-node network structure. During the super-node network phase, the combination of a proposed modularity optimization equation and rule-based strategies is applied to construct the entire super-node network structure. HAM stops and returns community detection results when network modularity can no longer be improved.

### Rule-based arc-merging strategies

The hierarchical arc-merging (edge- or node-merging) mechanism has been widely used for designing community detection algorithms [2, 8, 32, 34, 47–48]. It can be explicitly defined in terms of corresponding rule-based arc-merging strategies—that is, one strategy can be used to identify communities, and another to connect them. We established five arc-merging rules that can be combined to create different strategies. For each edge *e*_*ij*_ = (*v*_*i*_,*v*_*j*_) ∈ *E* and *v*_*i*_,*v*_*j*_ ∈ *V*, the arc-merging rules are defined as:

R1: Create a super-node *sn* that merges endpoints *v*_*i*_ and *v*_*j*_.
R2: If endpoint *v*_*i*_ is unmerged but endpoint *v*_*j*_ is merged with a super-node *s*_*j*_, then merge *v*_*i*_ with the super-node *sn*_*j*_ (or retain *v*_*i*_ as a super-node *sn*_*i*_).
R3: If endpoint *v*_*i*_ is merged with super-node *s*_*i*_ but endpoint *v*_*j*_ remains unmerged, then merge *v*_*j*_ with super-node *sn*_*i*_ (or retain *v*_*j*_ as a super-node *sn*_*j*_).
R4: Retain *v*_*i*_ and *v*_*j*_ as super-nodes *sn*_*i*_ and *sn*_*j*_.
R5: Otherwise, do not merge *v*_*i*_ and *v*_*j*_.

We used these rules to construct three kinds of strategies: community-creating (T1, which uses R1, R2, R3 and R5), structure maintenance (T2, which uses R2, R3, R4 and R5), and sink-shrinking (T3, which uses R2, R3 and R5). Details regarding the application of rule-based strategies for each HAM phase are presented as S1 File.

### Original network phase

Building on previous community detection studies [34–36, 38, 47], we believe that the characteristics of community structures can be captured by an explicit (deterministic) procedure. Hence, in the original network phase of HAM, we added a procedure consisting of calculating edge weights, classifying edges according to their weights, and merging edge endpoints according to a rule-based strategy for community detection. After calculating the edge weights or similarities of two endpoints and identifying the dense or loose parts of network components, component edges are classified as weighted-edge *E*^*W*^, bridge *E*^*B*^, or sink *E*^*S*^. These three edge classes are defined as:

$$E^{W}=\{e_{ij}|w_{ij}>0\}$$ (7)

$$E^{B}=\{e_{ij}|w_{ij}=0,k_{i}>1\;and\;k_{j}>1\}$$ (8)

$$E^{S}=\{e_{ij}|w_{ij}=0,k_{i}=1\;or\;k_{j}=1\}$$ (9)

where *k*_*i*_ is the degree of node *i* and *k*_*j*_ the degree of node *j*. *V* = *V*(*E*^*W*^) ∪ *V*(*E*^*B*^) ∪ *V*(*E*^*S*^) and *E* = *E*^*W*^ ∪ *E*^*B*^ ∪ *E*^*S*^, where *V*(*E*^*W*^) is the weighted-edge node set, *V*(*E*^*B*^) the bridge-edge node set, and *V*(*E*^*S*^) the sink-edge node set.

**Fig 2:**
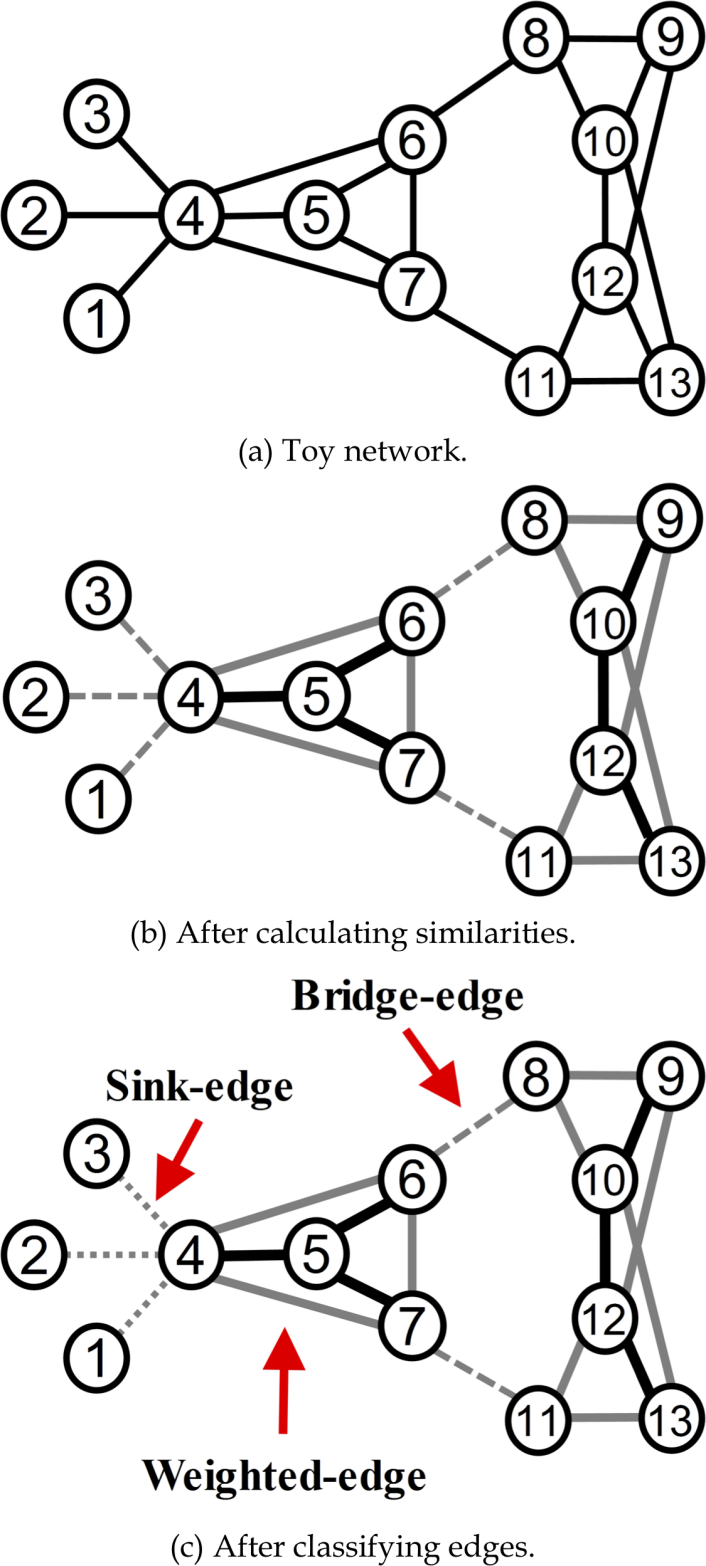
Edge classification results for a toy network. (a) The network, (b) after calculating similarities, (c) after classifying edges.

After classifying edge weights (Fig 2), *E*^*W*^ weighted edges are said to have greater similarity and higher summed node degrees, indicating that they are within the denser parts of communities—see, for example, edges (4, 5), (9, 10) and (12, 13) in Fig 2. Further, *E*^*B*^ bridge edges have higher degrees of either or both endpoints, indicating that they connect different communities—see edges (6, 8) and (7, 11) in the figure. Otherwise, the edges might be one part of a long bridge consisting of multiple edges. *E*^*S*^ sink-edges such as (1, 4), (2, 4) and (3, 4) have only single community connections.

Next, weighted-edge and bridge-edge classes are sorted and arranged in decreasing order according to two indexes: edge weight *w*_*ij*_ and the *k*_*i*_ + *k*_*j*_ summed degree of edge endpoints. An edge with high weight *w*_*ij*_ is perceived as having shared endpoints with a large number of neighbors, perhaps serving as the center of a community, group, or clique. However, the size of this community is unknown—it could be large or small. Accordingly, edge sorting involving *w*_*ij*_ weights and *k*_*i*_ + *k*_*j*_ summed degrees in decreasing order represents edge priorities in a small community. High-priority edges in such sequences are considered candidates for community foci.

To give an example, assume that edges *e*_*x*_ and *e*_*y*_ have identical weights as determined by minimum similarities (*w*_*x*_ = 3/*min*(4,5) = 0.75 and *w*_*y*_ = 15/*min*(20,25) = 0.75), but with different *k*_*i*_ + *k*_*j*_ values (9 and 45, respectively). Although the two edges have identical proportions of common neighbors, in terms of neighbor endpoint connections edge *e*_*y*_ likely captures more community information, and is therefore preferred for merging purposes early in the arc-merging process. Hence, an edge with a higher *k*_*i*_ + *k*_*j*_ value should be promoted and its priority increased in any sequence that is sorted during the arc-merging process in the original network phase. For the bridge-edge class, any edge with a high *k*_*i*_ + *k*_*j*_ should be considered an important bridge for connecting two communities, and therefore be given a higher priority during the arc-merging process.

According to this procedure, community structures are constructed from densest-to-loosest according to the order of sorted edges plus three rule-based strategies:

1. For *E*^*W*^ edges, the T1 community-creating strategy is used to merge the endpoints of edges into super-nodes for use as seeds (R1), to attract unmerged nodes located close to these seeds (R2 and R3), and to handle all other cases tied to creating edges for constructing network structure (R5). After T1 is completed, a preprocessed high-level network structure consisting of super-nodes is created.
2. For *E*^*B*^ edges, the T2 structure maintenance strategy is used to create edges for connecting isolated communities (R4), to attract nearby nodes with one edge endpoint that is already inside a community (R2 and R3), and to handle all other cases (R5). All isolated communities are connected after applying T2.
3. For *E*^*S*^ edges, the T3 sink-shrinking strategy is used to handle edges with edge endpoints (either one) of 1 degree (i.e., *d*_*i*_ = 1 or *d*_*j*_ = 1), and to address all other cases (R5). Although the functionality of T3 is part of strategy T2, we will consider T3 as independent for purposes of describing the HAM rule-based strategy.

### Super-node network phase

The procedure for the super-node network phase is similar to that for the original network phase. After edge similarity is measured in terms of the summed weights of all edges between two super nodes, modularity optimization is applied to determine whether any edge endpoint pairs should be merged into a high-level super-node based on a calculation of the Δ*Q* value of edges contributing to network modularity. The summed weight and Δ*Q* equations are expressed as

$${\hat{w}}_{ij}=\sum_{\begin{array}{c} v_{i}\in m_{i},v_{j}\in m_{j},\\ v_{i}\neq v_{j}\in V \end{array}}^{}w_{ij}$$ (10)

$$\begin{array}{l} {\Delta Q}_{ij}=Q_{m_{ij}}-Q_{m_{i}}-Q_{m_{j}}\\ =\left[\frac{l_{ij}}{\left|E\right|}-{\left(\frac{d_{ij}}{2|E|}\right)}^{2}\right]-\left[\frac{l_{i}}{\left|E\right|}-{\left(\frac{d_{i}}{2|E|}\right)}^{2}\right]-\left[\frac{l_{j}}{\left|E\right|}-{\left(\frac{d_{j}}{2|E|}\right)}^{2}\right]\\ =\frac{\left(l_{ij}-l_{i}-l_{j}\right)}{\left|E\right|}-\frac{\left(d_{ij}^{2}-d_{i}^{2}-d_{j}^{2}\right)}{4{\left|E\right|}^{2}}\\ =\frac{1}{\left|E\right|}\left[(l_{ij}-l_{i}-l_{j})-\frac{1}{4|E|}(d_{ij}^{2}-d_{i}^{2}-d_{j}^{2})\right] \end{array}$$ (11)

where *m*_*i*_ is community (or super-node) *i*, ${\hat{w}}_{ij}$ the summed weights of edges between communities *m*_*i*_ and *m*_*j*_, Δ*Q*_*ij*_ the incremental value of modularity as contributed by edge *e*_*ij*_, $Q_{m_{ij}}$ the partial modularity value after merging communities *m*_*i*_ and *m*_*j*_, $Q_{m_{i}}$ the partial modularity value before merging community *m*_*i*_, *l*_*ij*_ the number of edges in merged community *m*_*ij*_, *l*_*i*_ the number of edges in community *m*_*i*_, *d*_*ij*_ the summed degree of nodes in merged community *m*_*ij*_, and *d*_*i*_ the summed degree of nodes in community *m*_*i*_. If *l*_*ij*_ = *l*_*i*_ + *l*_*j*_ + |*e*_*ij*_|, then (11) can be simplified as

$${\Delta Q}_{ij}=\frac{1}{\left|E\right|}\left[e_{ij}-\frac{1}{4|E|}(d_{ij}^{2}-d_{i}^{2}-d_{j}^{2})\right]$$ (12)

where (according to formulas 11 and 12) network topology information (i.e., *l*_*ij*_, *l*_*i*_, *l*_*j*_, *d*_*ij*_, *d*_*i*_, *d*_*j*_ and |*e*_*ij*_|) is used for delta-Q calculations. This information, which is updated during the arc-merging process, can be applied immediately. Weighted network information only uses edge weights *w*_*ij*_ in the original network phase and summed weights ${\hat{w}}_{ij}$ in the super-node network phase for sorting edges in decreasing order.

After calculating their summed weights and Δ*Q* values, edges are classified as deltaQ-edge *E*^Δ*Q*^ or bypass-edge *E*^*P*^. *E*^Δ*Q*^ denotes a set of edges with Δ*Q*_*ij*_ values greater than zero—in other words, the merging of two edge endpoints carries the potential to increase the Δ*Q* of the entire network and improve community structure quality. *E*^*P*^ consists of a set of unmerged edges. The two classes are defined as

$$E^{\Delta Q}=\{e_{ij}|{\hat{w}}_{ij}>0\;and\;{\Delta Q}_{ij}>0\}$$ (13)

$$E^{P}=\left\{e_{ij}|({\hat{w}}_{ij}\geq 0\;and\;{\Delta Q}_{ij}\leq 0)\;or\;({\hat{w}}_{ij}=0\;and\;{\Delta Q}_{ij}\geq 0)\right\}$$ (14)

Following edge classification in the super-node network phase, *E*^Δ*Q*^ edges have higher weights and Δ*Q* values, indicating their positions between two dense components and their ability to increase the incremental Δ*Q* value of the entire network. *E*^*P*^ edges are only used to maintain the super-node network structure. Next, *E*^Δ*Q*^-class edges are sorted and arranged in decreasing order according to two indexes: the Δ*Q*_*ij*_ value of edges and the ${\hat{w}}_{ij}$ summed weights of edges. After sorting, the T1 and T2 rule-based strategies are used to create and maintain a high-level super-node network structure. For *E*^Δ*Q*^ edges, T1 is used to create super-nodes as seeds for attracting unmerged nodes that are close to the super-node, as well as to handle all other cases. After executing T1, a preprocessed high-level network structure is created. For *E*^*B*^ edges, T2 is used to create edges for connecting various communities, to attract nodes that are close to communities, and to handle all other cases. After executing T2, a high-level super-node network is completed.

### The proposed algorithm

**Fig 3:**
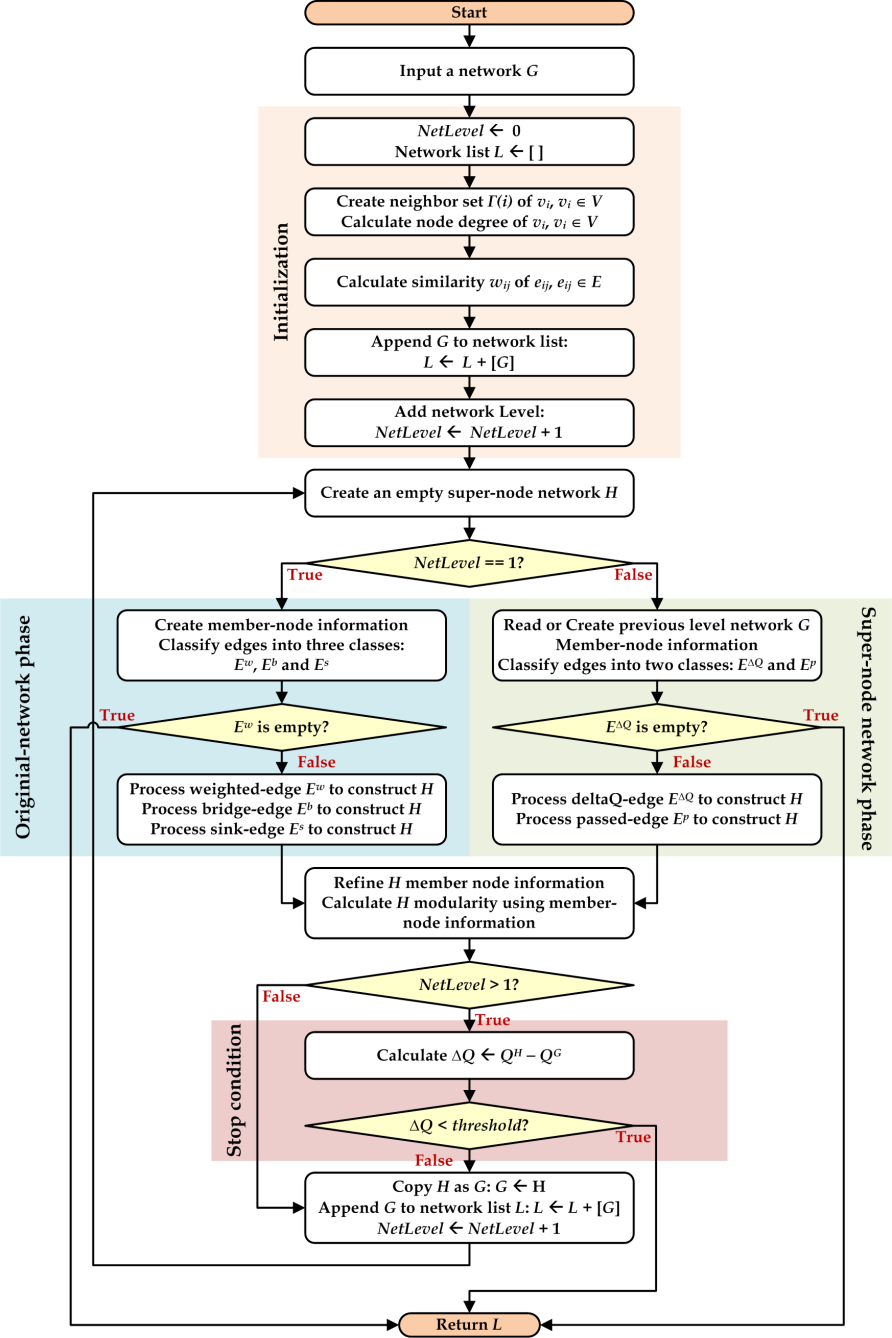
HAM algorithm flowchart.

A HAM flowchart is presented as Fig 3 and details presented as Algorithm 1. For any given network *G*, a set of neighbors for each node in the original network is created, a similarity measure is used to calculate edge weights, and the original network is appended to the network list. During the original network phase, an empty network *H* is created as a super-node network for further construction. Next, edges are classified as *E*^*W*^, *E*^*B*^ or *E*^*S*^. Three rule-based strategies (Algorithms A1-3 in S1 File) are applied during the original network phase: a strategy for creating communities, a maintenance strategy for connecting communities, and a sink-shrinking strategy for handling the edge endpoints with node degree *k*_*i*_ = 1. After applying these strategies, all member-node information for the super-node network is refined. The constructed super-node network is preserved and appended to the network list. As part of the super-node network phase, an empty super-node network is created, Δ*Q*_*ij*_ edge values are calculated, and edges are classified as *E*^Δ*Q*^ or *E*^*P*^. Two rule-based strategies (Algorithms A4-5 in S1 File) are used to merge super-nodes and to construct a high-level super-node network structure, after which member-node information is refined and used to calculate network modularity values. HAM continues this arc-merging procedure until the Δ*Q* increment of the entire network is below a threshold, or until there are no more Δ*Q* edges. Last, community structures are identified. See S2 File for a step-by-step example.

### Algorithm 1. Hierarchical arc-merging (HAM) algorithm

#### Time complexity

Time complexity analyses begin with node and edge pre-processing (steps 1 to 9) according to *O*(⟨*k*⟩ ∙ *V*) and *O*(*E*). The subsequent original network phase (steps 11 to 20) entails (a) the creation of member-node information using *O*(*V*), and (b) edge classification and sorting. Briefly, edge classification entails *O*(*E* + E^*^logE^*^), where E^*^ depends on *E*^*W*^ or *E*^*B*^, and where three rule-based strategies utilize *O*(*c* ∙ *E*), with *c* denoting the cost of merging nodes. The third part of time complexity analysis is a super-node network phase (steps 22 to 28), in which member-node information uses *O*(*V*′), with *V*′ denoting the nodes of super-node network *G* (step 35). Edge classification uses *O*(*E*′ + *E*^Δ*Q*^*logE*^Δ*Q*^), with *E*′ denoting the edges of super-node network *G*, and *E*^Δ*Q*^ denoting Δ*Q* edges. Both rule-based strategies use *O*(*c* ∙ *E*′). The final time complexity analysis step uses *O*(*V*″) to refine member-node information, and *O*(*M*) to calculate the modularity value of super-node network *H*. For any given network, the HAM while loop runs *L*′ times (step 10) at a cost of *O*(*L*′). The time complexity of the original network phase dominates the *O*(E^*^logE^*^) HAM community detection process, hence the overall time complexity of HAM does not exceed *O*(*L*′ ∙ E^*^logE^*^). For extreme sorted edge cases, the overall time complexity of HAM does not exceed *O*(*L*′ ∙ *E*), meaning that the edge-sorting step is ignored. A step-by-step example of HAM time complexity estimation is shown as S3 File.

## Experimental results

We used two well-studied methods to establish HAM identification accuracy and performance efficiency baselines that fit with the four criteria: the Louvain method, which has a reputation for dealing successfully with a network consisting of 1 billion edges using a PC machine [34], and the Infomap information theory-based method, based on its history of producing optimum NMI results for LFR benchmark networks [45]. We designed experiments to compare HAM with the CNM [8] and Louvain modularity optimization methods, Infomap, and DenShrink (DS, a density-based method) [47]. All four are frequently used in community detection comparison experiments. They were implemented using the C (CNM) or C++ (Louvain, Infomap and DS) programming languages. Our proposed method was created with Python to take advantage of its code readability and package support characteristics, while accepting the disadvantage of slower execution times compared to C and C++. Our HAM python program is downloadable at https://github.com/yuhsiangfu/Hierarchical-Arc-Merging.

### Datasets

For the effectiveness criterion we used five well-studied social networks and one small-scale LFR benchmark network with ground-truth communities to verify community detection results in terms of matches between identified and actual communities. For the examination criterion, two synthetic networks were used to identify algorithm-associated resolution limit problems, if any. For the correctness criterion, we used the LFR model [52–53] to generate synthesized networks with different community structure properties, as well as to test the accuracy of algorithm-identified community structures. For the scalability criterion, eight large real-world networks were used to test performance efficiency. The giant connected component (GCC) of the social, synthetic, small-scale benchmark, and large-scale real-world networks used in our experiments is shown in Table 1.

**Table 1:** Giant connected component (GCC) of network statistics sorted by number of edges.

Dataset	|*V*|	|*E*|	⟨*C*_*c*_⟩	⟨*k*⟩	*k*_*max*_	*r*
Karate	34	78	0.5706	4.5882	17	-0.4756
Dolphins	62	159	0.2590	5.1290	12	-0.0436
Clique-ring-m = 5_n = 30	150	330	0.8800	4.4000	6	0.0833
Clique-pair-m = 20_p = 5	50	404	0.9600	16.1600	20	0.9410
Polbooks	105	441	0.4875	8.4000	25	-0.1279
Football	115	613	0.4032	10.6609	12	0.1624
LFR-benchmark-n = 300-u = 0.05	300	2209	0.5538	14.7267	49	-0.1262
Polblogs	1222	16714	0.3203	27.3552	351	-0.2213
Email-contacts	12625	20362	0.1088	3.2257	576	-0.3867
Brightkite	56739	212945	0.1734	7.5061	1134	0.0096
Com-youtube	51204	317393	0.1750	12.3972	1928	-0.0586
Com-amazon	315819	870161	0.3990	5.5105	548	-0.0572
Com-DBLP	260691	949360	0.6457	7.2834	343	0.2599
Loc-gowalla	196591	950327	0.2367	9.6681	14730	-0.0293
Web-google	855802	4291352	0.5190	10.0288	6332	-0.0555
Wiki-talk	2388953	4656682	0.0527	3.8985	100029	*

* Memory error in Numpy.

⟨*C*_*c*_⟩, average clustering coefficient; ⟨*k*⟩, average node degree; *k*_*max*_, maximal node degree; *r*, assortativity.

The five well-studied social networks used in this project are also listed in Table 1. The Zachary Karate Club network consists of 34 nodes (club members) and 78 edges (cross-member friendships) [57]. A split occurred due to a disagreement between the club’s administrator and instructor; the instructor left, taking one-half of the original members and creating a new club. The Dolphins network consists of 62 bottlenose dolphins living in Doubtful Sound, New Zealand. Based on observations between 1994 and 2001, 159 interactions between dolphin pairs took place, more than would be predicted by chance [58]. This network can be divided into two groups based on the departure of one key individual. The U.S. college football network consists of 115 teams and 613 games played during the 2000 season [2], with nodes representing teams and edges games played between teams. The teams are divided into 12 conferences, and play more games against conference than non-conference opponents. The political book network [59] consists of the purchase histories of customers who bought books on political topics from the Amazon.com website. Nodes indicate books (105) and edges co-purchasing relationships in which users bought more than one book (441). Purchased books were classified as conservative, neutral or liberal. The political blogs network [60] consists of 1,222 blogs about the 2004 American presidential election and 16,714 links among them. The blogs were manually divided into conservative and liberal categories.

The two synthetic networks shown in Table 1 were used to determine the presence of resolution limit problems [51]. The Clique-ring synthetic network consists of a ring of *w* cliques (with *w* an even number) connected by a single edge. Each clique is a complete *K*_*p*_ graph consisting of *p* nodes and [*p*(*p* − 1)]/2 edges. The Clique-pair synthetic network consists of two *K*_*p*_ (part one) and two *K*_*q*_ complete graphs (part two), both connected by single edges. Each part one clique is connected to two part two cliques. Clique-ring parameters are *p* = 5 and *r* = 30 (150 nodes and 330 edges). Clique-pair parameters are *p* = 20 and *q* = 5 (50 nodes and 404 edges).

One assumption of the LFR model is that node degree and community size follow a power-law distribution with the following parameters: *γ*, degree distribution exponent; *β*, community size distribution exponent; *k*_*max*_ and *k*_*min*_, upper and lower node degree boundaries, respectively; *z*_*max*_ and *z*_*min*_, community size constraints; mixing parameter *u*, the proportion of nodes sharing links with the nodes of other communities; and 1 − *u*, the proportion of nodes sharing links with other nodes in the same community. The LFR parameters used in this study are shown in Table 2 [16, 47, 53]. The mixing parameter u range was between 0.1 and 0.8 (0.05 increments). The LFR model generated 30 synthesized networks for each *u*. The small-scale LFR benchmark network described above was generated with 300 nodes and *u* = 0.05 (Table 1).

**Table 2:** LFR benchmark network parameters.

Dataset	|*V*|	|*E*|	*γ*	*β*	⟨*k*⟩	*k*_*max*_	*z*_*min*_	*z*_*max*_	*μ*
1000 (S)	1000	20000	2	1	20	50	10	50	[0.1, 0.8]
1000 (B)	1000	20000	2	1	20	50	20	100	[0.1, 0.8]
5000 (S)	5000	100000	2	1	20	50	10	50	[0.1, 0.8]
5000 (B)	5000	100000	2	1	20	50	20	100	[0.1, 0.8]
10000 (S)	10000	200000	2	1	20	50	20	50	[0.1, 0.8]
10000 (B)	10000	400000	2	1	40	100	50	100	[0.1, 0.8]
50000 (S)	50000	2000000	2	1	40	100	50	100	[0.1, 0.8]
50000 (B)	50000	2000000	2	1	40	200	100	200	[0.1, 0.8]

(S), small community size.

(B), big community size.

The eight large-scale real-world networks [61] can be further divided into the three large (|*V*| = 1000∼10000) and five very large (|*V*| ≥ 10000) real network groups shown in Table 1. The three large networks were (a) Email-contact, consisting of messages sent and received between email accounts at the Computer Sciences Department of London’s Global University; (b) Brightkite, representing users and friendships within a location-based social networking service; and (c) Com-youtube, representing users of and friendships made via the YouTube video-sharing website. The five very large networks were (a) the Com-amazon network, consisting of customer co-purchasing behaviors on the Amazon.com website; (b) the Com-DBLP network, representing authors and co-author publications found in a computer science bibliography database; (c) the Loc-gowalla network, consisting of users and friendships within a location-based social networking website; (d) the Web-google network, consisting of web pages and hyperlinks between web pages; and (e) Wiki-talk, representing Wikipedia users and their co-editor communications.

### Results

Experimental results for the effectiveness and examination criteria are shown in Table 3. The modularity, NMI, and execution time data for each method represent averages for 30 runs. The first part of the table contains results for five well-studied social networks and one small-scale LFR benchmark network. Modularity and NMI measures were used to verify the correctness of community detection results produced by the various methods.

**Table 3:** Results for social, synthetic and LFR networks.

Network	Karate				Dolphins				Football				Polbooks			
Measure	NMI	Q	T-avg	T-std	NMI	Q	T-avg	T-std	NMI	Q	T-avg	T-std	NMI	Q	T-avg	T-std
Louvain	0.6176	**0.4188**	***0*.*0291***	0.0053	0.5636	0.5185	***0*.*0307***	0.0028	0.8909	**0.6046**	***0*.*0307***	0.0028	0.5122	***0*.*5266***	***0*.*0307***	0.0028
CNM	0.7069	0.3807	0.0421	0.0071	0.6020	0.4955	0.0562	0.0086	0.7698	0.5773	0.0728	0.0093	0.5314	0.5020	0.0671	0.0071
DS	0.6301	***0*.*4156***	0.0515	0.0071	0.5766	0.4889	0.0504	0.0066	***0*.*9096***	***0*.*6032***	0.0629	0.0049	0.5145	0.5042	0.0598	0.0071
INFOMAP	***0*.*7112***	0.4020	0.0395	0.0078	**0.6441**	**0.5285**	0.0432	0.0066	**0.9242**	0.6005	0.0582	0.0069	***0*.*5413***	**0.5268**	0.0530	0.0076
HAM_Min_	**1.0000**	0.3715	**0.0005**	0.0028	***0*.*6132***	***0*.*5228***	**0.0016**	0.0047	0.9018	0.5551	**0.0062**	0.0076	**0.5646**	0.5199	**0.0026**	0.0058
Network	Polblogs				LFR-benchmark-n = 300-u = 0.05				Clique-ring-m = 5-n = 30				Clique-pair-m = 20-p = 5			
Measure	NMI	Q	T-avg	T-std	NMI	Q	T-avg	T-std	NMI	Q	T-avg	T-std	NMI	Q	T-avg	T-std
Louvain	0.6505	**0.4269**	**0.0473**	0.0028	**1.0000**	**0.8062**	***0*.*0322***	0.0039	0.8923	**0.8879**	***0*.*0317***	0.0049	***0*.*9401***	**0.5426**	***0*.*0307***	0.0028
CNM	***0*.*6609***	**0.4269**	0.7842	0.0867	**1.0000**	**0.8062**	0.1404	0.0201	***0*.*9074***	***0*.*8863***	0.0816	0.0112	***0*.*9401***	**0.5426**	0.0510	0.0080
DS	0.3173	0.0649	0.9230	0.0156	**1.0000**	**0.8062**	0.1284	0.0077	**1.0000**	0.8758	0.0577	0.0071	**1.0000**	***0*.*5416***	0.0608	0.0074
INFOMAP	0.5431	0.4245	0.3895	0.0148	**1.0000**	**0.8062**	0.0790	0.0056	0.7258	0.8152	0.0588	0.0077	**1.0000**	***0*.*5416***	0.0390	0.0078
HAM_Min_	**0.7125**	***0*.*4252***	***0*.*1352***	0.0109	**1.0000**	**0.8062**	**0.0146**	0.0056	**1.0000**	0.8758	**0.0016**	0.0047	**1.0000**	***0*.*5416***	**0.0021**	0.0053

**Bold**, best result.

**Bold** and *italics*, second-best result.

Subscript, similarity measure used in HAM.

For the ground-truth community NMI results, the HAM algorithm produced the highest NMI values according to the minimum similarity measure among the Karate, Polbooks, Polblogs, and small-scale LFR benchmark networks—that is, the identified community structures were the closest (or identical) to those of the networks’ ground-truth communities. Infomap had the highest NMI values for the Dolphins, College Football, and small-scale LFR benchmark networks. For modularity results, when ground-truth communities were removed, the Louvain method had the highest modularity values for the Karate, Football, Polblogs and small-scale LFR benchmark networks. Infomap had the highest modularity values for the Dolphins and Polbooks networks. At best, HAM performance for modularity can only be considered satisfactory. For execution time results, HAM unexpectedly had the fastest performance efficiency for all six small-scale networks, including the Louvain method. Further, we found that different methods produced the highest NMI or modularity values, but whenever a method concurrently produced the highest values for both NMI and modularity (e.g., the Dolphin and small-scale LFR benchmark networks using Infomap), actual community sizes were approximately equal.

Results for two kinds of synthetic networks are presented in the second part of Table 3, with modularity and NMI measures used to determine the presence, if any, of resolution limit problems. HAM and DS had the highest NMI values (equal to 1) for the two networks, indicating that the resolution limit problems had been mitigated to a certain degree, and that community structures were correctly identified. In comparison, the Louvain method produced the highest modularity results for the two synthetic networks, indicating the presence of resolution limit problems. The unstable results produced by Infomap indicate uncertainty regarding their presence.

**Fig 4:**
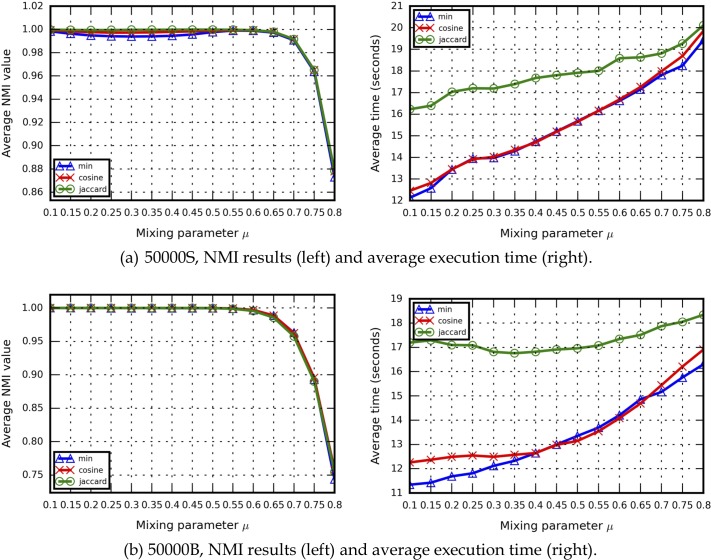
A comparison of similarities among the LFR benchmark networks used in this study. (a) 50000S, (b) 50000B.

For the correctness criterion, the LFR model was used to generate 30 benchmark networks for each *u* (450 networks total). The results shown in Fig 4 represent averages for all 30. The NMI measure was used to verify the correctness of identified community detection results. Regarding HAM similarity settings, we used 50000S and 50000B LFR benchmark networks to determine which similarity measures should be applied in our experiments, and found that all three resulted in similar NMI values, but with markedly different execution times. We observed that the minimum similarity measure resulted in the fastest execution times, that the cosine similarity measure performed as fast as the minimum similarity measure, and that the Jaccard index was the slowest. Based on the NMI and execution time results, we decided to use the cosine similarity measure in our experiments. Similarity comparison data for other LFR benchmark networks are presented in S4 File.

**Fig 5:**
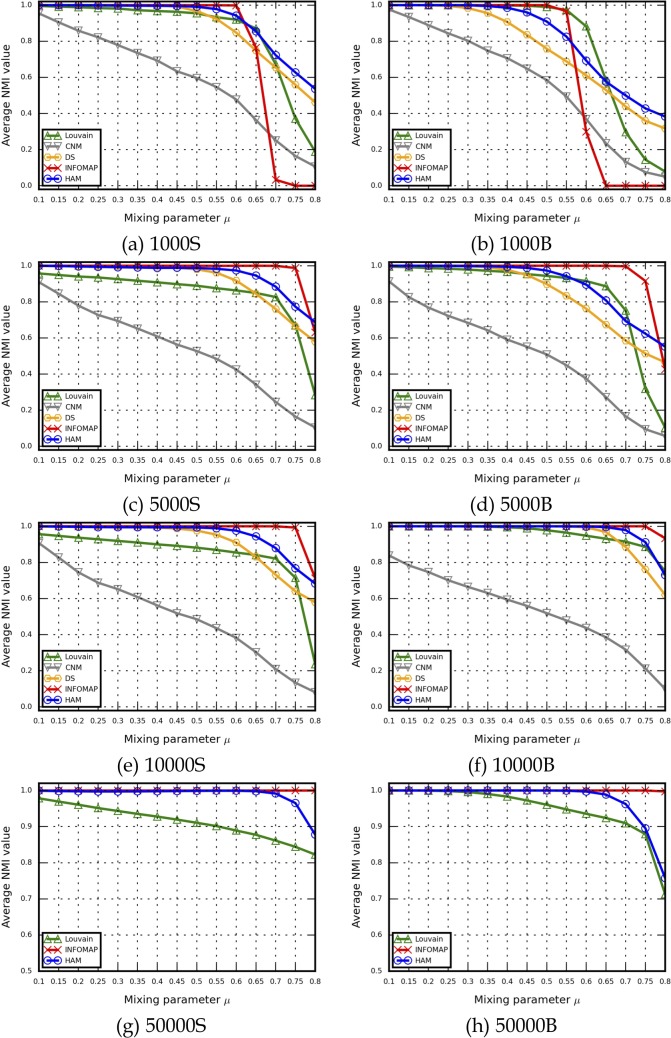
NMI results for the LFR benchmark networks used in this study. (a) 1000S, (b) 1000B, (c) 5000S, (d) 5000B, (e) 10000S, (f) 10000B, (g) 50000S, (h) 50000B.

**Fig 6:**
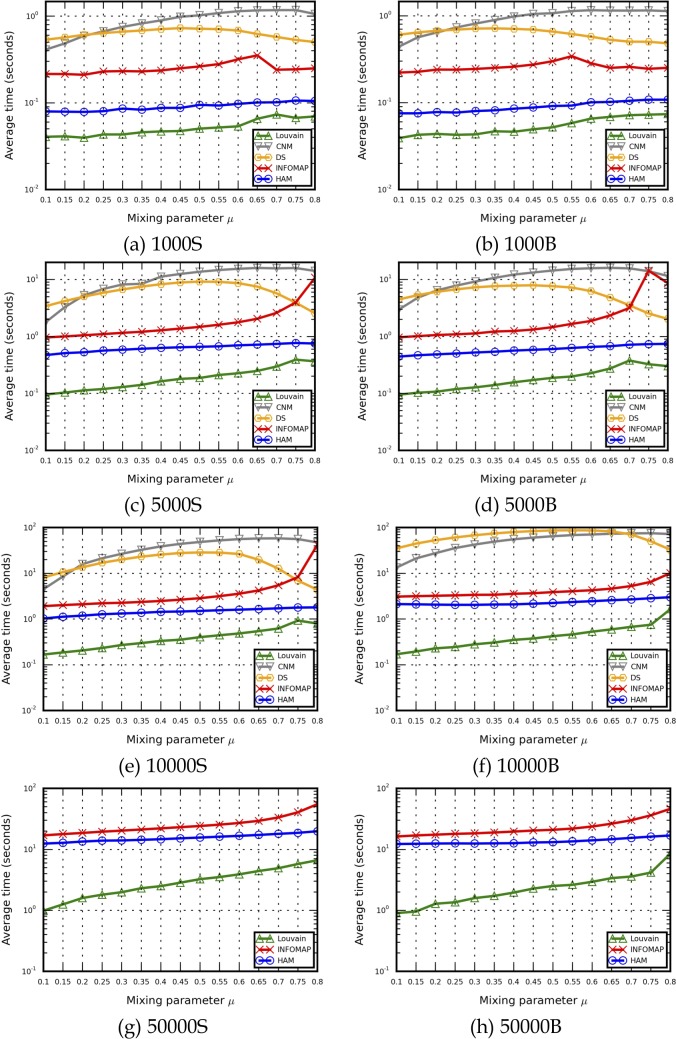
Execution time results for the LFR benchmark networks used in this study. (a) 1000S, (b) 1000B, (c) 5000S, (d) 5000B, (e) 10000S, (f) 10000B, (g) 50000S, (h) 50000B.

NMI and execution time results for LFR benchmark networks are shown in Figs 5 and 6, and detailed NMI and execution time data are presented in S5 and S6 Files. One CNM run required more than one hour for each u value for all 30 networks. Accordingly, generating results for all LFR benchmark network sets would require many hours or days of computing time. Due to a memory allocation error (“std::bad_alloc”) during DS execution, CNM and DS results are not shown in Figs 5G, 5H, 6G or 6H. As shown, for each method the overall NMI value decreased when *u* increased, meaning that the community structures became less distinct as the number of in-between edges increased, making community structures more difficult for algorithms to identify. NMI results from various methods were much closer to each other when *u* ≤ 0.5. NMI values produced by Infomap dropped sharply when *u* ≥ 0.6 (e.g., 1000S) or *u* ≥ 0.55 (e.g., 1000B), indicating that the network structure information may have been insufficient for random walkers to capture indistinct community structures. Similar decreases have been reported by other researchers [18–19, 28, 39, 53]. In contrast, when network structure information was sufficient (e.g., 5000S/B to 50000S/B), Infomap performed well in cases with *u* = 0.1∼0.8 ranges of distinct/indistinct community structures. In those cases, Infomap NMI results were best for the LFR benchmark networks (6 of 8 sets) (Tables I-M in S5 File).

Regarding cosine similarity, our NMI results indicate that HAM successfully identified community structures that were close (e.g., *u* ≤ 0.6 in 1000S/B or *u* ≤ 0.5 in 5000S/B) or identical to actual structures (e.g., *u* ≤ 0.7 in 10000S/B to 50000S/B). Compared to those produced by the Louvain method, HAM results were close (e.g., *u* = 0.6∼0.7 for 1000S and 5000B) or better (e.g., *u* ≥ 0.1 for 5000S and 10000S/B to 50000S/B). HAM results were significantly better than those produced by CNM and DS in terms of ground-truth community correctness. Combined, the data indicate that HAM produced the second best NMI results for the LFR benchmark networks (6 of 8 sets) (Tables I-M in S5 File).

According to the execution time results shown in Fig 6, the Louvain method had the best performance efficiency among the LFR benchmark networks. Despite being constructed with an interpreted programming language, HAM still outperformed CNM, DS and Infomap. Infomap’s performance efficiency results were satisfactory, with best NMI values produced when *u* ≤ 0.6. However, execution times increased sharply when *u* ≥ 0.6, meaning that random walkers required more time to find appropriate community structure boundaries. CNM execution time results indicate that the computing time required to identify the shortest paths between all node pairs increased as *u* increased. The high peaks in the DS execution time results may be due to an excessive number of choices for finding and merging micro-communities when *u* = 0.5.

**Fig 7:**
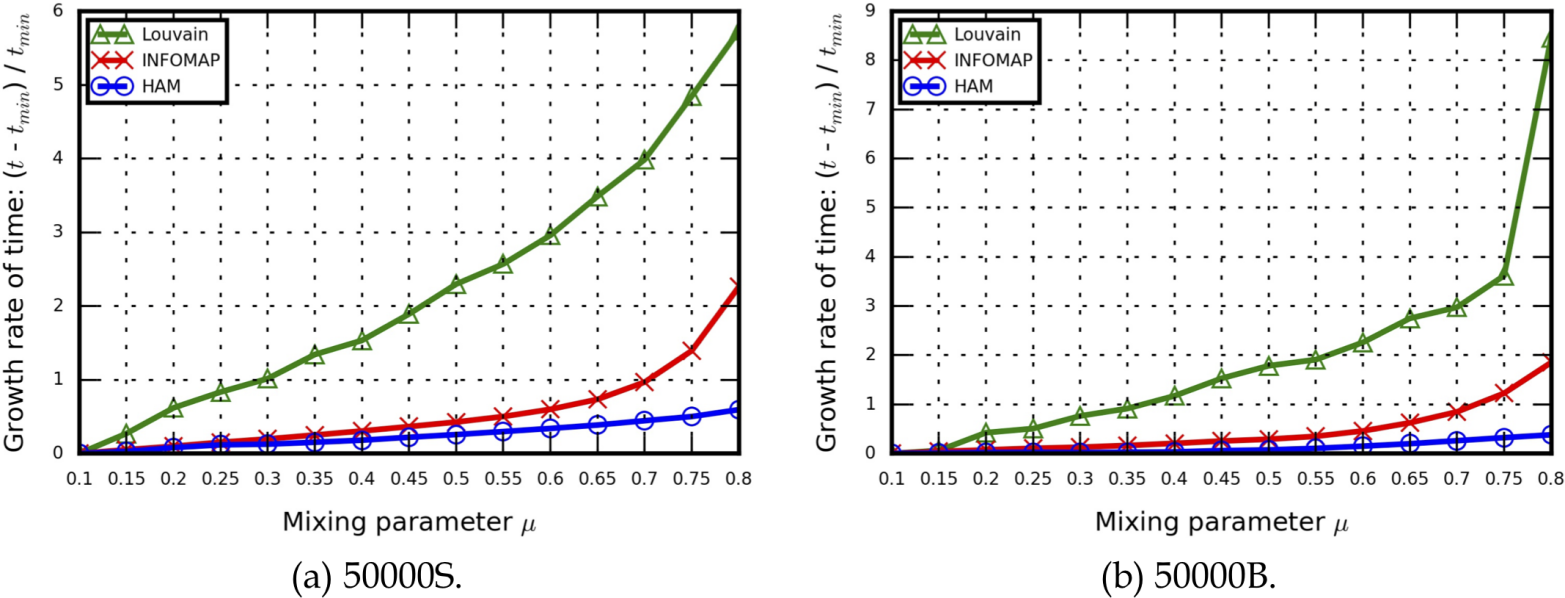
Execution time growth rate results for the LFR benchmark networks used in this study. (a) 50000S, (b) 50000B.

Regarding the growth rate of execution time results (i.e., [*t* − *t*_*min*_]/*t*_*min*_), HAM exhibited good stability in performance growth compared to the Louvain method when *u* = 0.1∼0.8. Infomap data indicate rapid growth when *u* ≥ 0.6 (Fig 7). According to these findings, the HAM algorithm was not significantly affected by small/large community sizes or distinct/indistinct community structures. In contrast, the Louvain method and Infomap were affected by increased *u* values. Although the Louvain method had the best performance efficiency, its execution time growth rate increased quickly, producing execution time results that were close to those produced by the HAM algorithm (e.g., *u* = 0.8 for the 10000B and 50000B LFR benchmark networks).

Data for the scalability criterion are shown in Table 4. Modularity and execution time results for each method represent averages for 10 runs. Overall, only the Louvain method and HAM could be applied to all of the large-scale real-world networks. Further, the Louvain method produced the best modularity results for large-scale real-world networks such as Email-contacts, Loc-gowalla, Web-google, and Wiki-talk for identifying community structures in the absence of ground-truth communities. According to the cosine similarity measure, HAM produced the best modularity results for the Brightkite, Com-youtube, and Com-amazon networks, and was second best for large-scale real-world networks (S7 File). HAM modularity results were close to or better than those produced by the Louvain method. In terms of execution time, the Louvain method had the best performance efficiency for large-scale real-world networks. In terms of performance efficiency, HAM was second, behind the Louvain method. Its performance was considered satisfactory, despite the drawback that HAM was created with an interpreted language (Table B in S7 File).

**Table 4:** Large scale real network results.

Network	Email-contacts			Brightkite			Com-youtube			Com-amazon		
Measure	Q	T-avg	T-std	Q	T-avg	T-std	Q	T-avg	T-std	Q	T-avg	T-std
Louvain	**0.6875**	**0.1295**	0.0122	***0*.*6609***	**0.8330**	0.0125	0.0123	**0.9188**	0.0323	0.0000	**6.4132**	0.1611
CNM	0.6590	10.2258	1.2981	0.5963	328.2683	5.2576	***0*.*5792***	497.8858	1.7927	-	-	-
DS	0.3656	1.4430	0.0174	0.4880	58.1397	0.2177	0.2869	81.1186	0.3387	***0*.*7983***	1132.5838	5.0705
INFOMAP	0.1099	2.0124	0.0327	0.4000	33.4121	0.0707	0.4568	35.4838	0.1492	0.4348	232.5652	0.6746
HAM_Cosine_	***0*.*6691***	***0*.*1654***	0.0076	**0.6631**	***2*.*7394***	0.0651	**0.5926**	***4*.*3555***	0.0548	**0.8980**	***14*.*3317***	0.0550
Network	Com-DBLP			Loc-gowalla			Web-google			Wiki-talk		
Measure	Q	T-avg	T-std	Q	T-avg	T-std	Q	T-avg	T-std	Q	T-avg	T-std
Louvain	***0*.*8083***	**5.3992**	0.0465	**0.6845**	**3.3852**	0.0242	**0.9774**	**19.9524**	0.3932	**0.5837**	**23.5389**	0.4411
CNM	-	-	-	-	-	-	-	-	-	-	-	-
DS	0.6459	798.4390	8.0639	0.4393	855.8750	4.2060	*	*	*	*	*	*
INFOMAP	**0.8131**	124.5974	0.2805	0.5534	152.0472	0.3748	*	*	*	*	*	*
HAM_Cosine_	0.7947	***14*.*3941***	0.0335	***0*.*6825***	***13*.*3427***	0.0931	***0*.*9713***	***55*.*8731***	0.0947	***0*.*5235***	***66*.*1238***	0.2340

**Bold**, best result.

**Bold** and *italics*, second-best result.

Subscript, similarity measure used in HAM.

-, method has excessive time requirement (runs exceed at least one hour for a single time).

*, memory allocation error (e.g., “std::bad_alloc”).

We also conducted multi-resolution analyses to compare HAM performance with minimum, cosine, and Jaccard index similarity measures for small-scale social networks [62–67]. To execute a multi-resolution analysis, we introduced a tunable parameter as suggested by Xiang et al. [68] and Arenas et al. [69]. In the original network phase, a weighted-edge is determined by edge weight *w*_*ij*_ > 0. We substituted a weight threshold for the 0 in formula 7—that is, *E*^*W*^ = {*e*_*ij*_ | *w*_*ij*_ > *w*_*threshold*_}. Hence, edges were classified as weighted when their weights exceeded a threshold, otherwise they were classified as bridge-edge or sink-edge.

**Fig 8:**
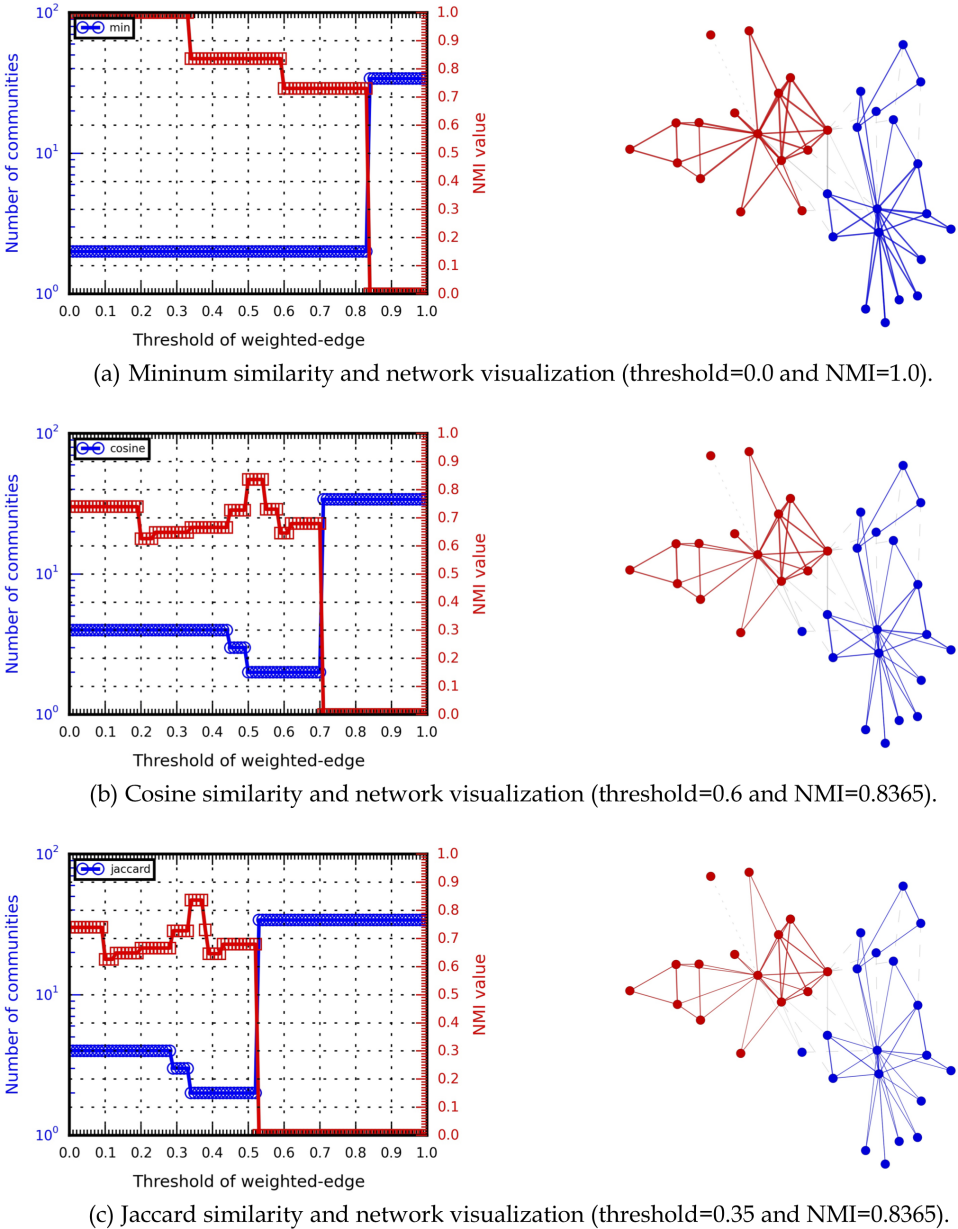
Multi-resolution analysis data for different Karate network similarities.

**Fig 9:**
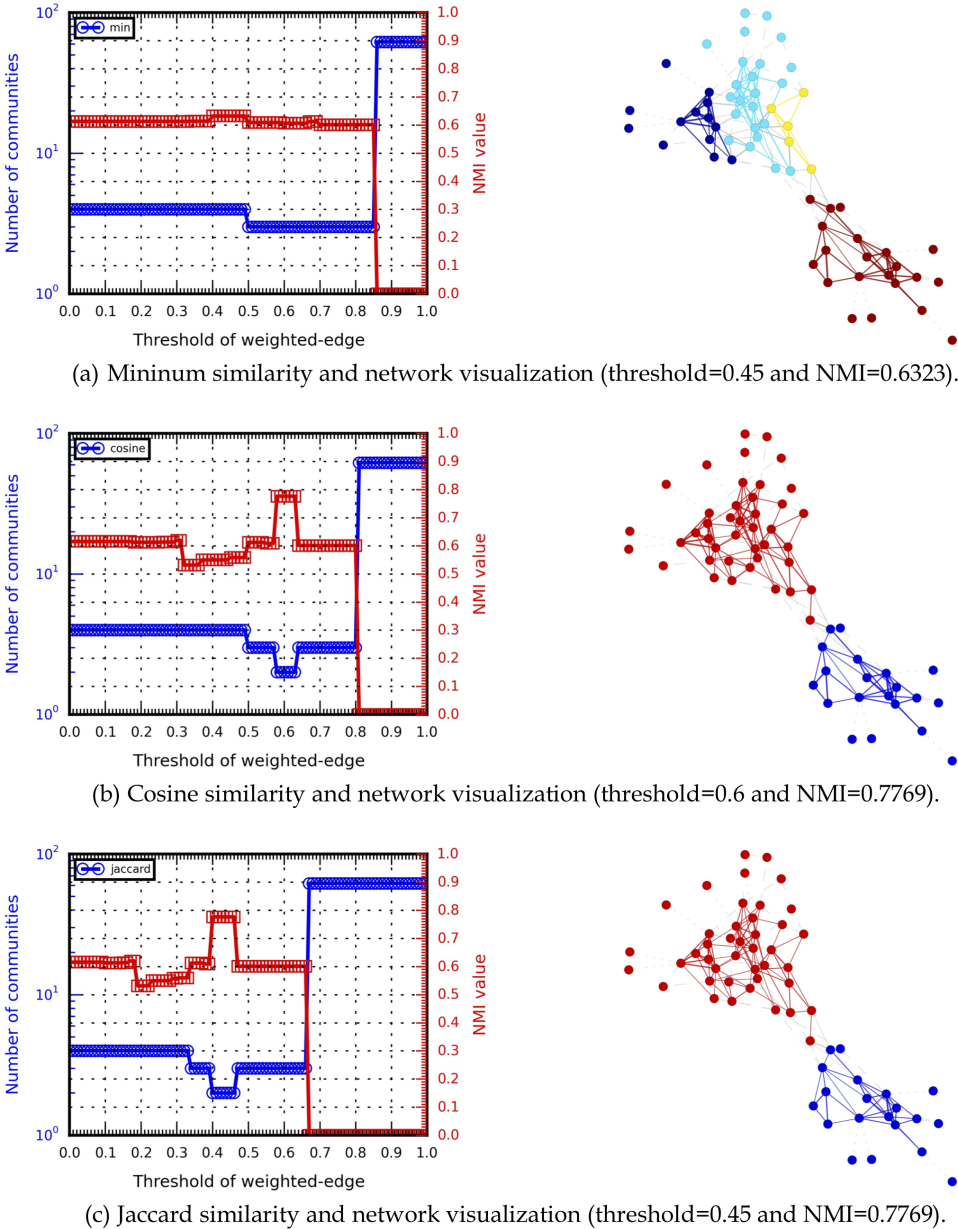
Multi-resolution analysis of different Dolphins network similarities.

In addition to using a weight threshold as a tunable parameter [68–69], we introduced several communities and NMI values, and visualized the identified community structures. The weight threshold value was established as *w*_*threshold*_ ∈ [0,1], in increments of 0.01. Community detection results produced by the HAM algorithm were collected for each threshold value. The results shown in Figs 8 and 9 indicate that different similarity measures affected HAM’s community detection capabilities (e.g., different NMI curve trends). For example, the highest NMI value for the Karate network (1.0) involved the minimum similarity measure, indicating that the identified and ground-truth community structures were identical. In contrast, the highest NMI values for the Dolphins network (0.7769) were produced by both the cosine similarity measure and the Jaccard index. The results also indicate that multi-resolution analysis can be used to determine appropriate parameters (e.g., weight thresholds or tunability) for acquiring useable community detection results. Multi-resolution analysis results for small-scale social networks are shown in S8 File.

## Summary and discussion

The underlying HAM rationale is based on observations from two kinds of synthetic networks and related studies of resolution limit problems associated with community detection [51]. Since modularity approaches are not capable of identifying communities below certain scales, there is a possibility that such communities are connected by single bridges or small numbers of low-weight edges that can be identified via one or more combinations of a similarity measure and rule-based strategies. Further, a similarity measure can be used to calculate edge weights in order to identify low-weight edges, including those located between communities. The best rule-based strategy for merging nodes into super-nodes or for retaining nodes in their own communities is determined by edge type—weighted, bridge, or sink. We therefore designed a pre-processing original-network phase for partitioning networks into sub-communities based on weighted network structure. Because of the modularity maximization mechanism, these sub-communities are merged.

The rule-based methods described in this paper focus on the use of network structure information to extract important features from community structures. This observation can be used to build corresponding arc-merging strategies. For example, in the original HAM network phase we designed a three-step procedure for partitioning network community structures: edge classification for identifying different edge types, edge sorting for determining the order of applying arc-merging strategies, and arc-merging strategies for merging edge endpoints according to edge type and sorting order. All three steps are based on community structure observations. Other researchers have used similar strategies to determine community structure identification start points [40, 47, 63].

State-of-the-art methods can be analyzed, simplified and utilized based on their respective advantages to create explicit rule-based strategies for community detection tasks. In cases of overlapping communities [63–64], overlaps can be used to create corresponding rule-based strategies for HAM extensions. The k-cliques of any given network can be merged as super-node seeds during the original network phase, after which edge endpoints are merged into super-nodes. In some instances of overlap, link-pair similarities can be utilized for HAM extensions [65], and merged link-pair endpoints can serve as super-node seeds during initial network phases. Afterwards, single edge endpoints can be merged into super-nodes. Nodes belonging to multiple communities can be handled by a rule-based strategy involving the creation of duplicate nodes in individual communities.

According to our experimental data, there are two possible explanations for the capability of the proposed HAM algorithm to mitigate resolution limit problems: (a) the similarity measure used to calculate edge weights (especially bridge edges), and (b) the strategy of using bridge edges to maintain community structures. For example, in the Clique rings network, an individual clique is connected to two smaller cliques via two individual bridge edges; a single bridge edge also connects the smaller cliques to each other. Based on this example, we think that any two communities (or groups or cliques) connected by bridge edges should be retained, since merging them into new communities might increase overall modularity value.

Our execution time data indicate that the performance efficiency of HAM was satisfactory, raising questions about which modifications could lead to improvement. Possibilities include the addition of a more efficient sorting algorithm (e.g., a distributed sorting algorithm); a similarity measure with lower computation costs such as a minimum similarity denominator (as opposed to the Jaccard index denominator); simplifying defined rule structures in order to reduce computation costs; and using compiled programming languages such as C and C++ rather than interpreted languages such as Python.

Modularity, which is frequently used to evaluate community detection results produced by algorithms, is strongly associated with resolution limit problems. Alternatives include greedy surprise maximization [35], preprocessing [66], edge-reweighting [67–68], multi-resolution [69], and Hamiltonians [70–71], among others. However, each alternative has its own problems, including excessive community splitting. Infomap, a non-modularity method, uses map equations as quality functions for community detection tasks. Our experimental data indicate that Infomap mitigates the resolution limit problem for clique-pair networks but not for clique-ring networks—a unique resolution limit problem.

**Fig 10:**
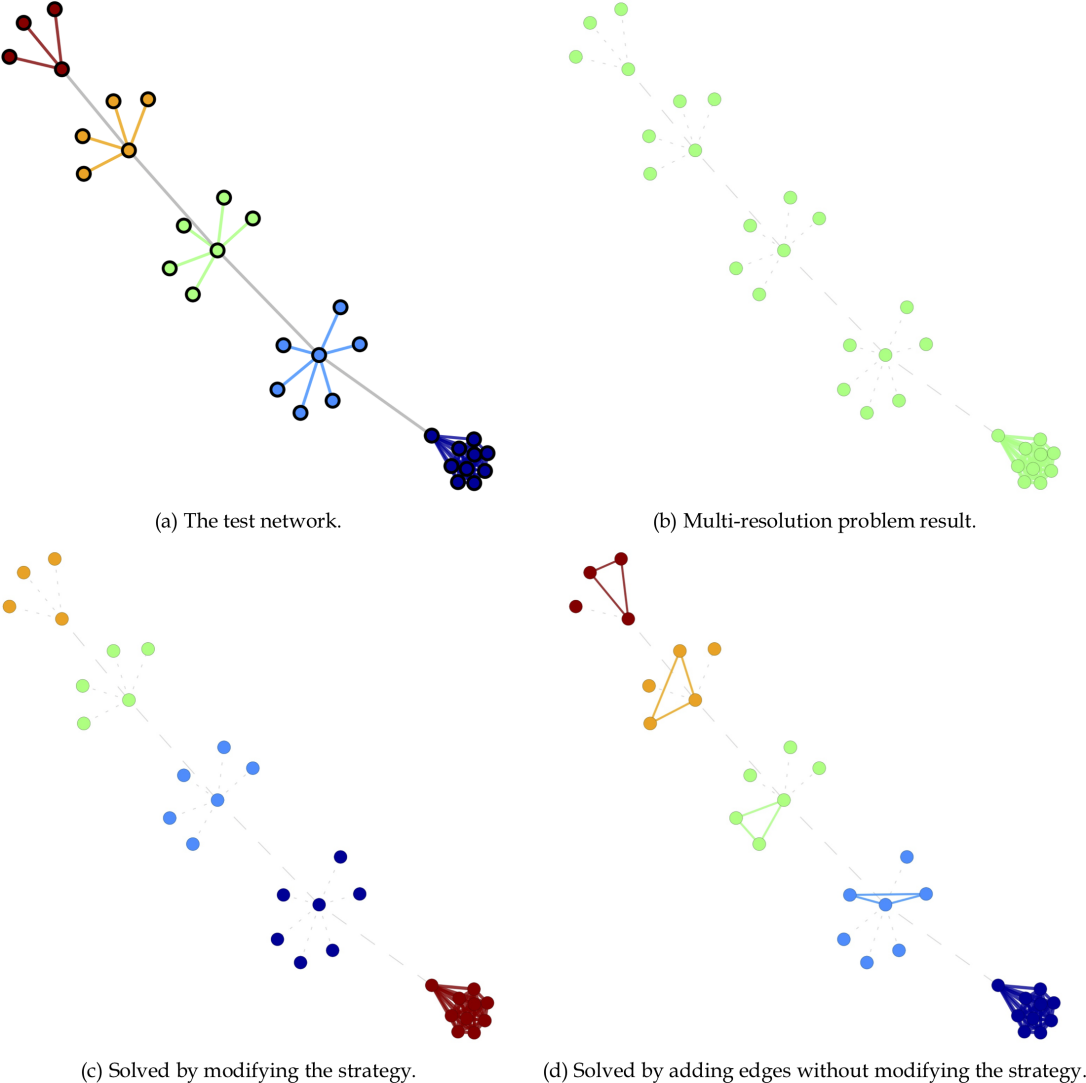
The HAM multi-resolution problem. (a) The test network; (b) multi-resolution problem result; (c) modified strategy solution; (d) solution involving the addition of edges without modifying the strategy.

According to our multi-resolution analysis results (Fig 10), HAM tended to “over-merge” long chains of star-like nodes (communities) connected by bridge edges in Clique line networks [41, 50]. We believe this problem is associated with the structure maintenance strategy (Algorithm A2 in S1 File). After handling weighted edges, a long chain consisting of four star-like nodes and bridge edges establishes a connection to the largest community in the Clique line network (i.e., nodes with four bridge edges and multiple sink edges). After applying the structure maintenance strategy, the first bridge edge is merged according to rule R3, since one endpoint is in an identified community. Subsequently, the other three bridge edges merge with the largest R3-based community. The remaining sink edges are handled by applying the sink-shrinking strategy (Algorithm A3 in S1 File). In the end, only one community is identified.

There are at least three ways to address this problem. First, structure maintenance rules (especially R3 and R4) can be modified to retain edges between a community and a star-like node, or between two star-like nodes. Second, each star-like node can be modified so that only single edges are added for purposes of connecting neighbor pairs in a manner that eliminates the need to modify the structure maintenance strategy. A third possibility is to add node-merging strategies to identify star-like and other node types; this idea exceeds the scope of arc-merging strategies addressed in this paper.

According to the study results shown in Table 5, decisions regarding which algorithm to use—Louvain, Infomap, DS or HAM—must be made on a case-by-case basis. If effectiveness involving small-scale social networks is the main concern, the Louvain method is a better choice for modularity optimization, while HAM is a better choice when accuracy or time limitations are priorities. When the primary concern is mitigating resolution limit problems (examination criterion), DS or HAM are the best choices. For the correctness criterion using LFR theoretical networks, the Louvain method may be preferred due to its smaller time requirement, Infomap if accuracy is emphasized, or HAM in scenarios involving combined accuracy and performance efficiency. For the scalability criterion involving large-scale real-world networks, the Louvain method remains the best choice in terms of community structure quality and execution time. However, HAM is a satisfactory alternative in terms of community structure quality or performance efficiency, especially when avoiding potential resolution limit problems is a primary concern.

**Table 5:** Experiment results summary.

Criterion	(a) Effectiveness			(b) Examination		(c) Correctness		(d) Scalability	
Network	Social networks			Synthetic networks		LFR networks		Large networks	
Measure	NMI	Q	Time	Test1	Test2	NMI	Time	Q	Time
Louvain	-	●	○	-	-	-	●	●	●
CNM	-	-	-	-	-	-	-	-	-
DS	-	-	-	●	●	-	-	-	-
INFOMAP	○	○	-	-	●	●	-	-	-
HAM	●	-	●	●	●	○	○	○	○

●, first place or overcame resolution limitation problem.

○, second place.

## Conclusion

In this paper we introduced rule-based strategies for community detection tasks, and described a hierarchical arc-merging (HAM) algorithm that uses network topologies and rule-based arc-merging strategies to identify community structures. The HAM architecture consists of similarity measurement and modularity optimization phases, plus rule-based strategies for community detection. We also used four criteria—effectiveness, examination, correctness, and scalability—to determine community detection algorithm appropriateness. Experiments were conducted to examine our proposed HAM algorithm according to these criteria, which we believe all community detection algorithms should satisfy. To test for effectiveness, we used five social networks and one small-scale LFR benchmark network, all with ground-truth communities. Our results indicate that HAM was capable of identifying community structures with satisfactory NMI values, and that the identified communities were similar to ground-truth communities in social and LFR benchmark networks. For the examination criterion, our results (involving two synthetic networks) indicate an absence of HAM-associated resolution limit problems. For the correctness criterion, results from an analysis involving LFR benchmark networks (also with ground-truth communities) with different parameters and community sizes indicate that HAM’s NMI values and performance efficiency were as satisfactory as those produced by Infomap. For the scalability criterion, eight large/very large real networks without ground-truth communities were used for separate tests. Results indicate that HAM produced satisfactory modularity values and good performance efficiency, although greater efficiency can likely be achieved if a compiled language is used for implementation.

## Supporting information

S1 File Rule-based strategies for the HAM algorithm. (DOCX)

S2 File Step-by-step example of HAM community detection. (DOCX)

S3 File HAM time complexity analysis. (DOCX)

S4 File Similarity comparison for LFR-benchmark networks. (DOCX)

S5 File NMI results for LFR benchmark networks. (DOCX)

S6 File Execution time results for LFR benchmark networks. (DOCX)

S7 File Summarized results for large-scale real networks. (DOCX)

S8 File Multi-resolution analysis of different similarities for small-scale networks. (DOCX)
