# Supplementary material for: A community detection algorithm using network topologies and rule-based hierarchical arc-merging strategies
Source: PLoS One. 2017 Nov 9;12(11):e0187603. doi: 10.1371/journal.pone.0187603 (PMC5679540; doi:10.1371/journal.pone.0187603)
Supplement: S1 File — (DOCX) [file pone.0187603.s001.docx]

**S1 File. Rule-based strategies for HAM algorithm.**

| **Algorithm S1-1. Community-creating strategy during original network phase.** |
| --- |
| **Input**: network $G$, super-node network $H$, sorted edges $\hat{E}$.  **Output**: super-node network $H$.  1: **For** $e_{ij}=\left( v_{i},v_{j} \right)$ in $\hat{E}$:  2: **If** node $v_{i}$ is not unmerged:  3: **If** node $v_{j}$ is not unmerged (R1):  4: add a super-node $sn$ to $H$ that merges nodes $v_{i}$ and $v_{j}$  5: record nodes $v_{i}$ and $v_{j}$ as member nodes of super-node $sn$  6: assign $l_{sn}=1$ and $d_{sn}=d_{i}+d_{j}$  7: **Else** (R2):  8: merge node $v_{i}$ into super-node ${sn}_{j}$ of node $v_{j}$  9: record node $v_{i}$ as a member node of super-node ${sn}_{j}$  10: assign $l_{{sn}_{j}}=l_{{sn}_{j}}+1$ and $d_{{sn}_{j}}=d_{{sn}_{j}}+d_{i}$  11: **Else**:  12: **If** node $v_{j}$ is not unmerged (R3):  13: merge node $v_{j}$ into super-node ${sn}_{i}$ of node $v_{i}$  14: record node $v_{j}$ as a member node of super-node ${sn}_{i}$  15: assign $l_{{sn}_{i}}=l_{{sn}_{i}}+1$ and $d_{{sn}_{i}}=d_{{sn}_{i}}+d_{j}$  16: **Else** (R5):  17: get super-node ${sn}_{i}$ of node $v_{i}$  18: get super-node ${sn}_{j}$ of node $v_{j}$  19: **If** ${sn}_{i}\neq{sn}_{j}$:  20: **If** $H$ has an edge $e_{{sn}_{i}{sn}_{j}}=\left( {sn}_{i},{sn}_{j} \right)$:  21: assign edge count $\left\vert e_{{sn}_{i}{sn}_{j}} \right\vert=\left\vert e_{{sn}_{i}{sn}_{j}} \right\vert+1$  22: assign edge weight $w_{{sn}_{i}{sn}_{j}}=w_{{sn}_{i}{sn}_{j}}+w_{ij}$  23: **Else**:  24: add edge $e_{{sn}_{i}{sn}_{j}}$ to $H$  25: assign edge count $\left\vert e_{{sn}_{i}{sn}_{j}} \right\vert=1$  26: assign edge weight $w_{{sn}_{i}{sn}_{j}}=w_{ij}$  27: **Else**:  28: assign $l_{sn}=l_{sn}+1$  29: **Return** $H$ |

| **Algorithm S1-2. Structure maintenance strategy during original network phase.** |
| --- |
| **Input**: network $G$, super-node network $H$, sorted edges $\hat{E}$.  **Output**: super-node network $H$.  1: **For** $e_{ij}=\left( v_{i},v_{j} \right)$ in $\hat{E}$:  2: **If** node $v_{i}$ is not unmerged:  3: **If** node $v_{j}$ is not unmerged (R4):  4: add super-node ${sn}_{i}$ to $H$ that merges node $v_{i}$  5: add super-node ${sn}_{j}$ to $H$ that merges node $v_{j}$  6: record node $v_{i}$ as a member node of super-node ${sn}_{i}$  7: record node $v_{j}$ as a member node of super-node ${sn}_{j}$  8: assign $l_{{sn}_{i}}=0$ and $d_{{sn}_{i}}=d_{i}$  9: assign $l_{{sn}_{j}}=0$ and $d_{{sn}_{j}}=d_{j}$  10: add edge $e_{{sn}_{i}{sn}_{j}}=\left( {sn}_{i},{sn}_{j} \right)$ to $H$  11: assign edge count $\left\vert e_{{sn}_{i}{sn}_{j}} \right\vert=1$  12: assign edge weight $w_{{sn}_{i}{sn}_{j}}=w_{ij}$  13: **Else** (R2):  14: repeat S1-1  15: **Else**:  16: **If** node $v_{j}$ is not unmerged (R3):  17: repeat S1-1  18: **Else** (R5):  19: repeat S1-1  20: **Return** $H$ |

| **Algorithm S1-3. Sink-shrinking strategy during original network phase.** |
| --- |
| **Input**: network $G$, super-node network $H$, sorted edges $\hat{E}$.  **Output**: super-node network $H$.  1: **For** $e_{ij}=\left( v_{i},v_{j} \right)$ in $\hat{E}$:  2: **If** node $v_{i}$ is not unmerged:  3: **If** node $v_{j}$ is not unmerged:  4: pass  5: **Else** (R2):  6: repeat S1-1  7: **Else**:  8: **If** node $v_{j}$ is not unmerged (R3):  9: repeat S1-1  10: **Else** (R5):  11: pass  12: **Return** $H$ |

| **Algorithm S1-4. Community-creating strategy during super-node network phase.** |
| --- |
| **Input**: super-node network$G$, super-node network $H$, sorted edges $\hat{E}$.  **Output**: super-node network $H$.  1: **For** $e_{ij}=\left( v_{i},v_{j} \right)$ in $\hat{E}$:  2: **If** node $v_{i}$ is not unmerged:  3: **If** node $v_{j}$ is not unmerged (R1):  4: add a super-node $sn$ to $H$ that merges node $v_{i}$ and $v_{j}$  5: record nodes $v_{i}$ and $v_{j}$ as member nodes of super-node $sn$  6: assign $l_{sn}=l_{i}+l_{j}+\left\vert e_{ij} \right\vert$ and $d_{sn}=d_{i}+d_{j}$  7: **Else** (R2):  8: get super-node ${sn}_{i}$ of node $v_{i}$  9: **If** re-checked ${\Delta Q}_{ij}$ is still larger than zero:  10: merge node $v_{j}$ to super-node ${sn}_{i}$ of node $v_{i}$  11: record node $v_{j}$ as a member node of super-node ${sn}_{i}$  12: assign $l_{{sn}_{i}}=l_{{sn}_{i}}+l_{j}+\left\vert e_{ij} \right\vert$ and $d_{{sn}_{i}}=d_{{sn}_{i}}+d_{j}$  13: **Else**:  14: add a super-node ${sn}_{j}$ to $H$ that merges node $v_{j}$  15: record node $v_{j}$ as a member node of super-node ${sn}_{j}$  16: assign $l_{{sn}_{j}}=l_{j}$ and $d_{{sn}_{j}}=d_{j}$  17: **If** $H$ has an edge $e_{{sn}_{i}{sn}_{j}}=\left( {sn}_{i},{sn}_{j} \right)$:  18: assign edge count $\left\vert e_{{sn}_{i}{sn}_{j}} \right\vert=\left\vert e_{{sn}_{i}{sn}_{j}} \right\vert+\left\vert e_{ij} \right\vert$  19: assign edge weight $w_{{sn}_{i}{sn}_{j}}=w_{{sn}_{i}{sn}_{j}}+w_{ij}$  20: **Else**:  21: add an edge $e_{{sn}_{i}{sn}_{j}}$ to $H$  22: assign edge count $\left\vert e_{{sn}_{i}{sn}_{j}} \right\vert=\left\vert e_{ij} \right\vert$  23: assign edge weight $w_{{sn}_{i}{sn}_{j}}=w_{ij}$  24: **Else**:  25: **If** node $v_{j}$ is not unmerged (R3):  26: get super-node ${sn}_{j}$ of node $v_{j}$  27: **If** re-checked ${\Delta Q}_{ij}$ is still larger than zero:  28: merge node $v_{i}$ to super-node ${sn}_{j}$ of node $v_{j}$  29: record node $v_{i}$ as a member node of super-node ${sn}_{j}$  30: assign $l_{{sn}_{j}}=l_{{sn}_{j}}+l_{i}+\left\vert e_{ij} \right\vert$ and $d_{{sn}_{j}}=d_{{sn}_{j}}+d_{i}$  31: **Else**:  32: add a super-node ${sn}_{i}$ to $H$ that merges node $v_{i}$  33: record node $v_{i}$ as a member node of super-node ${sn}_{i}$  34: assign $l_{{sn}_{i}}=l_{i}$ and $d_{{sn}_{i}}=d_{i}$  35: **If** $H$ has an edge $e_{{sn}_{i}{sn}_{j}}=\left( {sn}_{i},{sn}_{j} \right)$:  36: assign edge count $\left\vert e_{{sn}_{i}{sn}_{j}} \right\vert=\left\vert e_{{sn}_{i}{sn}_{j}} \right\vert+\left\vert e_{ij} \right\vert$  37: assign edge weight $w_{{sn}_{i}{sn}_{j}}=w_{{sn}_{i}{sn}_{j}}+w_{ij}$  38: **Else**:  39: add an edge $e_{{sn}_{i}{sn}_{j}}$ to $H$  40: assign edge count $\left\vert e_{{sn}_{i}{sn}_{j}} \right\vert=\left\vert e_{ij} \right\vert$  41: assign edge weight $w_{{sn}_{i}{sn}_{j}}=w_{ij}$  42: **Else** (R5):  43: get super-node ${sn}_{i}$ of node $v_{i}$  44: get super-node ${sn}_{j}$ of node $v_{j}$  45: **If** ${sn}_{i}\neq{sn}_{j}$:  46: **If** $H$ has edge $e_{{sn}_{i}{sn}_{j}}=\left( {sn}_{i},{sn}_{j} \right)$:  47: assign edge count $\left\vert e_{{sn}_{i}{sn}_{j}} \right\vert=\left\vert e_{{sn}_{i}{sn}_{j}} \right\vert+1$  48: assign edge weight $w_{{sn}_{i}{sn}_{j}}=w_{{sn}_{i}{sn}_{j}}+w_{ij}$  49: **Else**:  50: add edge $e_{{sn}_{i}{sn}_{j}}$ to $H$  51: assign edge count $\left\vert e_{{sn}_{i}{sn}_{j}} \right\vert=1$  52: assign edge weight $w_{{sn}_{i}{sn}_{j}}=w_{ij}$  53: **Else**:  54: assign $l_{sn}=l_{sn}+1$  55: **Return** $H$ |

| **Algorithm S1-5. Structure maintenance strategy during super-node network phase.** |
| --- |
| **Input**: network $G$, super-node network $H$, sorted edges $\hat{E}$.  **Output**: super-node network $H$.  1: **For** $e_{ij}=\left( v_{i},v_{j} \right)$ in $\hat{E}$:  2: get super-node ${sn}_{i}$ of node $v_{i}$  3: get super-node ${sn}_{j}$ of node $v_{j}$  4: **If** node $v_{i}$ is not unmerged:  5: **If** node $v_{j}$ is not unmerged (R1):  6: add a super-node ${sn}_{i}$ to $H$ that merges node $v_{i}$  7: add a super-node ${sn}_{j}$ to $H$ that merges node $v_{j}$  8: record node $v_{i}$ as a member node of super-node ${sn}_{i}$  9: record node $v_{j}$ as a member node of super-node ${sn}_{j}$  10: assign $l_{{sn}_{i}}=l_{i}$ and $d_{{sn}_{i}}=d_{i}$  11: assign $l_{{sn}_{j}}=l_{j}$ and $d_{{sn}_{j}}=d_{j}$  12: **Else** (R2):  13: get super-node ${sn}_{j}$ of node $v_{j}$  14: add a super-node ${sn}_{j}$ to $H$ that merges node $v_{j}$  15: record node $v_{j}$ as a member node of super-node ${sn}_{j}$  16: assign $l_{{sn}_{j}}=l_{j}$ and $d_{{sn}_{j}}=d_{j}$  17: **Else**:  18: I**f** node $v_{j}$ is not unmerged (R3):  19: get super-node ${sn}_{i}$ of node $v_{i}$  20: add a super-node ${sn}_{j}$ to $H$ that merges node $v_{j}$  21: record node $v_{i}$ as a member node of super-node ${sn}_{i}$  22: assign $l_{{sn}_{j}}=l_{j}$ and $d_{{sn}_{j}}=d_{j}$  23: **Else** (R5):  24: get super-node ${sn}_{i}$ of node $v_{i}$  25: get super-node ${sn}_{j}$ of node $v_{j}$  26: **If** ${sn}_{i}\neq{sn}_{j}$:  27: **If** $H$ has an edge $e_{{sn}_{i}{sn}_{j}}=\left( {sn}_{i},{sn}_{j} \right)$:  28: assign edge count $\left\vert e_{{sn}_{i}{sn}_{j}} \right\vert=\left\vert e_{{sn}_{i}{sn}_{j}} \right\vert+1$  29: assign edge weight $w_{{sn}_{i}{sn}_{j}}=w_{{sn}_{i}{sn}_{j}}+w_{ij}$  30: **Else**:  31: add an edge $e_{{sn}_{i}{sn}_{j}}$ to $H$  32: assign edge count $\left\vert e_{{sn}_{i}{sn}_{j}} \right\vert=1$  33: assign edge weight $w_{{sn}_{i}{sn}_{j}}=w_{ij}$  34: **Else**:  35: assign $l_{sn}=l_{sn}+1$  36: **Return** $H$ |
