# Supplementary material for: A community detection algorithm using network topologies and rule-based hierarchical arc-merging strategies
Source: PLoS One. 2017 Nov 9;12(11):e0187603. doi: 10.1371/journal.pone.0187603 (PMC5679540; doi:10.1371/journal.pone.0187603)
Supplement: S2 File — (DOCX) [file pone.0187603.s002.docx]

**S2 File. Step-by-step example of HAM community detection.**

The toy network shown in Fig S2-1 consists of 13 nodes and 20 edges.


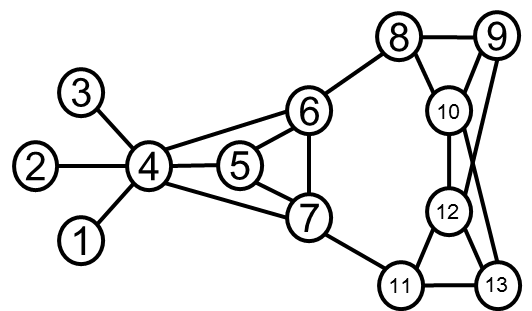


**Fig S2-1. Toy network.**

The minimum similarity measure used in this example is defined as (4) and expressed as

where common neighbor $S_{cn}\left( i, j \right)=\left| \Gamma\left( i \right)\cap\Gamma\left( j \right) \right|$, 𝛤(𝑖) is the neighbor set of node *i*, and 𝛤(𝑗) the neighbor set of node *j*.

Fig S2-2 shows the difference in toy network structure before and after calculating edge weight (similarity)—steps 3 to 7 in algorithm 1. **Bold** and grey lines indicate the different similarity values of edges. Dashed lines indicate that the similarity of an edge equals 0.

| 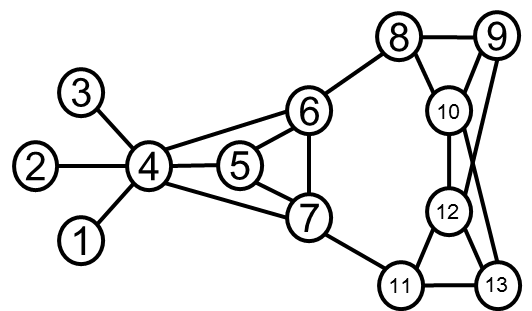 | 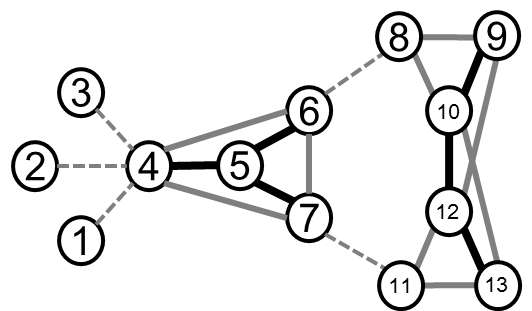 |
| --- | --- |
| (a) Before calculating similarities. | (b) After calculating similarities. |

**Fig S2-2. Similarity differences before and after calculations.** (a) Before calculating similarities, (b) After calculating similarities.

During the original network phase of HAM, toy network edges are classified as weighted-edge $E^{W}$ (**bold**), bridge-edge $E^{B}$ (dashed lines), or sink-edge $E^{S}$ (dotted lines). Edge classification results are shown in Fig S2-3.

| 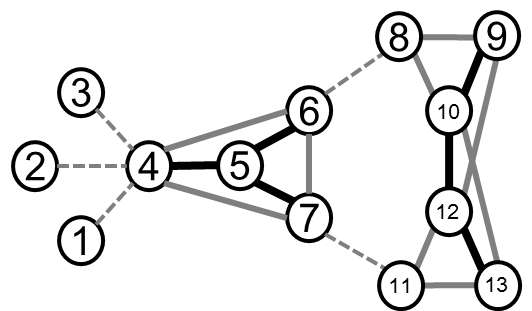 | 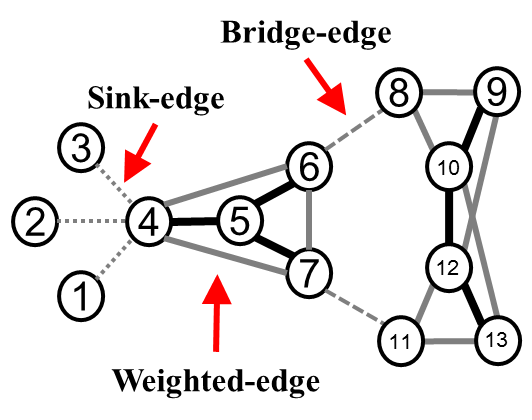 |
| --- | --- |
| (a) Before edge classification. | (b) After edge classification. |

**Fig S2-3. Toy network edge classification results.** (a) Before edge classification, (b) After edge classification.

After edge classification, $E^{W}$ and $E^{B}$ edges are sorted according to two indexes: edge similarity ($w_{ij}$) and the summed degree of two edge endpoints ($k_{i}+k_{j}$), as described in section 2B of our paper. Tables S2-1 and S2-2 present sorted weighted-edge and bridge-edge details; sink-edge $E^{S}$ details are shown in Table S2-3.

**Table S2-1. Details for sorted weighted-edge** $\boldsymbol{E}^{\boldsymbol{W}}$**.**

| $v_{i}$ | $v_{j}$ | $w_{ij}$ | $k_{i}+k_{j}$ | Strategy:Rule |
| --- | --- | --- | --- | --- |
| 4 | 5 | 0.6667 | 9 | S1-1:R1 |
| 5 | 6 | 0.6667 | 7 | S1-1:R3 |
| 5 | 7 | 0.6667 | 7 | S1-1:R3 |
| 9 | 10 | 0.6667 | 7 | S1-1:R1 |
| 12 | 13 | 0.6667 | 7 | S1-1:R1 |
| 4 | 6 | 0.5 | 10 | S1-1:R5 |
| 4 | 7 | 0.5 | 10 | S1-1:R5 |
| 6 | 7 | 0.5 | 8 | S1-1:R5 |
| 10 | 12 | 0.5 | 8 | S1-1:R5 |
| 8 | 10 | 0.3333 | 7 | S1-1:R2 |
| 9 | 12 | 0.3333 | 7 | S1-1:R5 |
| 10 | 13 | 0.3333 | 7 | S1-1:R5 |
| 11 | 12 | 0.3333 | 7 | S1-1:R2 |
| 8 | 9 | 0.3333 | 6 | S1-1:R5 |
| 11 | 13 | 0.3333 | 6 | S1-1:R5 |

**Table S2-2. Details for sorted bridge-edge** $\boldsymbol{E}^{\boldsymbol{B}}$**.**

| $v_{i}$ | $v_{j}$ | $w_{ij}$ | $k_{i}+k_{j}$ | Strategy:Rule |
| --- | --- | --- | --- | --- |
| 6 | 8 | 0 | 7 | S1-2:R5 |
| 7 | 11 | 0 | 7 | S1-2:R5 |

**Table S2-3. Details for sink-edge** $\boldsymbol{E}^{\boldsymbol{S}}$**.**

| $v_{i}$ | $v_{j}$ | $w_{ij}$ | $k_{i}+k_{j}$ | Strategy:Rule |
| --- | --- | --- | --- | --- |
| 1 | 4 | 0 | 7 | S1-3:R2 |
| 2 | 4 | 0 | 7 | S1-3:R2 |
| 3 | 4 | 0 | 7 | S1-3:R2 |

Next, rule-based strategies are applied to the edge classes (step 18 of algorithm 1). Following the edge order shown in Table S2-1, the S1-1 community-creating strategy and R1 are used to create super-node 14 and to merge the edge (4, 5) as shown in Fig S2-4. S1-1 and R3 are then used to merge edges (5, 7) and (6, 7) into the super-node 14 shown in Fig S2-5.

| 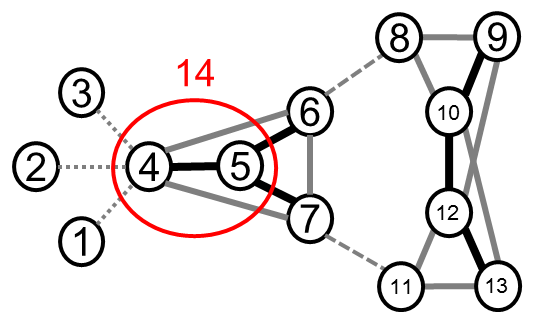 |
| --- |
| **Fig S2-4. Result of merging the edge (4, 5).** |
|  |
| 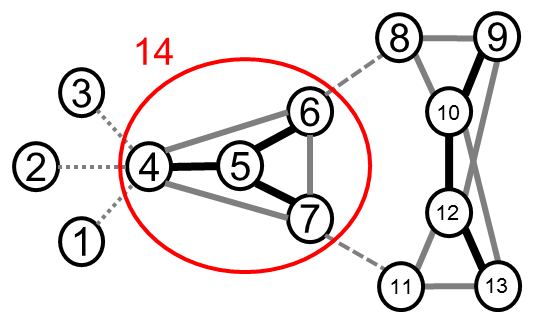 |
| **Fig S2-5. Result of merging edges (5, 7) and (6, 7).** |

Next, S1-1 and R1 are used to create super-node 15 from the merged the edge (9, 10), and to create super-node 16 from the merged the edge (12, 13) (Fig. S2-6).

| 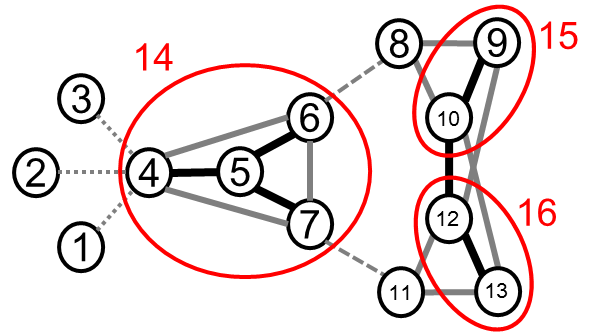 |
| --- |
| **Fig S2-6. Result of merging edges (9, 10) and (12, 13).** |

Next, S1-1 and R5 are used to bypass edges (4, 6), (4, 7), (6, 7) and (10, 12). The result is the same as in Fig S2-6. S1-1 and R2 are then used to merge the edges (8, 10), and S1-1 and R5 are used to bypass edges (9, 12) and (10, 13); results are shown as Fig S2-7.

| 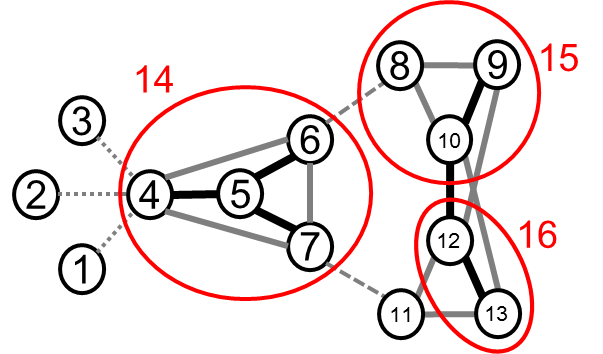 |
| --- |
| **Fig S2-7. Result of merging the edge (9, 10) and bypassing edges (9, 12) and (10, 13).** |

Next, S1-1 and R2 are used to merge the edge (11, 12), and S1-1 and R5 are used to bypass edge (8, 9) and (11, 13). The result following the application of strategy S1-1 is shown as Fig S2-8.

| 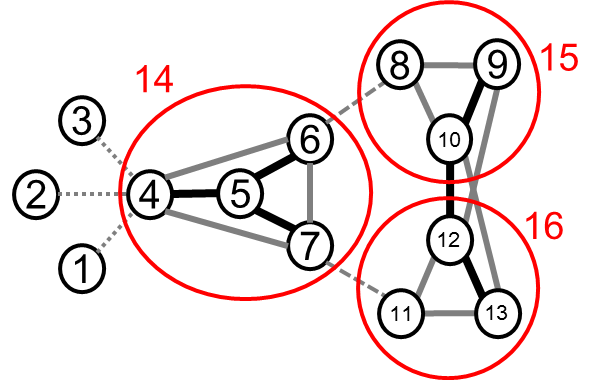 |
| --- |
| **Fig S2-8. Result of merging the edge (11, 12), and bypassing edges (8, 9) and (11, 13).** |

After applying strategy S1-1, the S1-2 structure-maintain strategy is used with the current super-node network (step 19 of algorithm 1). By following the edge order of Table S2-2, S1-2 and R5 are used to bypass edges (6, 8) and (7, 11), with the same results as in Fig S2-8.

After applying strategy S1-2, the S1-3 thread-shrinking strategy is used to merge the sink edges into a super-node (step 20 of algorithm 1). S1-3 and R2 are used to merge edges (1, 4), (2, 4) and (3, 4) into super-node 14. The result after applying S1-3 is shown in Fig S2-9.

| 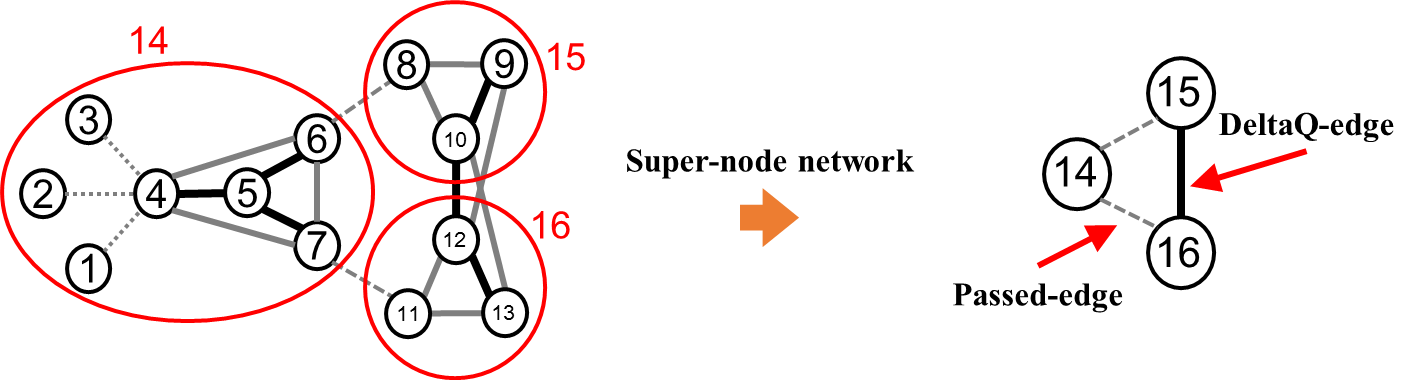 |
| --- |
| **Fig S2-9. Result from merging edges (1, 4), (2, 4) and (3, 4) into the super-node 14.** |

A high-level super-node network is created after the original network phase, after which the algorithm enters the super-node network phase. Edges are classified as deltaQ-edge $E^{\Delta Q}$ or bypass-edge $E^{P}$, indicated as **bold** or dashed lines in Fig S2-9 (step 24 of algorithm 1). The two edge classes are then sorted according to two indexes: edge similarity $w_{ij}$ and ${\Delta Q}_{ij}$ value. Details are shown as Tables S2-4 and S2-5.

**Table S2-4. Details for sorted deltaQ-edge** $\boldsymbol{E}^{\boldsymbol{\Delta Q}}$**.**

| $v_{i}$ | $v_{j}$ | $w_{ij}$ | ${\Delta Q}_{ij}$ | Strategy:Rule |
| --- | --- | --- | --- | --- |
| 16 | 15 | 1.1666 | 0.025 | S1-4:R1 |

**Table S2-5. Details for sorted bypass-edge** $\boldsymbol{E}^{\boldsymbol{P}}$**.**

| $v_{i}$ | $v_{j}$ | $w_{ij}$ | ${\Delta Q}_{ij}$ | Strategy:Rule |
| --- | --- | --- | --- | --- |
| 16 | 14 | 0 | -0.2 | S1-5:R3 |
| 14 | 15 | 0 | -0.2 | S1-5:R5 |

Next, the rule-based strategies are applied to the edge classes (step 27 of algorithm 1). By following the edge order in Table S2-4, the S1-4 community-creating strategy is used to process deltaQ-edge $E^{\Delta Q}$. S1-4 and R1 are used to create super-node 17 and to merge the edge (15, 16), as shown in Fig S2-10. The S1-5 structure-maintain strategy is then applied to bypass-edge $E^{P}$ (step 28 of algorithm 1). S1-5 and R5 are used to bypass edges (14, 16) and (14, 15). The result is shown as Fig S2-11.

| 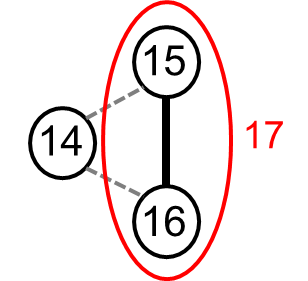 |
| --- |
| **Fig S2-10. Result of merging edges 15 and 16.** |
|  |
| 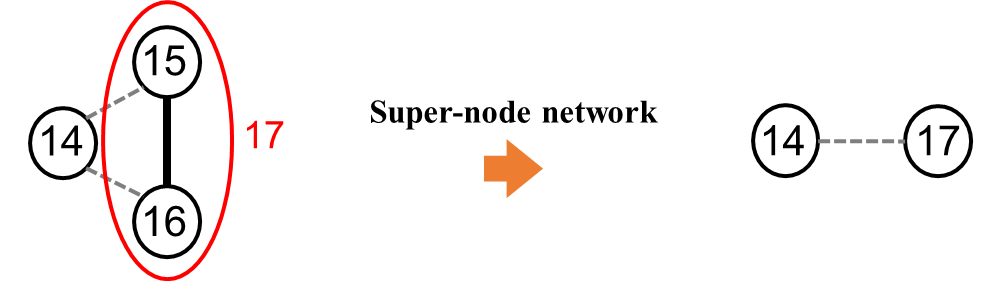 |
| **Fig S2-11. Result of merging the edge (15, 16) and creating a super-node network.** |

Last, after applying HAM to community detection, two high-level toy networks are constructed and super-node network communities are identified. Community results can be projected from the high-level super-node network to the original network—for example, from L2 to L1 of the super-node network, then to the original network (level 0, L0) as shown in Fig S2-12. Fig S2-13 shows the actual projection results for the toy network.

| 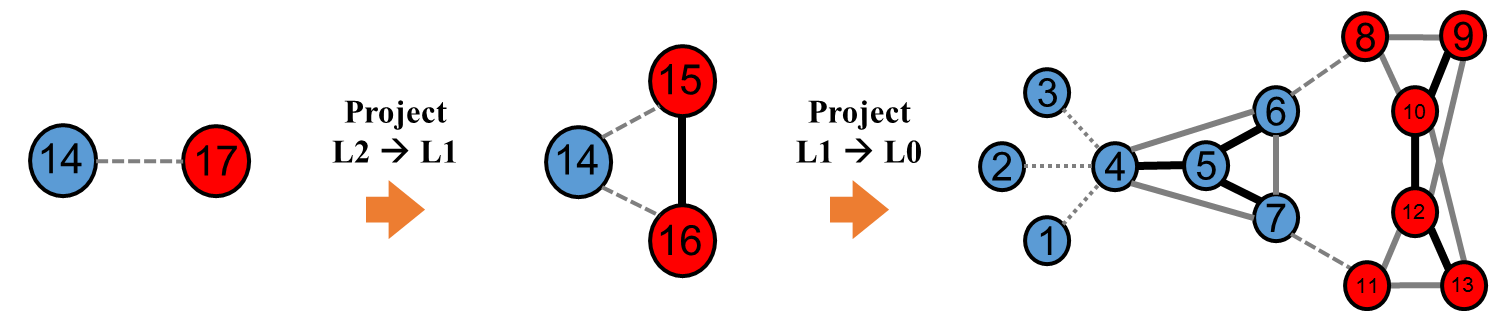 |
| --- |
| **(a) Projecting L2 results to the L1 super-node network and then to the original network.** |
| 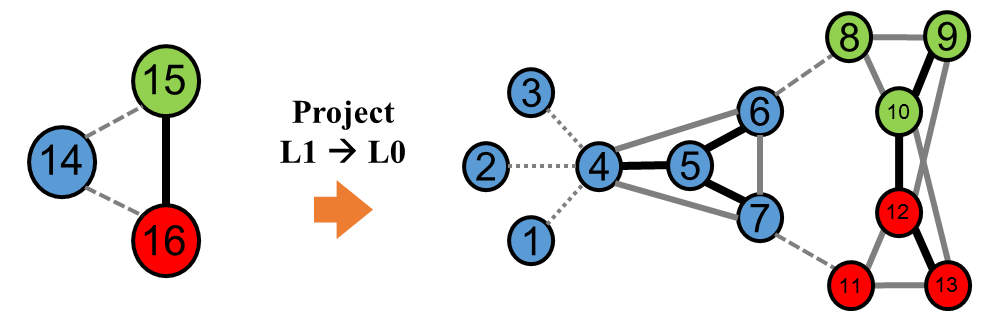 |
| **(b) Projecting L1 results to the original network.** |

**Fig S2-12. Projection results for the super-node networks.** (a) Projecting L2 results to the L1 super-node network and then to the original network, (b) Projecting L1 results to the original network.

| 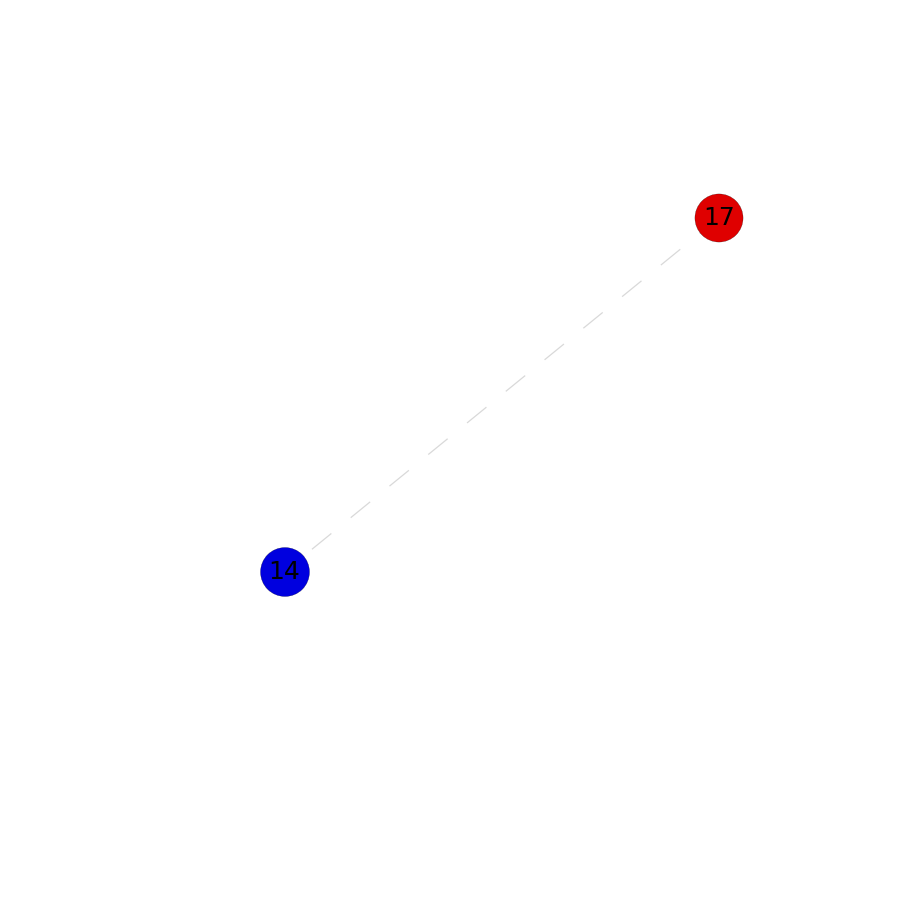 | 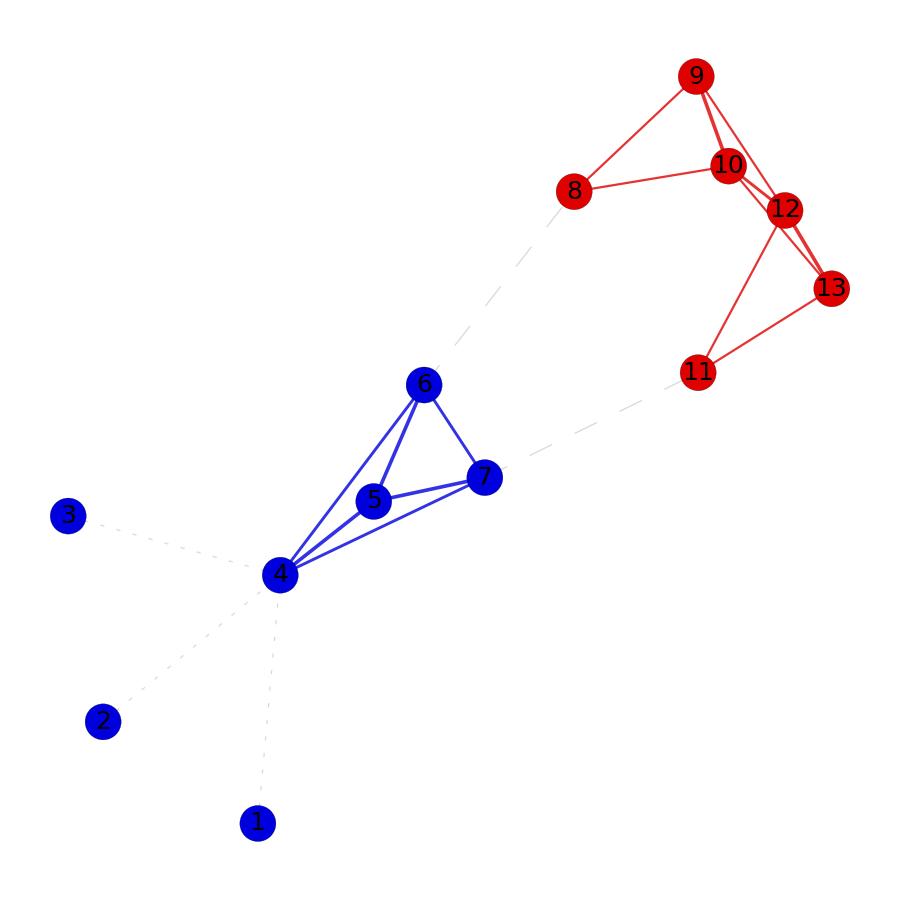 |
| --- | --- |
| **(a) L2 projection results for the original network (L0).** | |
| 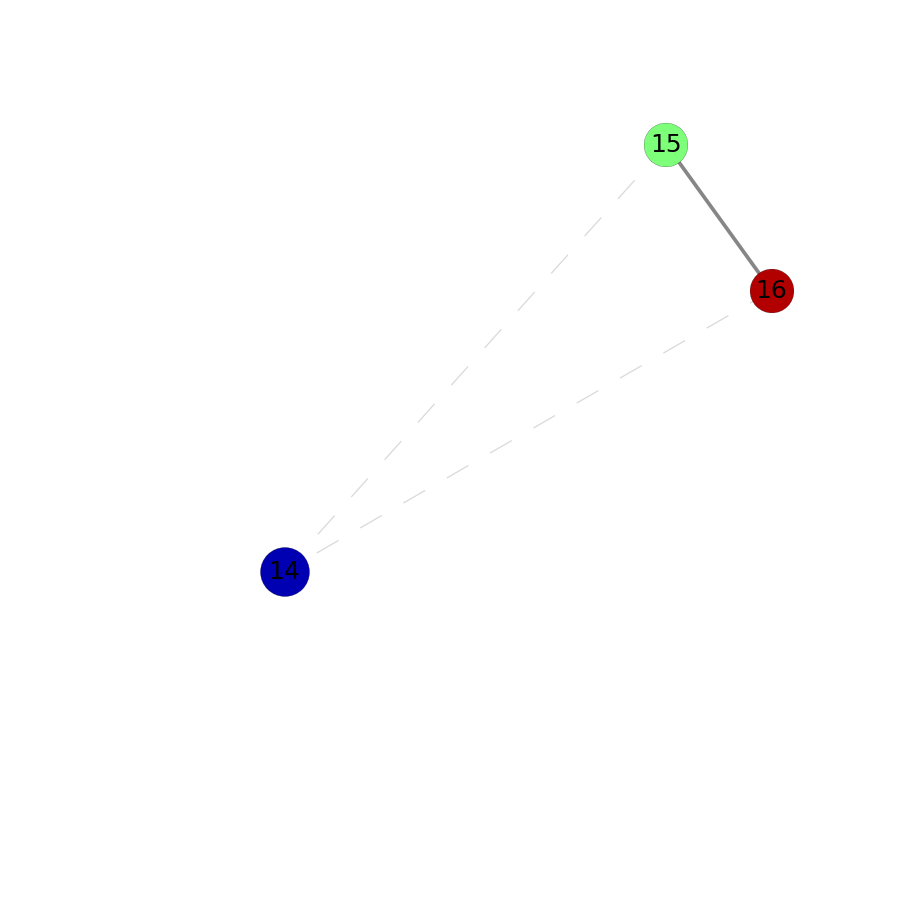 | 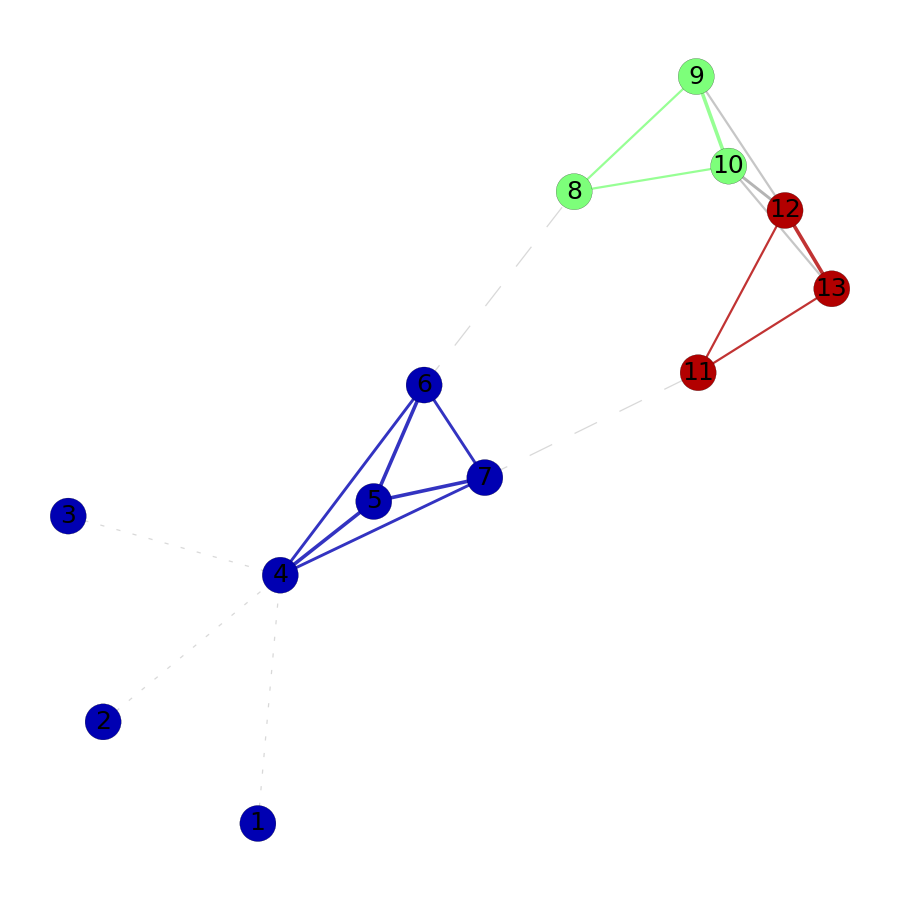 |
| **(b) L1 projection results for the original network (L0).** | |

**Fig S2-13. Actual toy network projection results.** (a) L2 projection results for the original network (L0), (b) L1 projection results for the original network (L0).
