# Supplementary material for: A community detection algorithm using network topologies and rule-based hierarchical arc-merging strategies
Source: PLoS One. 2017 Nov 9;12(11):e0187603. doi: 10.1371/journal.pone.0187603 (PMC5679540; doi:10.1371/journal.pone.0187603)
Supplement: S3 File — (DOCX) [file pone.0187603.s003.docx]

**S3 File. HAM time complexity analysis.**

| **Algorithm 1** Hierarchical Arc-Merging (HAM) Algorithm. | Time complexity |
| --- | --- |
| **Input**: network $G$.  **Output**: network list $L$. |  |
| 1: $NetLevel\leftarrow0$  2: $L\leftarrow[ ]$ | $O\left( 1 \right)$  $O\left( 1 \right)$ |
| 3: **For** $v_{i}$ in $V$:  4: create neighbor set $\Gamma\left( i \right)$ for $v_{i}$  5: calculate node degree $k_{i}$for $v_{i}$ | $O\left( \left\langle k \right\rangle\cdot V \right)$  $O\left( \left\langle k \right\rangle\right)$  $O\left( 1 \right)$ |
| 6: **For** $e_{ij}=\left( v_{i},v_{j} \right)$ in $E$:  7: calculate similarity $w_{ij}$ for $e_{ij}$ | $O\left( E \right)$ |
| 8: append $G$ to network list $L$: $L\leftarrow L+[G]$  9: $NetLevel\leftarrow NetLevel+1$ | $O\left( 1 \right)$  $O\left( 1 \right)$ |
| 10: **While** **True**: | $O\left( L^{'} \right)$  Let $L^{'}=NetLevel$ |
| 11: create an empty super-node network $H$  12: **If** $NetLevel==1$:  13: /* Original HAM network phase */  14: create initial member-node information for $H$ | $O\left( 1 \right)$  $O\left( 1 \right)$  $O\left( V \right)$ |
| 15: classify $E$ into three edge classes: $E^{W}$, $E^{B}$ and $E^{S}$ | $O\left( E+E^{*}logE^{*} \right)$  Let $E^{*}=max\left( E^{W},E^{B} \right)$  Do not sort $E^{S}$ |
| 16: **If** $E^{W}$ is empty:  17: **break** | $O\left( 1 \right)$  $O\left( 1 \right)$ |
| 18: process weighted-edge $E^{W}$ to construct $H$ using S1-1  19: process bridge-edge $E^{B}$ to construct $H$ using S1-2  20: process sink-edge $E^{S}$ to construct $H$ using S1-3 | $O\left( c\cdot E^{W} \right)$  $O\left( c\cdot E^{B} \right)$  $O\left( c\cdot E^{S} \right)$ |
|  | $O\left( c\cdot E \right)$ for 18~20  $E=E^{W}+E^{B}+E^{S}$  $c$ is cost of merging node |
| 21: **Else:**  22: /* HAM super-node network phase */  23: create member node information from $G$ | $O\left( 1 \right)$  $O\left( V^{'} \right)$  Let $V^{'}$ is nodes of $G$ |
| 24: classify $E$ into two edge classes: $E^{\Delta Q}$ and $E^{P}$ | $O\left( E^{'}+E^{\Delta Q}logE^{\Delta Q} \right)$  Let $E^{'}$ is edges of $G$  Do not sort $E^{P}$ |
| 25: **If** $E^{\Delta Q}$ is empty:  26: **break** | $O\left( 1 \right)$  $O\left( 1 \right)$ |
| 27: process deltaQ-edge $E^{\Delta Q}$ to construct $H$ using S1-4  28: process passed-edge $E^{P}$ to construct $H$ using S1-5 | $O\left( c\cdot E^{\Delta Q} \right)$  $O\left( c\cdot E^{P} \right)$ |
|  | $O\left( c\cdot E^{'} \right)$ for 27~28  $E^{'}=E^{\Delta Q}+E^{P}$ |
| 29: refine $H$ member node information  30: calculate $H$ modularity using member node information | $O\left( V^{''} \right)$  $O\left( M \right)$  $V^{''}$ is nodes of $H$  $M$ is community number  $M=V^{''}$ |
| 31: **If** $NetLevel>1$:  32: $\Delta Q=Q^{H}-Q^{G}$  33: **If** $\Delta Q$ is less than threshold:  34: **break**  35: copy $H$ as $G$: $H\leftarrow G$  36: append $G$ to network list $L$: $L\leftarrow L+[G]$  37: $NetLevel\leftarrow NetLevel+1$ | $O\left( 1 \right)$  $O\left( 1 \right)$  $O\left( 1 \right)$  $O\left( 1 \right)$  $O\left( 1 \right)$  $O\left( 1 \right)$  $O\left( 1 \right)$ |
| 38: **Return** $L$ | $O\left( 1 \right)$ |
| Overall time complexity: $O\left( L^{'}\cdot E^{*}logE^{*} \right)$ | |
| Note: $O\left( V \right)<O\left( E \right)<O\left( E^{\Delta Q}logE^{\Delta Q} \right)<O\left( L^{'}\cdot E^{*}logE^{*} \right)<O\left( ElogE \right)$ | |
