# Supplementary material for: A community detection algorithm using network topologies and rule-based hierarchical arc-merging strategies
Source: PLoS One. 2017 Nov 9;12(11):e0187603. doi: 10.1371/journal.pone.0187603 (PMC5679540; doi:10.1371/journal.pone.0187603)
Supplement: S4 File — (DOCX) [file pone.0187603.s004.docx]

**S4 File. Similarity comparison for LFR-benchmark networks.**

| 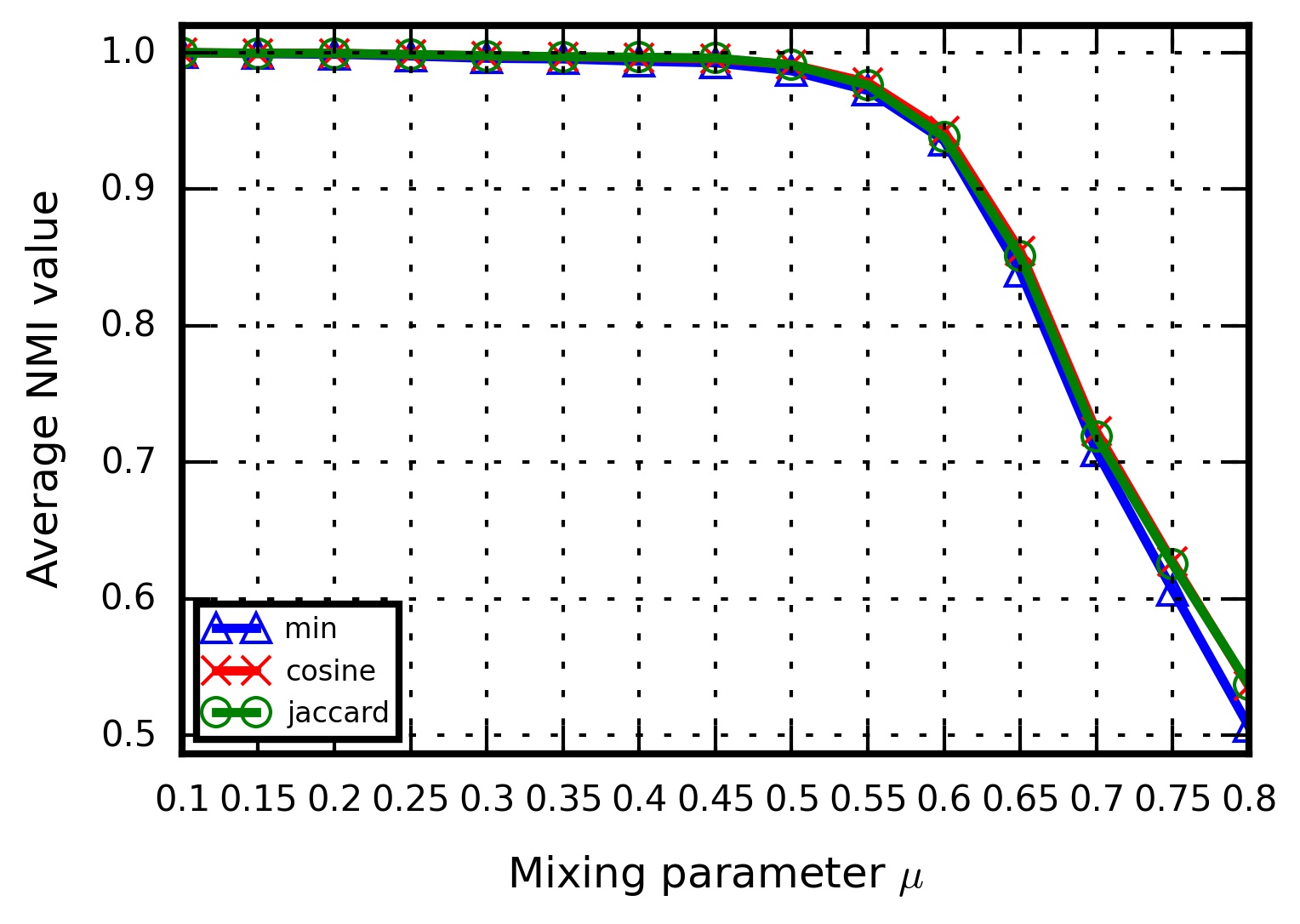 | 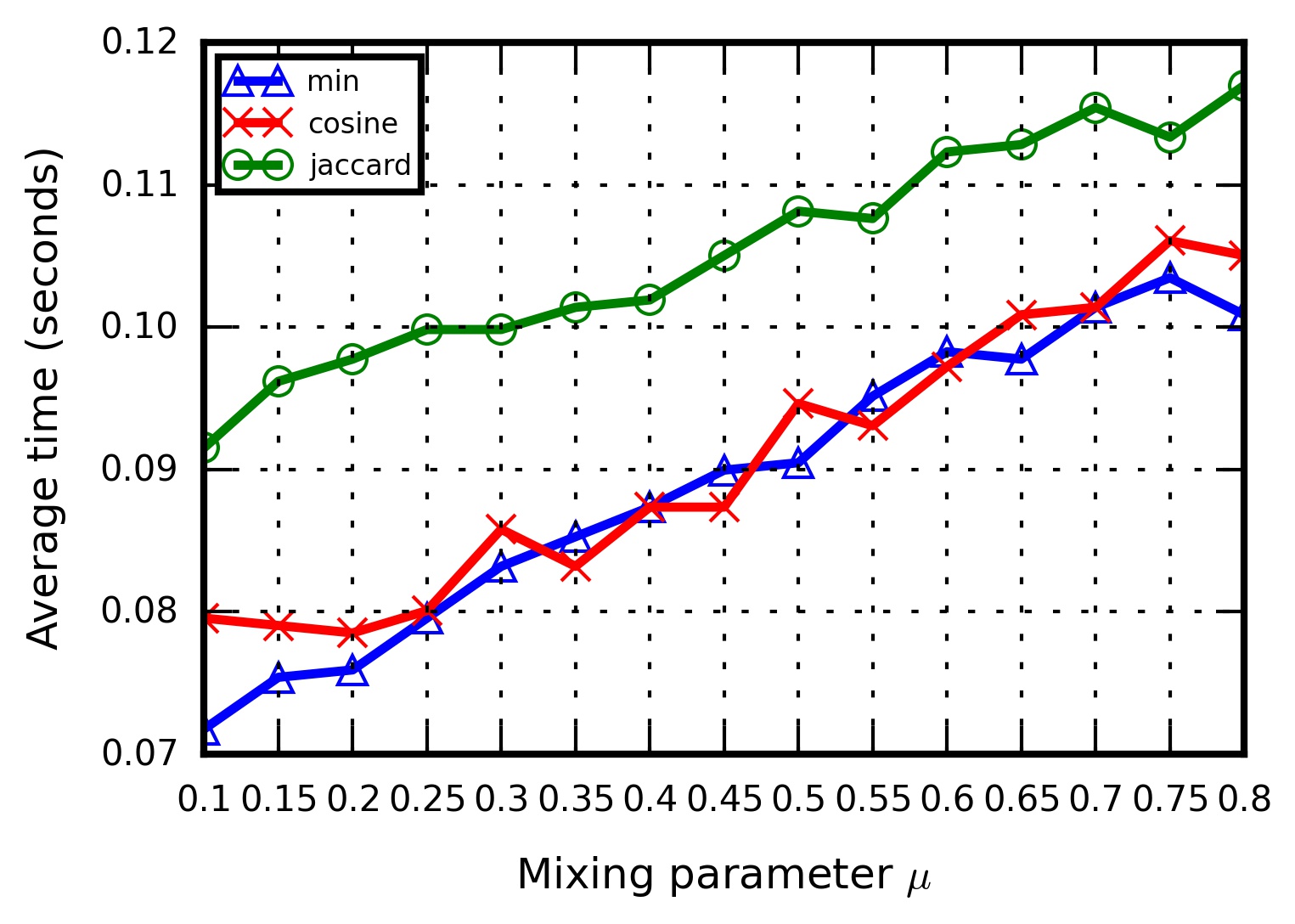 |
| --- | --- |
| (a) NMI value | (b) Execution time |
| **Fig S4-1. Similarity comparison for LFR-benchmark-1000S network.** (a) NMI value, (b) Execution time. | |

| 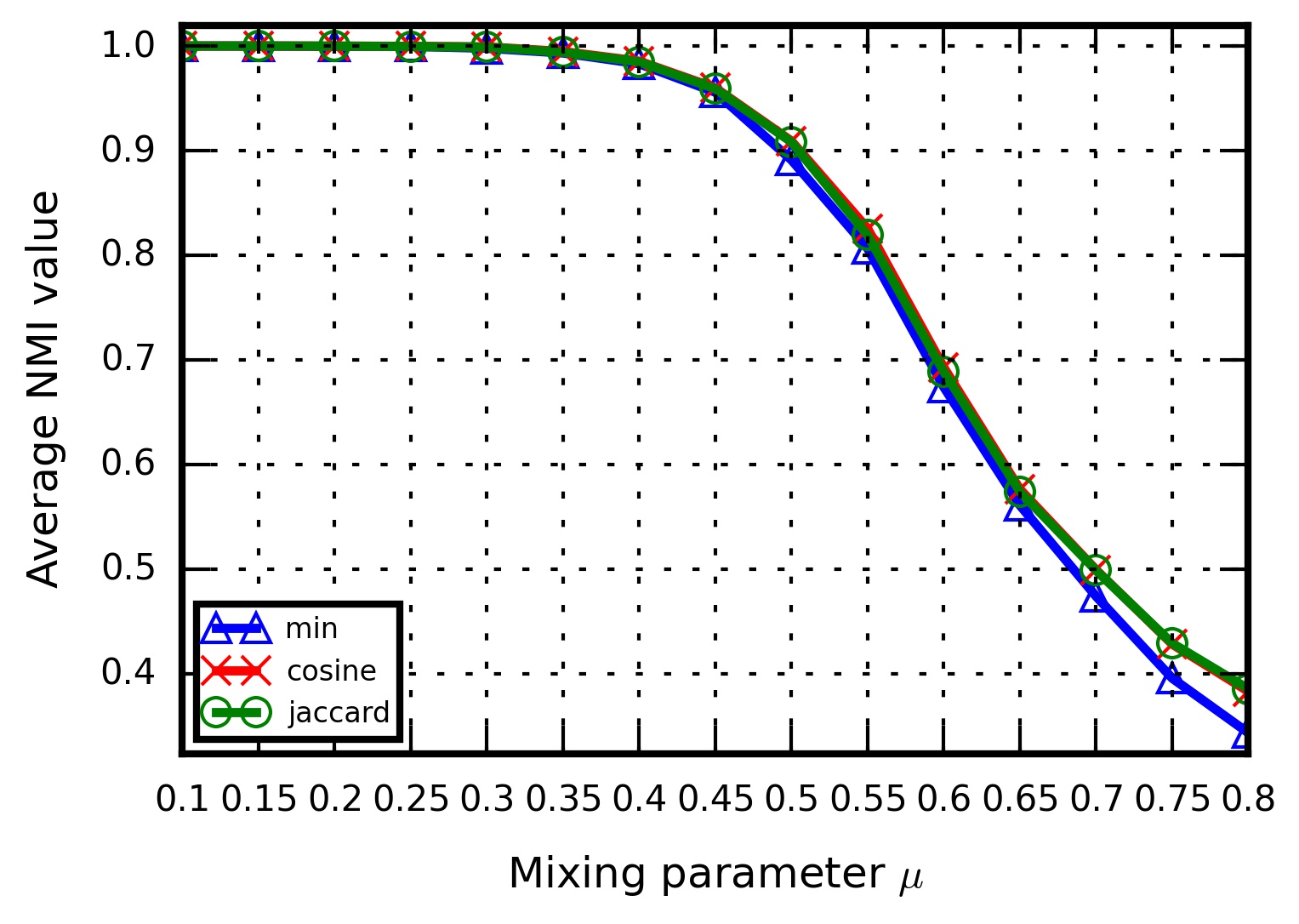 | 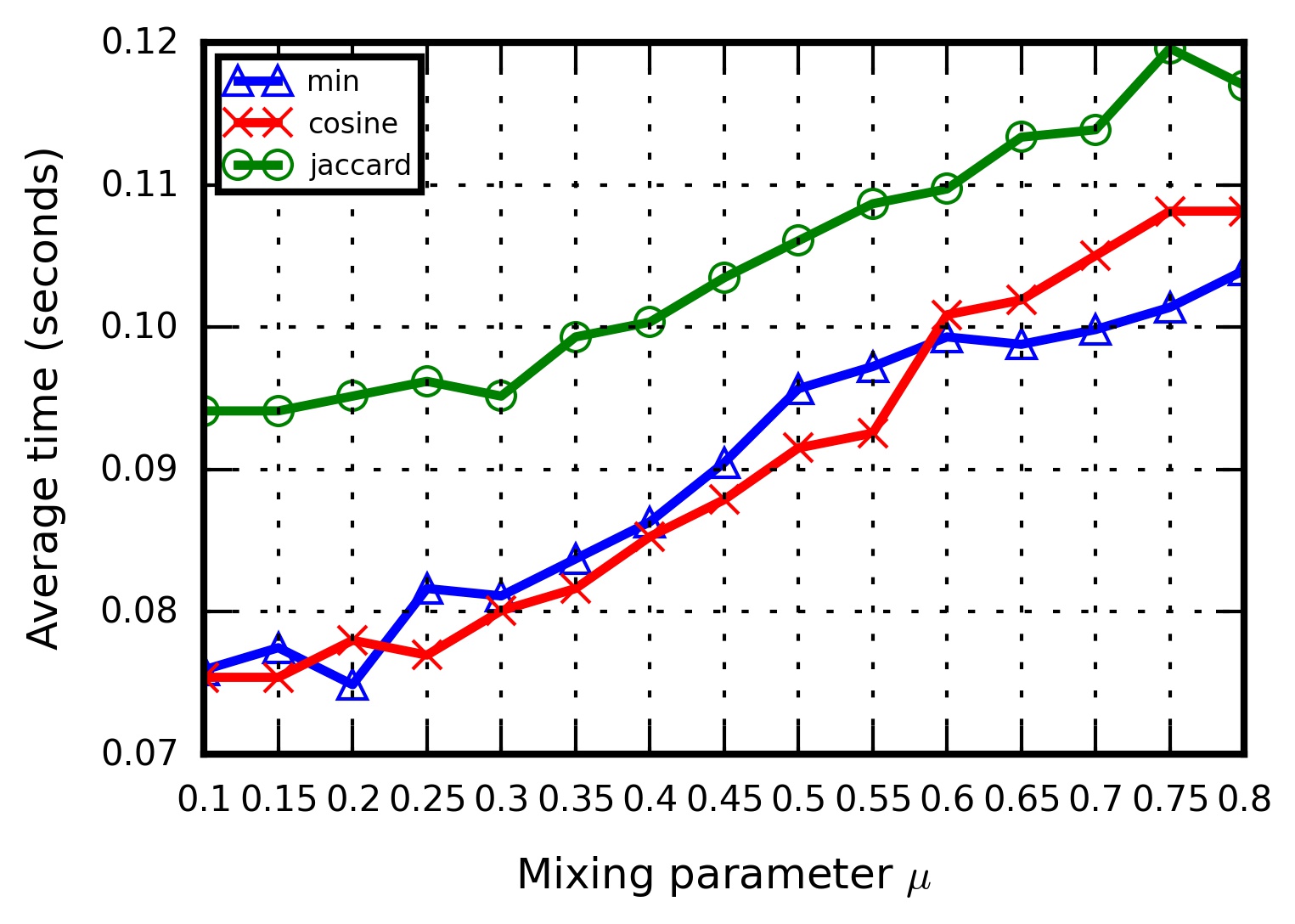 |
| --- | --- |
| (a) NMI value | (b) Execution time |
| **Fig S4-2. Similarity comparison for LFR-benchmark-1000B network.** (a) NMI value, (b) Execution time. | |

| 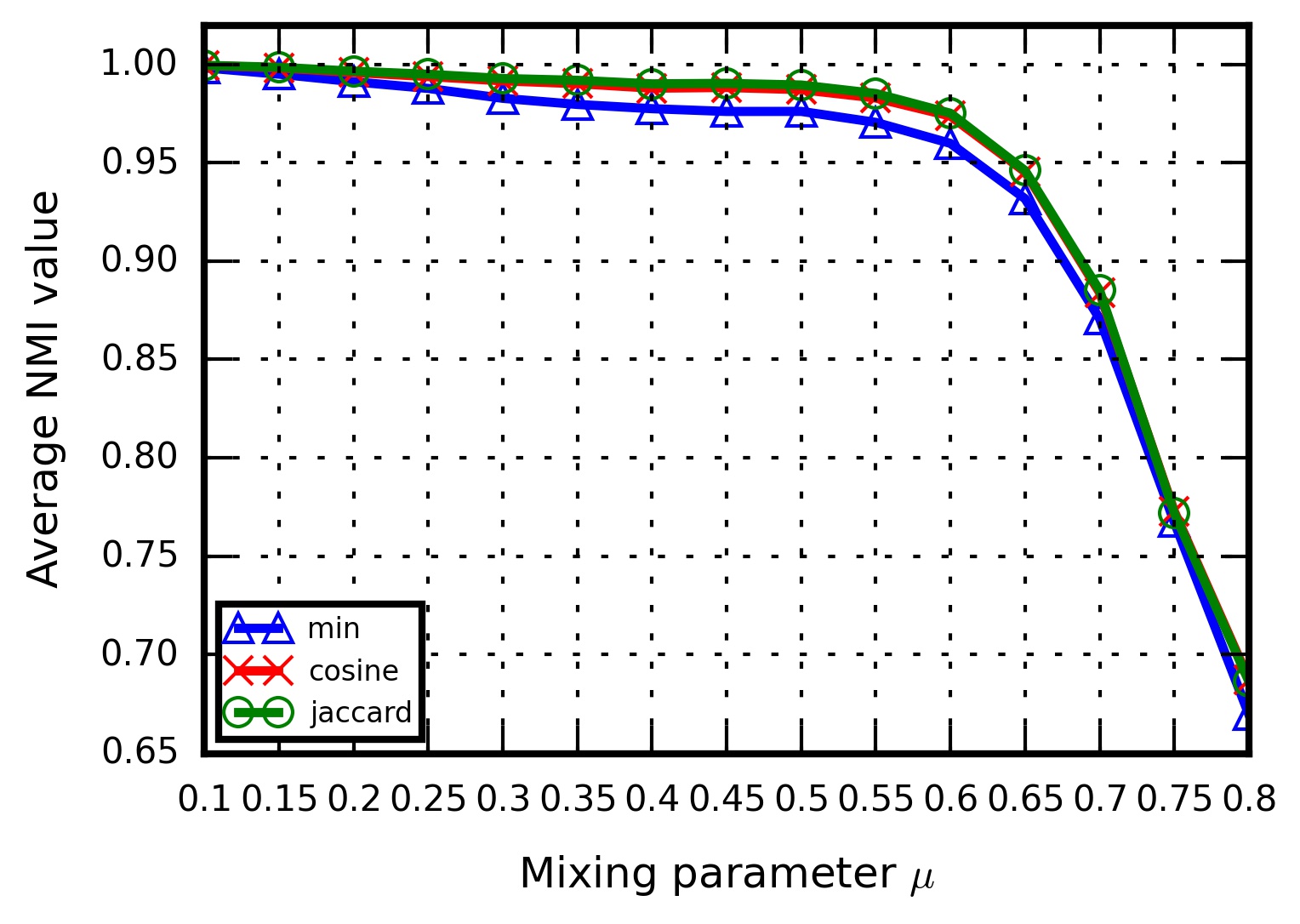 | 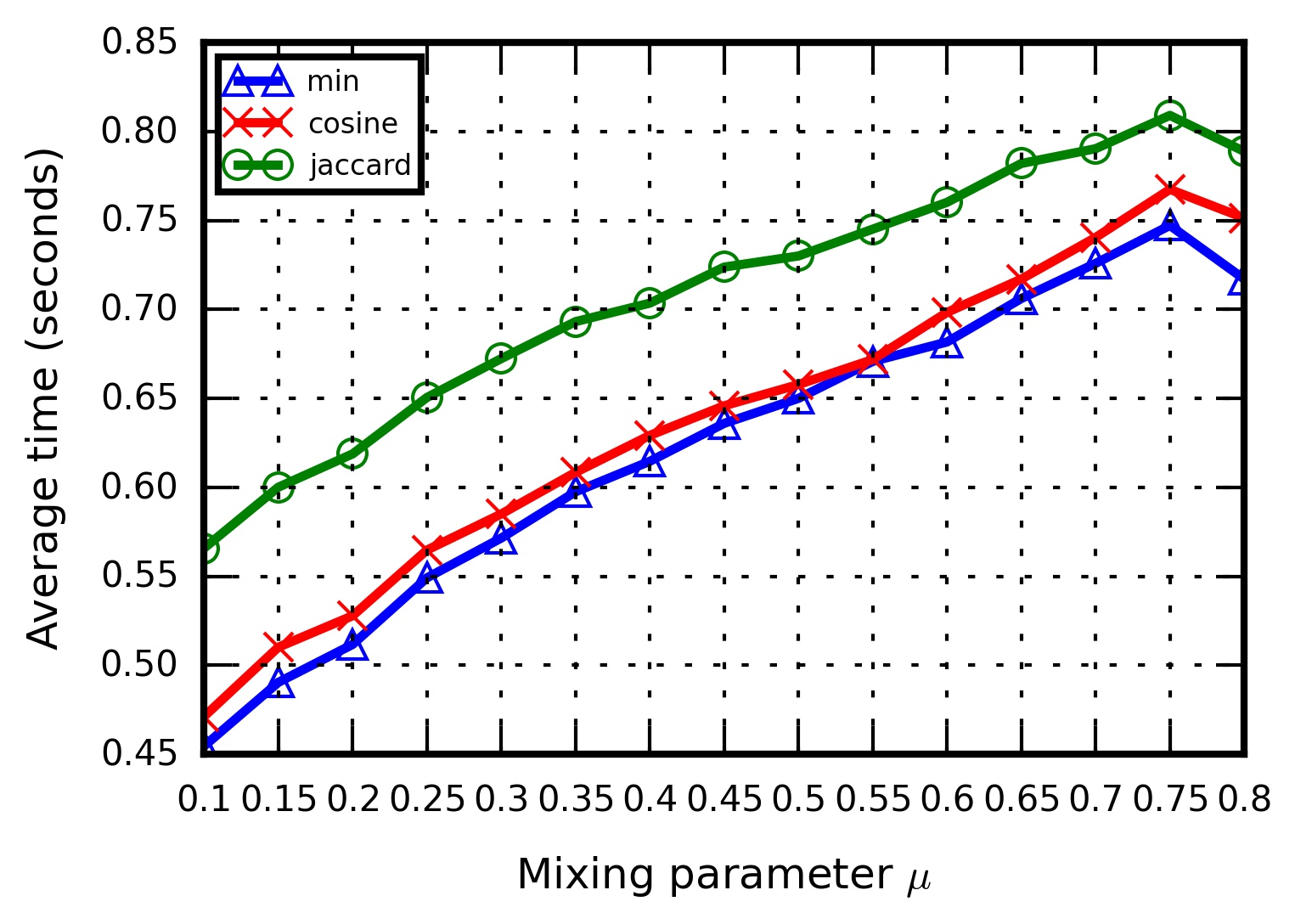 |
| --- | --- |
| (a) NMI value | (b) Execution time |
| **Fig S4-3. Similarity comparison for LFR-benchmark-5000S network.** (a) NMI value, (b) Execution time. | |

| 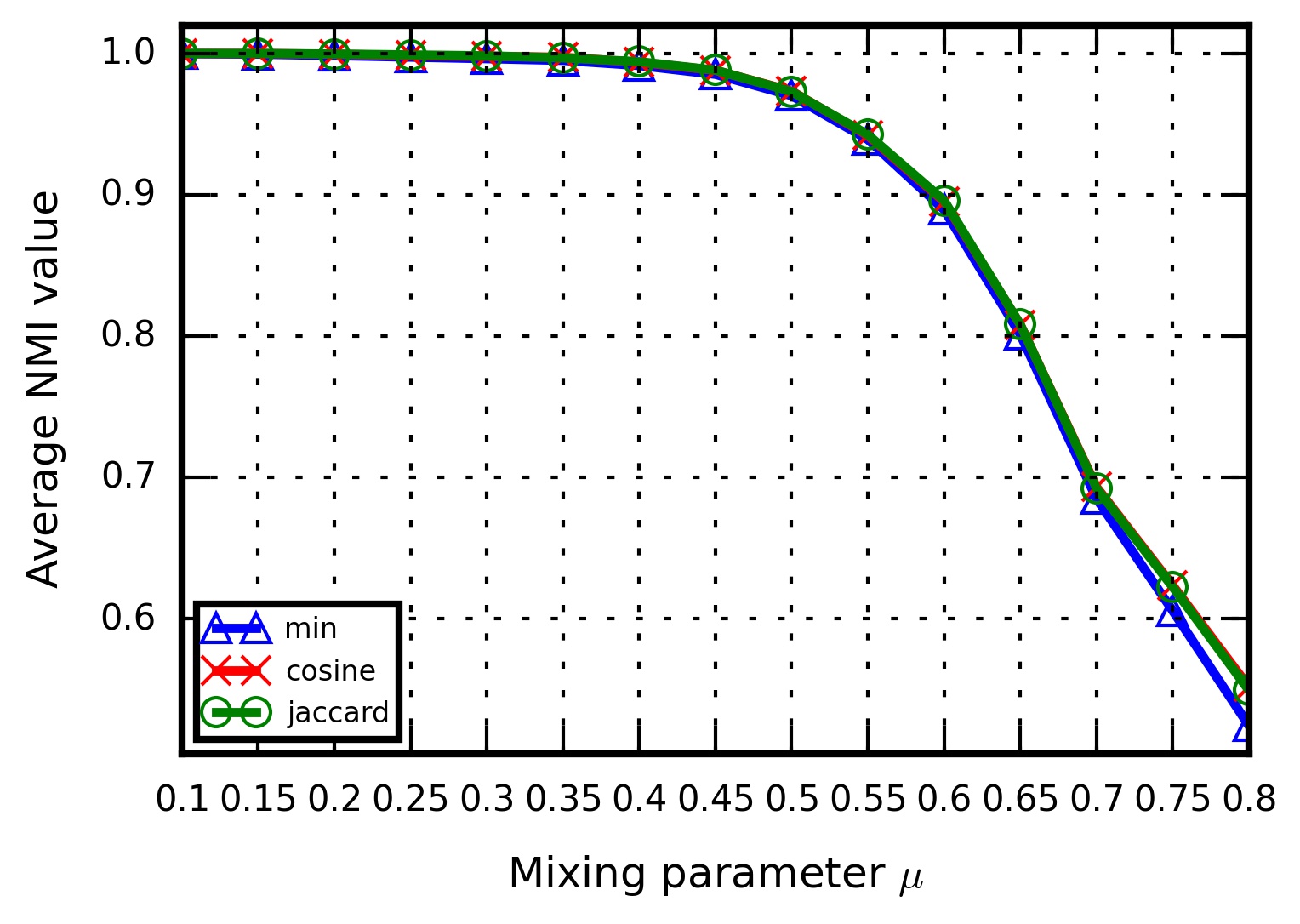 | 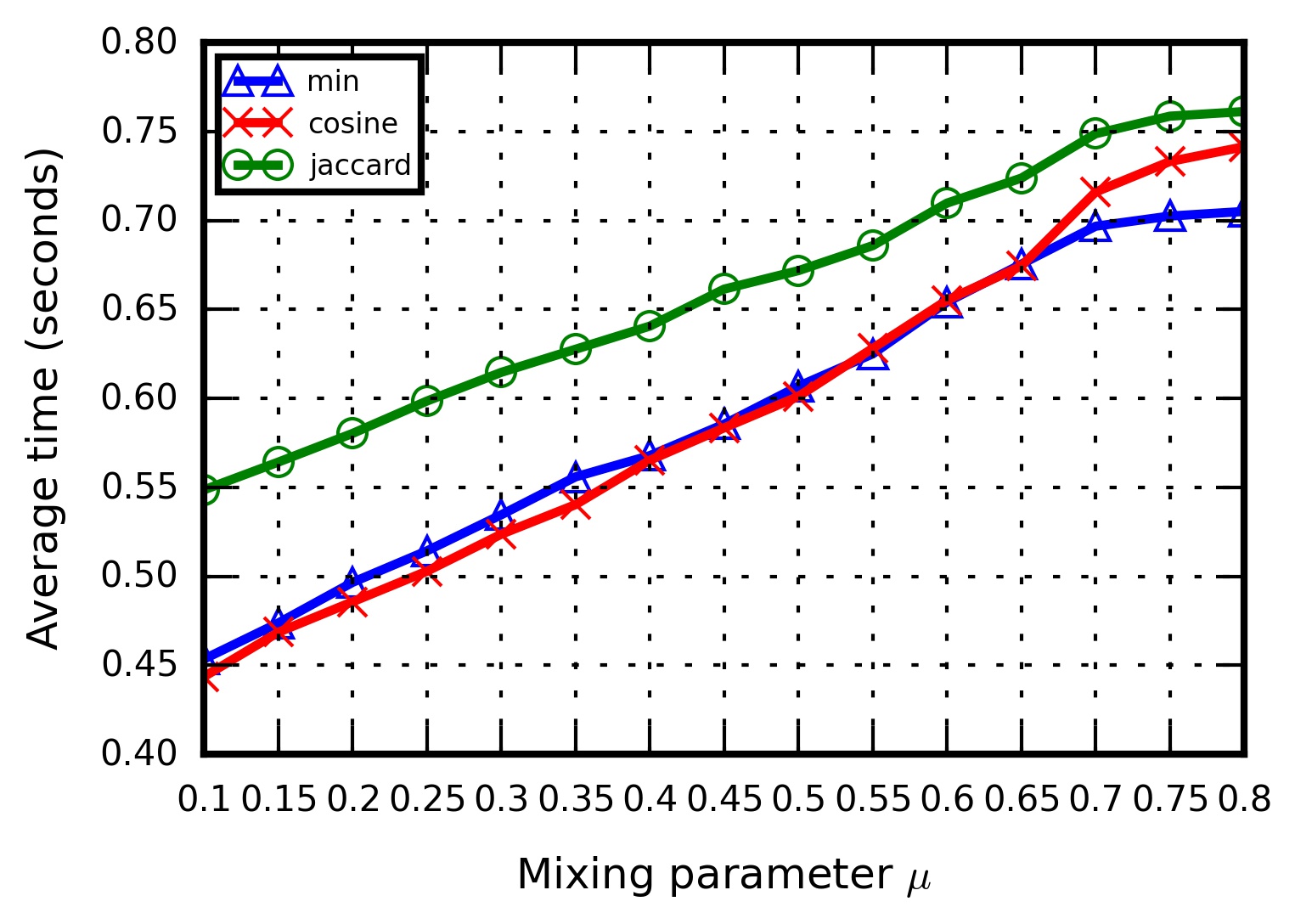 |
| --- | --- |
| (a) NMI value | (b) Execution time |
| **Fig S4-3. Similarity comparison for LFR-benchmark-5000B network.** (a) NMI value, (b) Execution time. | |

| 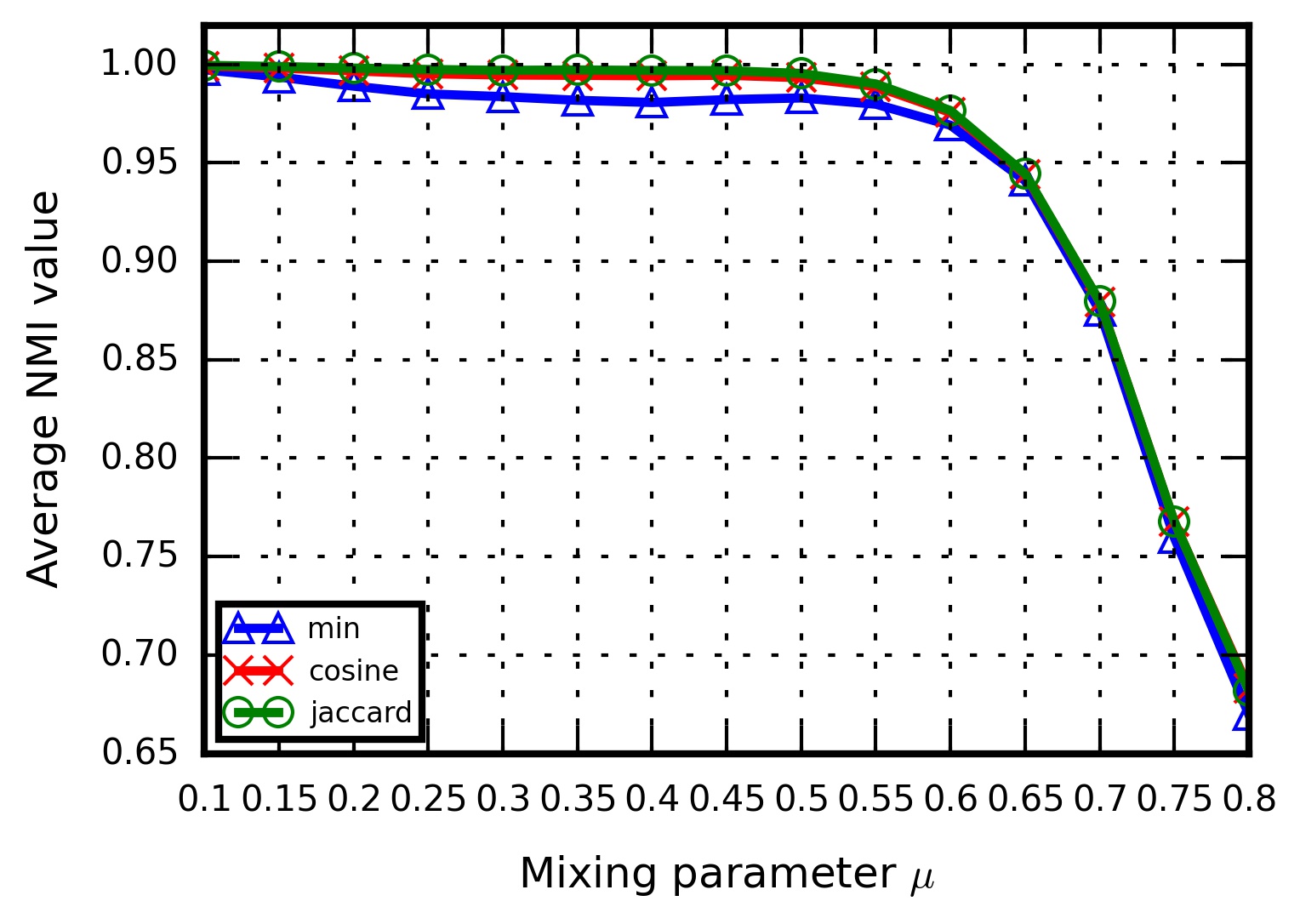 | 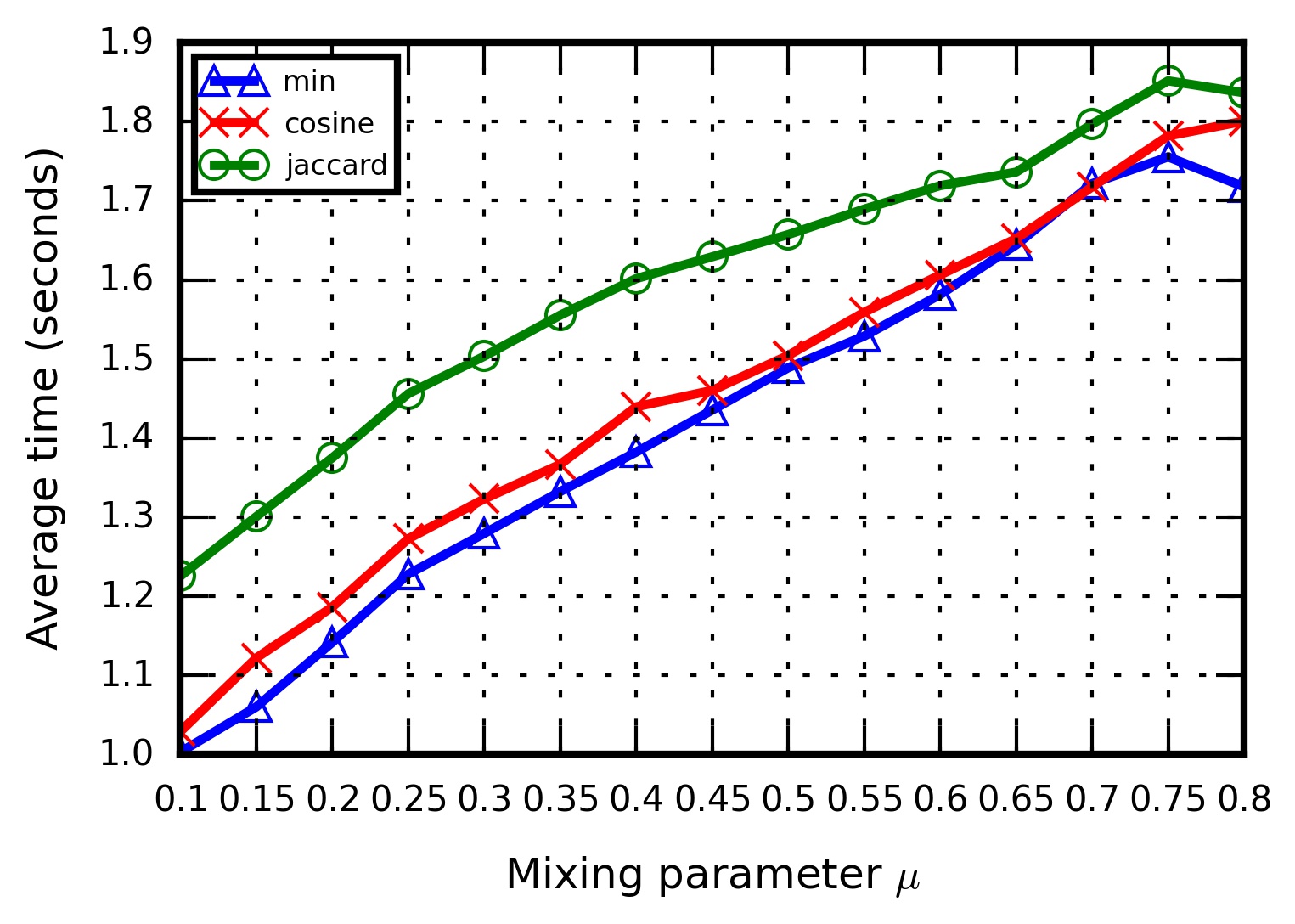 |
| --- | --- |
| (a) NMI value | (b) Execution time |
| **Fig S4-5. Similarity comparison for LFR-benchmark-10000S network.** (a) NMI value, (b) Execution time. | |

| 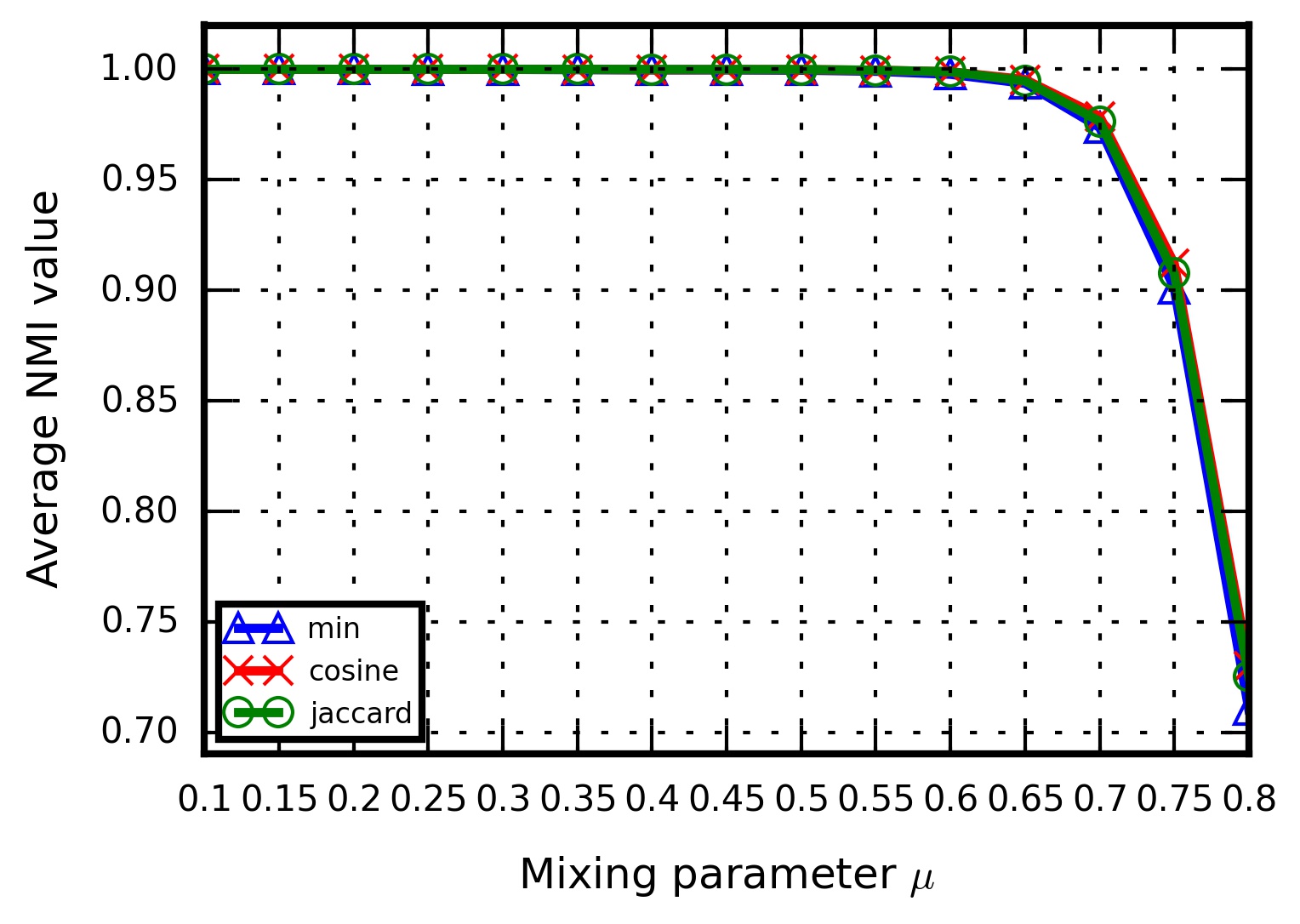 | 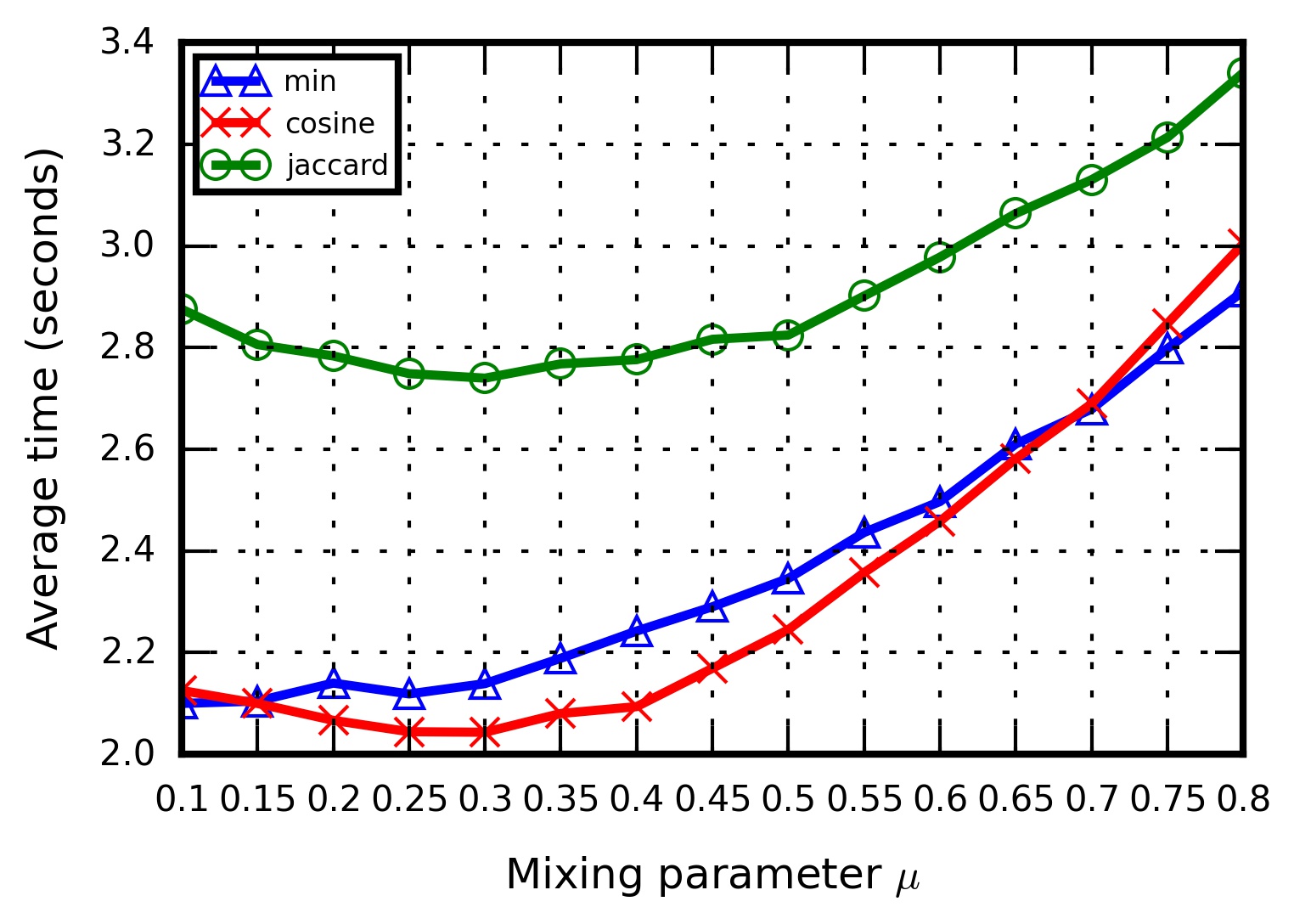 |
| --- | --- |
| (a) NMI value | (b) Execution time |
| **Fig S4-6. Similarity comparison for LFR-benchmark-10000B network.** (a) NMI value, (b) Execution time. | |

| 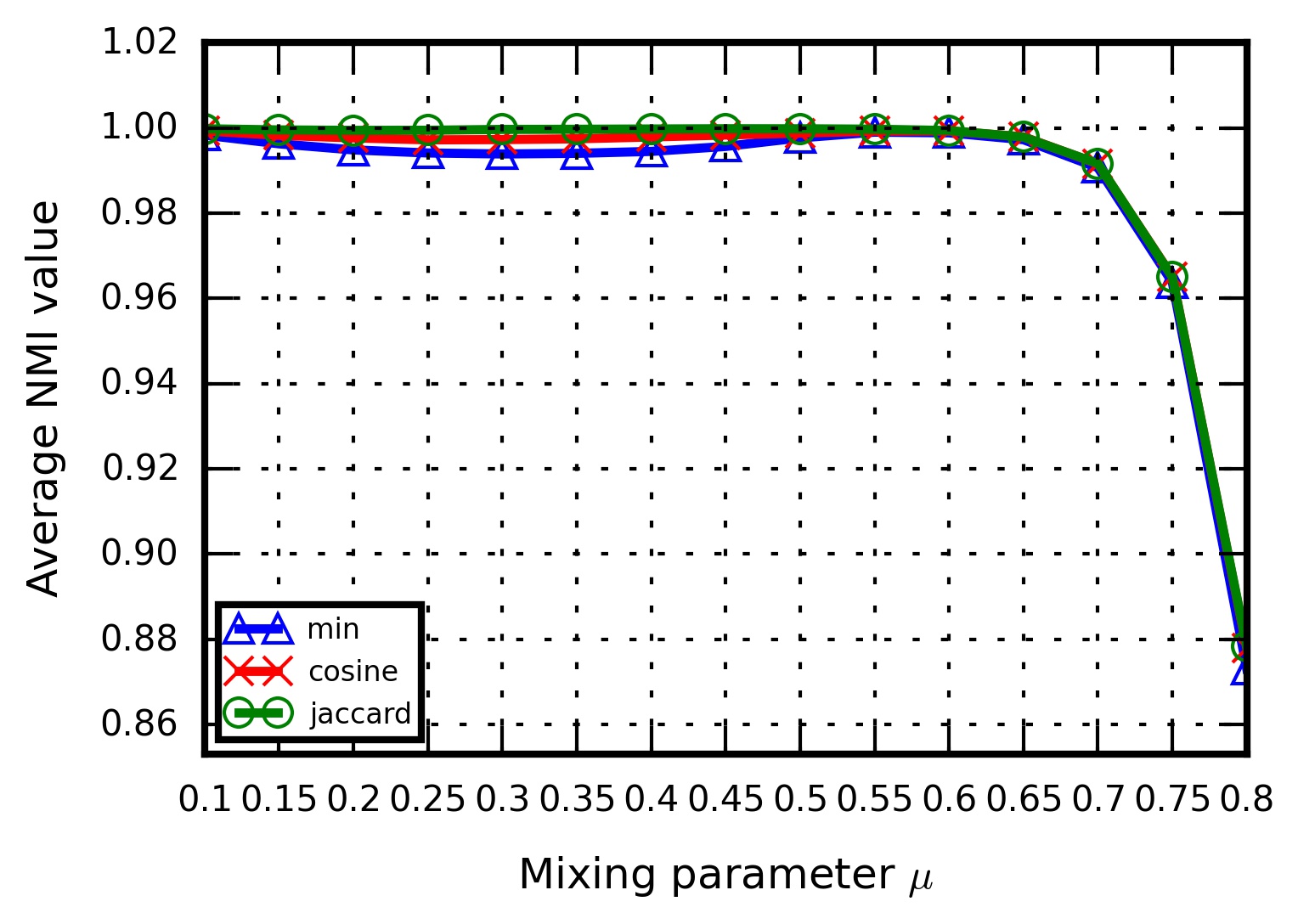 | 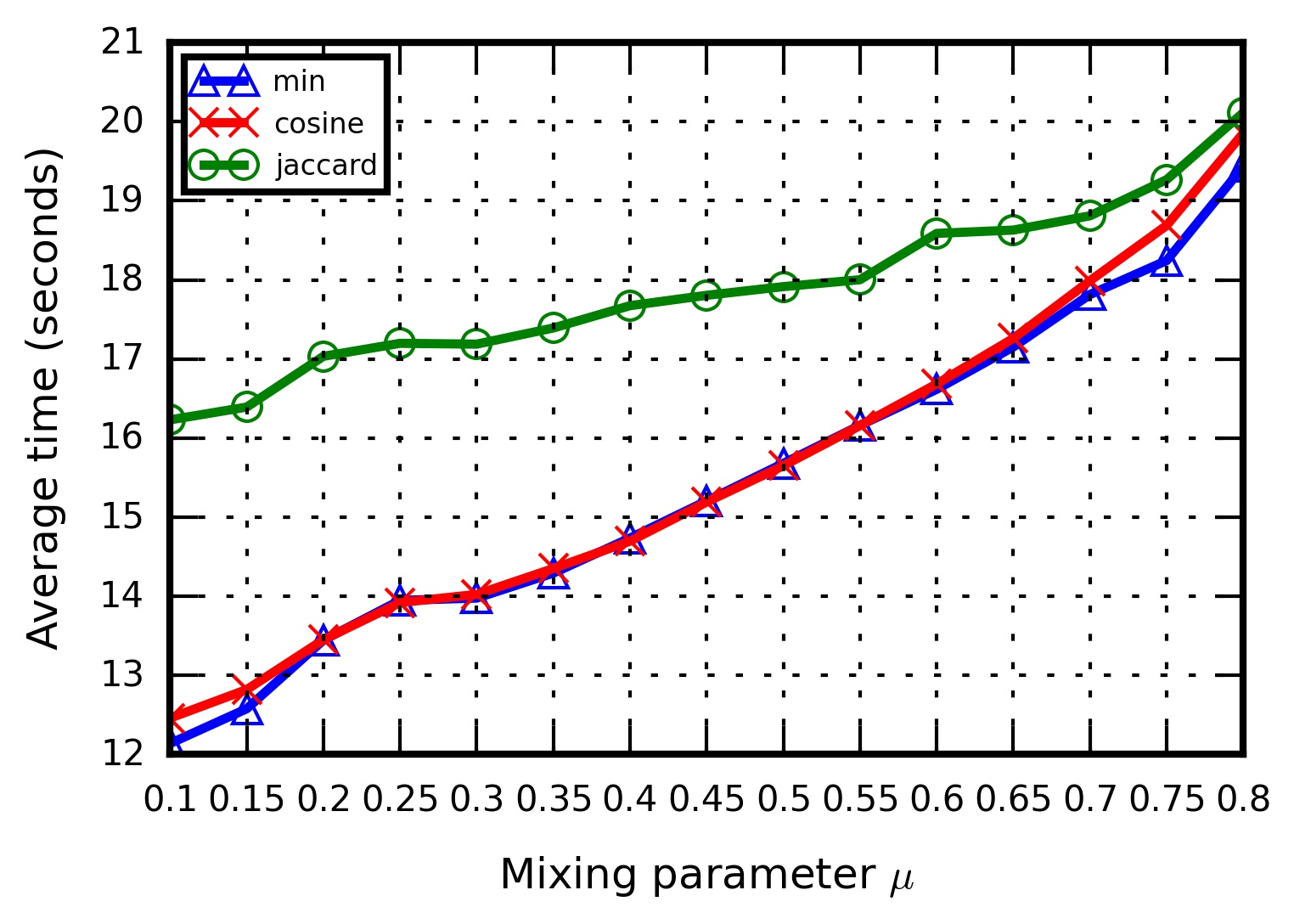 |
| --- | --- |
| (a) NMI value | (b) Execution time |
| **Fig S4-7. Similarity comparison for LFR-benchmark-50000S network.** (a) NMI value, (b) Execution time. | |

| 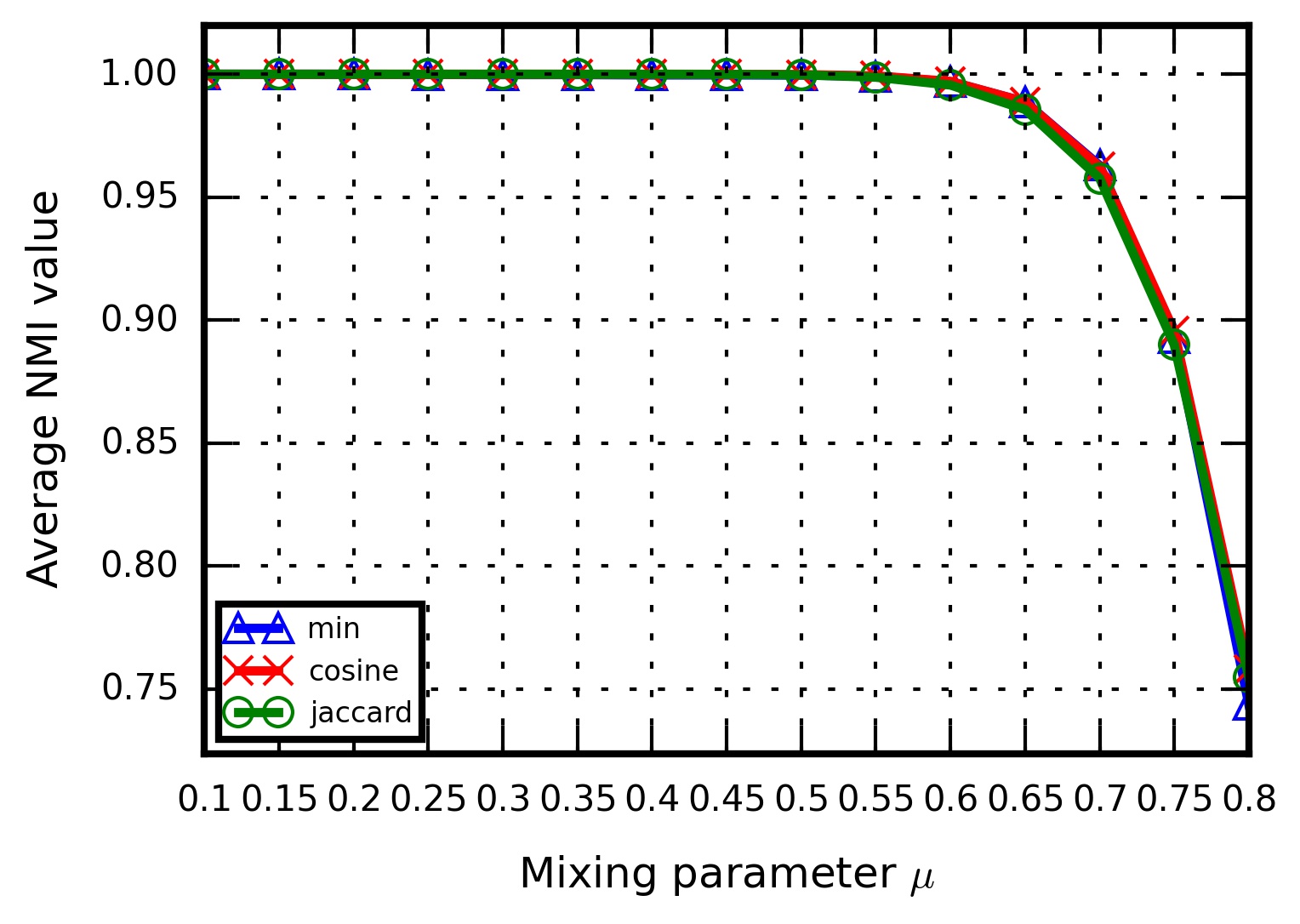 | 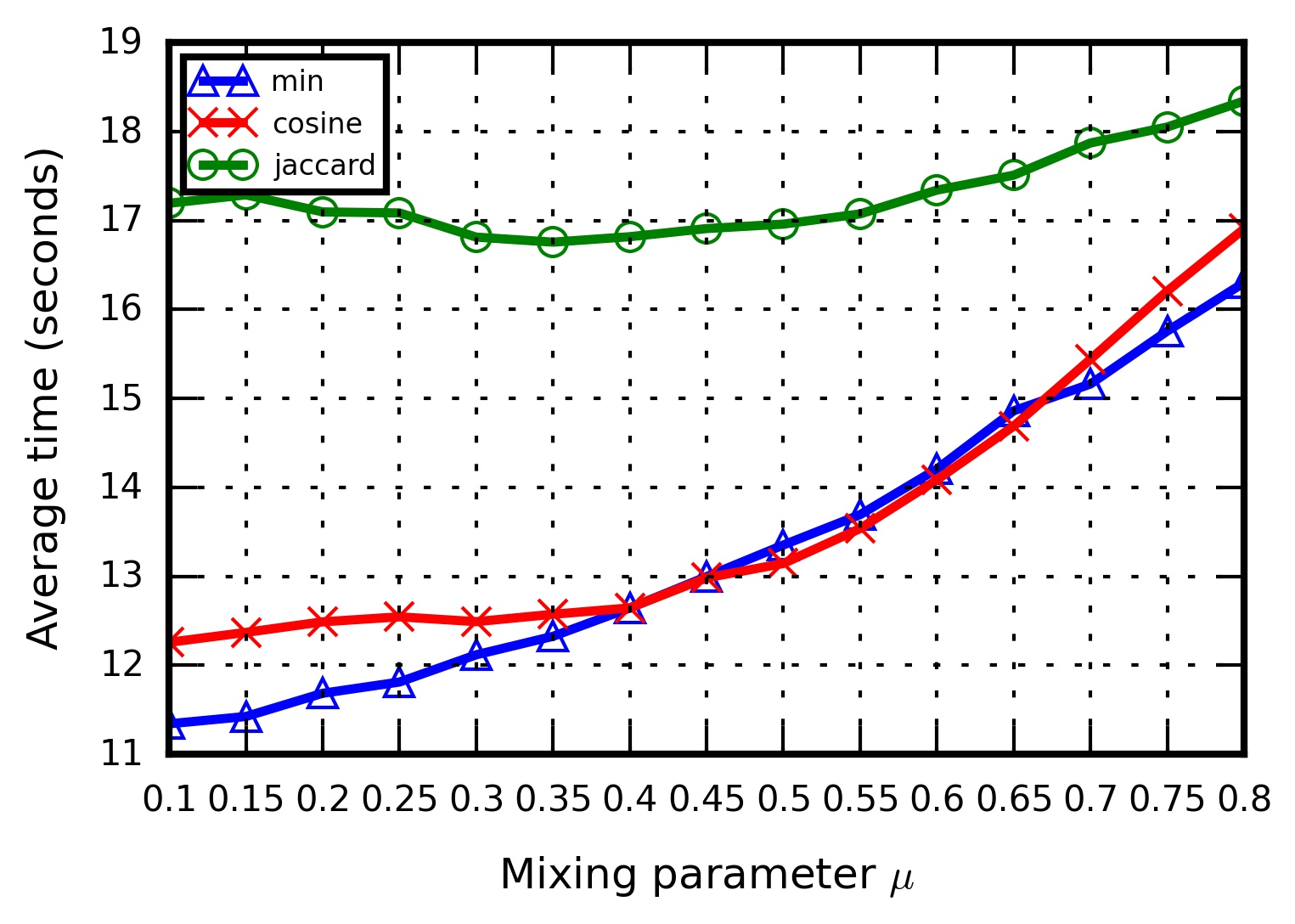 |
| --- | --- |
| (a) NMI value | (b) Execution time |
| **Fig S4-8. Similarity comparison for LFR-benchmark-50000B network.** (a) NMI value, (b) Execution time. | |
