# Supplementary material for: A community detection algorithm using network topologies and rule-based hierarchical arc-merging strategies
Source: PLoS One. 2017 Nov 9;12(11):e0187603. doi: 10.1371/journal.pone.0187603 (PMC5679540; doi:10.1371/journal.pone.0187603)
Supplement: S5 File — (DOCX) [file pone.0187603.s005.docx]

**S5 File. NMI Results of LFR benchmark networks.**

Table A. Avg. NMI for LFR benchmark network 1000S.

| Mixing  parameter $\mu$ | 0.1 | 0.15 | 0.2 | 0.25 | 0.3 | 0.35 | 0.4 | 0.45 | 0.5 | 0.55 | 0.6 | 0.65 | 0.7 | 0.75 | 0.8 |
| --- | --- | --- | --- | --- | --- | --- | --- | --- | --- | --- | --- | --- | --- | --- | --- |
| Louvain | 0.9953 | 0.9915 | 0.9885 | 0.9835 | 0.9811 | 0.9719 | 0.9673 | 0.9629 | 0.9545 | 0.9329 | 0.9198 | **0.8704** | ***0.6728*** | 0.3711 | 0.1883 |
| CNM | 0.9543 | 0.9049 | 0.8585 | 0.8219 | 0.7788 | 0.7351 | 0.6930 | 0.6333 | 0.5966 | 0.5461 | 0.4774 | 0.3639 | 0.2511 | 0.1645 | 0.1062 |
| DS | **1.0000** | **1.0000** | **1.0000** | **1.0000** | **1.0000** | ***0.9996*** | ***0.9985*** | 0.9922 | 0.9672 | 0.9237 | 0.8479 | 0.7477 | 0.6540 | ***0.5585*** | ***0.4615*** |
| INFOMAP | **1.0000** | **1.0000** | **1.0000** | **1.0000** | **1.0000** | **1.0000** | **0.9999** | **1.0000** | **0.9999** | **0.9992** | **0.9984** | 0.7640 | 0.0330 | 0.0000 | 0.0000 |
| HAM_Cosine_ | ***0.9999*** | ***0.9993*** | ***0.9994*** | ***0.9983*** | ***0.9974*** | 0.9968 | 0.9962 | ***0.9952*** | ***0.9911*** | ***0.9781*** | ***0.9423*** | ***0.8548*** | **0.7226** | **0.6271** | **0.5360** |
| **Bold**, best result.  **Bold** and *italics*, second best result. | | | | | | | | | | | | | | | |

Table B. Avg. NMI for LFR benchmark network 1000B.

| Mixing  parameter $\mu$ | 0.1 | 0.15 | 0.2 | 0.25 | 0.3 | 0.35 | 0.4 | 0.45 | 0.5 | 0.55 | 0.6 | 0.65 | 0.7 | 0.75 | 0.8 |
| --- | --- | --- | --- | --- | --- | --- | --- | --- | --- | --- | --- | --- | --- | --- | --- |
| Louvain | ***0.9998*** | **1.0000** | **1.0000** | **1.0000** | ***0.9998*** | ***0.9993*** | ***0.9986*** | ***0.9964*** | ***0.9903*** | **0.9732** | **0.8823** | **0.5845** | 0.2965 | 0.1442 | 0.0786 |
| CNM | 0.9756 | 0.9344 | 0.8890 | 0.8440 | 0.8027 | 0.7478 | 0.7062 | 0.6484 | 0.5846 | 0.4935 | 0.3692 | 0.2347 | 0.1319 | 0.0749 | 0.0504 |
| DS | **1.0000** | ***0.9999*** | 0.9992 | 0.9958 | 0.9835 | 0.9560 | 0.9065 | 0.8353 | 0.7581 | 0.6883 | 0.6107 | 0.5301 | ***0.4383*** | ***0.3593*** | ***0.3172*** |
| INFOMAP | **1.0000** | **1.0000** | **1.0000** | **1.0000** | **1.0000** | **1.0000** | **1.0000** | **1.0000** | **1.0000** | ***0.9660*** | 0.2989 | 0.0000 | 0.0000 | 0.0000 | 0.0000 |
| HAM_Cosine_ | **1.0000** | **1.0000** | ***0.9999*** | ***0.9997*** | 0.9992 | 0.9948 | 0.9855 | 0.9600 | 0.9092 | 0.8255 | ***0.6928*** | ***0.5763*** | **0.4994** | **0.4282** | **0.3830** |
| **Bold**, best result.  **Bold** and *italics*, second best result. | | | | | | | | | | | | | | | |

Table C. Avg. NMI for LFR benchmark network 5000S.

| Mixing  parameter $\mu$ | 0.1 | 0.15 | 0.2 | 0.25 | 0.3 | 0.35 | 0.4 | 0.45 | 0.5 | 0.55 | 0.6 | 0.65 | 0.7 | 0.75 | 0.8 |
| --- | --- | --- | --- | --- | --- | --- | --- | --- | --- | --- | --- | --- | --- | --- | --- |
| Louvain | 0.9570 | 0.9485 | 0.9400 | 0.9349 | 0.9257 | 0.9168 | 0.9079 | 0.8978 | 0.8891 | 0.8753 | 0.8630 | 0.8490 | 0.8266 | 0.6701 | 0.2836 |
| CNM | 0.9089 | 0.8449 | 0.7791 | 0.7275 | 0.6942 | 0.6521 | 0.6085 | 0.5635 | 0.5275 | 0.4843 | 0.4255 | 0.3414 | 0.2457 | 0.1646 | 0.1041 |
| DS | 0.9988 | ***0.9983*** | ***0.9982*** | ***0.9974*** | ***0.9965*** | ***0.9952*** | ***0.9927*** | ***0.9887*** | 0.9801 | 0.9613 | 0.9173 | 0.8447 | 0.7599 | 0.6640 | 0.5795 |
| INFOMAP | **1.0000** | **1.0000** | **1.0000** | **1.0000** | **0.9999** | **1.0000** | **0.9999** | **0.9998** | **0.9997** | **0.9994** | **0.9996** | **0.9999** | **0.9995** | **0.9874** | ***0.6306*** |
| HAM_Cosine_ | **0.9994** | 0.9982 | 0.9959 | 0.9938 | 0.9917 | 0.9902 | 0.9879 | 0.9883 | ***0.9872*** | ***0.9829*** | ***0.9737*** | ***0.9454*** | ***0.8840*** | ***0.7729*** | **0.6872** |
| **Bold**, best result.  **Bold** and *italics*, second best result. | | | | | | | | | | | | | | | |

Table D. Avg. NMI for LFR benchmark network 5000B.

| Mixing  parameter $\mu$ | 0.1 | 0.15 | 0.2 | 0.25 | 0.3 | 0.35 | 0.4 | 0.45 | 0.5 | 0.55 | 0.6 | 0.65 | 0.7 | 0.75 | 0.8 |
| --- | --- | --- | --- | --- | --- | --- | --- | --- | --- | --- | --- | --- | --- | --- | --- |
| Louvain | 0.9952 | 0.9918 | 0.9867 | 0.9837 | 0.9785 | 0.9722 | 0.9655 | 0.9532 | 0.9435 | 0.9320 | 0.9147 | 0.8859 | 0.7519 | 0.3183 | 0.1034 |
| CNM | 0.9132 | 0.8261 | 0.7681 | 0.7227 | 0.6839 | 0.6425 | 0.5908 | 0.5512 | 0.5087 | 0.4488 | 0.3743 | 0.2720 | 0.1660 | 0.0946 | 0.0578 |
| DS | **1.0000** | ***0.9999*** | ***0.9998*** | ***0.9995*** | ***0.9974*** | 0.9921 | 0.9772 | 0.9484 | 0.8978 | 0.8338 | 0.7620 | 0.6739 | 0.5844 | 0.5136 | ***0.4649*** |
| INFOMAP | **1.0000** | **1.0000** | **1.0000** | **1.0000** | **1.0000** | **1.0000** | **1.0000** | **1.0000** | **1.0000** | **1.0000** | **1.0000** | **0.9995** | **0.9970** | **0.9150** | 0.4265 |
| HAM_Cosine_ | ***0.9999*** | ***0.9999*** | 0.9994 | 0.9984 | 0.9979 | ***0.9973*** | ***0.9937*** | ***0.9880*** | ***0.9733*** | ***0.9418*** | ***0.8952*** | ***0.8080*** | ***0.6931*** | ***0.6235*** | **0.5517** |
| **Bold**, best result.  **Bold** and *italics*, second best result. | | | | | | | | | | | | | | | |

Table E. Avg. NMI for LFR benchmark network 10000S.

| Mixing  parameter $\mu$ | 0.1 | 0.15 | 0.2 | 0.25 | 0.3 | 0.35 | 0.4 | 0.45 | 0.5 | 0.55 | 0.6 | 0.65 | 0.7 | 0.75 | 0.8 |
| --- | --- | --- | --- | --- | --- | --- | --- | --- | --- | --- | --- | --- | --- | --- | --- |
| Louvain | 0.9566 | 0.9469 | 0.9369 | 0.9280 | 0.9190 | 0.9100 | 0.9000 | 0.8916 | 0.8815 | 0.8692 | 0.8545 | 0.8411 | 0.8212 | 0.7145 | 0.2363 |
| CNM | 0.9070 | 0.8271 | 0.7452 | 0.6876 | 0.6519 | 0.6086 | 0.5618 | 0.5185 | 0.4853 | 0.4356 | 0.3821 | 0.3028 | 0.2094 | 0.1337 | 0.0813 |
| DS | ***0.9999*** | ***0.9998*** | ***0.9996*** | ***0.9990*** | ***0.9983*** | ***0.9963*** | 0.9933 | 0.9872 | 0.9761 | 0.9535 | 0.9102 | 0.8308 | 0.7316 | 0.6398 | 0.5791 |
| INFOMAP | **1.0000** | **1.0000** | **1.0000** | **1.0000** | **1.0000** | **1.0000** | **1.0000** | **1.0000** | **1.0000** | **1.0000** | **1.0000** | **1.0000** | **0.9996** | **0.9931** | **0.7121** |
| HAM_Cosine_ | 0.9992 | 0.9981 | 0.9967 | 0.9953 | 0.9946 | 0.9945 | ***0.9942*** | ***0.9945*** | ***0.9933*** | ***0.9888*** | ***0.9756*** | ***0.9440*** | ***0.8793*** | ***0.7676*** | ***0.6827*** |
| **Bold**, best result.  **Bold** and *italics*, second best result. | | | | | | | | | | | | | | | |

Table F. Avg. NMI for LFR benchmark network 10000B.

| Mixing  parameter $\mu$ | 0.1 | 0.15 | 0.2 | 0.25 | 0.3 | 0.35 | 0.4 | 0.45 | 0.5 | 0.55 | 0.6 | 0.65 | 0.7 | 0.75 | 0.8 |
| --- | --- | --- | --- | --- | --- | --- | --- | --- | --- | --- | --- | --- | --- | --- | --- |
| Louvain | **1.0000** | **1.0000** | ***0.9999*** | ***0.9998*** | ***0.9994*** | ***0.9980*** | 0.9944 | 0.9893 | 0.9781 | 0.9646 | 0.9478 | 0.9310 | 0.9153 | 0.8858 | ***0.7513*** |
| CNM | 0.8385 | 0.7834 | 0.7466 | 0.7012 | 0.6644 | 0.6308 | 0.5939 | 0.5593 | 0.5189 | 0.4786 | 0.4376 | 0.3854 | 0.3169 | 0.2129 | 0.0995 |
| DS | **1.0000** | **1.0000** | **1.0000** | **1.0000** | **1.0000** | **1.0000** | **1.0000** | **1.0000** | ***0.9999*** | 0.9990 | 0.9941 | 0.9685 | 0.8853 | 0.7608 | 0.6203 |
| INFOMAP | **1.0000** | **1.0000** | **1.0000** | **1.0000** | **1.0000** | **1.0000** | **1.0000** | **1.0000** | **1.0000** | **1.0000** | **1.0000** | **1.0000** | **1.0000** | **1.0000** | **0.9333** |
| HAM_Cosine_ | **1.0000** | **1.0000** | **1.0000** | **1.0000** | **1.0000** | **1.0000** | ***0.9998*** | ***0.9998*** | 0.9998 | ***0.9996*** | ***0.9989*** | ***0.9953*** | ***0.9787*** | ***0.9120*** | 0.7301 |
| **Bold**, best result.  **Bold** and *italics*, second best result. | | | | | | | | | | | | | | | |

Table G. Avg. NMI for LFR benchmark network 50000S.

| Mixing  parameter $\mu$ | 0.1 | 0.15 | 0.2 | 0.25 | 0.3 | 0.35 | 0.4 | 0.45 | 0.5 | 0.55 | 0.6 | 0.65 | 0.7 | 0.75 | 0.8 |
| --- | --- | --- | --- | --- | --- | --- | --- | --- | --- | --- | --- | --- | --- | --- | --- |
| Louvain | 0.9783 | 0.9693 | 0.9606 | 0.9513 | 0.9433 | 0.9352 | 0.9278 | 0.9194 | 0.9106 | 0.9015 | 0.8892 | 0.8773 | 0.8613 | 0.8441 | 0.8226 |
| CNM | - | - | - | - | - | - | - | - | - | - | - | - | - | - | - |
| DS | * | * | * | * | * | * | * | * | * | * | * | * | * | * | * |
| INFOMAP | **1.0000** | **1.0000** | **1.0000** | **1.0000** | **1.0000** | **1.0000** | **1.0000** | **1.0000** | **1.0000** | **1.0000** | **1.0000** | **1.0000** | **1.0000** | **1.0000** | **1.0000** |
| HAM_Cosine_ | ***0.9993*** | ***0.9983*** | ***0.9977*** | ***0.9972*** | ***0.9973*** | ***0.9974*** | ***0.9979*** | ***0.9983*** | ***0.9988*** | ***0.9992*** | ***0.9993*** | ***0.9979*** | ***0.9916*** | ***0.9651*** | ***0.8779*** |
| **Bold**, best result.  **Bold** and *italics*, second best result.  -, runs exceeded at least 1 hours for 30 networks and would cost many hours for all sets of networks.  *, memory allocation error (e.g., “std::bad_alloc”). | | | | | | | | | | | | | | | |

Table H. Avg. NMI for LFR benchmark network 50000B.

| Mixing  parameter $\mu$ | 0.1 | 0.15 | 0.2 | 0.25 | 0.3 | 0.35 | 0.4 | 0.45 | 0.5 | 0.55 | 0.6 | 0.65 | 0.7 | 0.75 | 0.8 |
| --- | --- | --- | --- | --- | --- | --- | --- | --- | --- | --- | --- | --- | --- | --- | --- |
| Louvain | **1.0000** | **1.0000** | ***0.9996*** | ***0.9983*** | ***0.9958*** | ***0.9904*** | ***0.9831*** | ***0.9723*** | 0.9604 | 0.9477 | 0.9355 | 0.9239 | 0.9095 | 0.8792 | 0.7128 |
| CNM | - | - | - | - | - | - | - | - | - | - | - | - | - | - | - |
| DS | * | * | * | * | * | * | * | * | * | * | * | * | * | * | * |
| INFOMAP | **1.0000** | **1.0000** | **1.0000** | **1.0000** | **1.0000** | **1.0000** | **1.0000** | **1.0000** | **1.0000** | **1.0000** | **1.0000** | **1.0000** | **1.0000** | **0.9999** | **0.9974** |
| HAM_Cosine_ | **1.0000** | **1.0000** | **1.0000** | **1.0000** | **1.0000** | **1.0000** | **1.0000** | **1.0000** | ***0.9999*** | ***0.9993*** | ***0.9971*** | ***0.9888*** | ***0.9624*** | ***0.8954*** | ***0.7577*** |
| **Bold**, best result.  **Bold** and *italics*, second best result.  -, runs exceeded at least 1 hours for 30 networks and would cost many hours for all sets of networks.  *, memory allocation error (e.g., “std::bad_alloc”). | | | | | | | | | | | | | | | |

Table I. Summary of each method for LFR benchmark networks 1000S/B.

| Method | 1000S | | | 1000B | | |
| --- | --- | --- | --- | --- | --- | --- |
|  | # of 1^st^ place | # of 2^st^ place | **Final place** | # of 1^st^ place | # of 2^st^ place | **Final place** |
| Louvain | 1 | 1 | 4 | 6 | 6 | **2** |
| CNM | - | - | - | - | - | - |
| DS | 5 | 2 | **2** | 1 | 1 | 4 |
| INFOMAP | 11 | - | **1** | 9 | 1 | **1** |
| HAM_Cosine_ | 3 | 10 | **2** | 5 | 3 | **2** |
| The final place is determined by # of 1^st^ place and then # of 2^nd^ place.  In 1000S, DS was placed in the 2^nd^ place in the first half of $\mu$ range (e.g., $\mu\leq0.5$) and HAM was placed in the 2^nd^ place in the second half of $\mu$ range (e.g., $\mu\geq0.5$).  In 1000B, Louvain was placed in the 2^nd^ place in the first half of $\mu$ range and HAM was placed in the 2^nd^ place in the second half of $\mu$ range. | | | | | | |

Table J. Summary of each method for LFR benchmark networks 5000S/B.

| Method | 5000S | | | 5000B | | |
| --- | --- | --- | --- | --- | --- | --- |
|  | # of 1^st^ place | # of 2^st^ place | **Final place** | # of 1^st^ place | # of 2^st^ place | **Final place** |
| Louvain | - | - | - | - | - | - |
| CNM | - | - | - | - | - | - |
| DS | - | 7 | 3 | 1 | 4 | 3 |
| INFOMAP | 14 | 1 | **1** | 14 | - | **1** |
| HAM_Cosine_ | 1 | 7 | **2** | 1 | 11 | **2** |
| The final place is determined by # of 1^st^ place and then # of 2^nd^ place. | | | | | | |

Table K. Summary of each method for LFR benchmark networks 10000S/B.

| Method | 10000S | | | 10000B | | |
| --- | --- | --- | --- | --- | --- | --- |
|  | # of 1^st^ place | # of 2^st^ place | **Final place** | # of 1^st^ place | # of 2^st^ place | **Final place** |
| Louvain | - | - | - | 2 | 5 | 4 |
| CNM | - | - | - | - | - | - |
| DS | - | 6 | 3 | 8 | 1 | **2** |
| INFOMAP | 15 | - | **1** | 15 | - | **1** |
| HAM_Cosine_ | - | 9 | **2** | 6 | 8 | **2** |
| The final place is determined by # of 1^st^ place and then # of 2^nd^ place.  In 10000B, DS was placed in the 2^nd^ place in the first half of $\mu$ range and HAM was placed in the 2^nd^ place in the second half of $\mu$ range. | | | | | | |

Table L. Summary of each method for LFR benchmark networks 50000S/B.

| Method | 50000S | | | 50000B | | |
| --- | --- | --- | --- | --- | --- | --- |
|  | # of 1^st^ place | # of 2^st^ place | **Final place** | # of 1^st^ place | # of 2^st^ place | **Final place** |
| Louvain | - | - | - | 2 | 6 | 3 |
| CNM | - | - |  | - | - | - |
| DS | - | - | - | - | - | - |
| INFOMAP | 15 | - | **1** | 15 | - | **1** |
| HAM_Cosine_ | - | 15 | **2** | 8 | 7 | **2** |
| The final place is determined by # of 1^st^ place and then # of 2^nd^ place. | | | | | | |

Table M. Summary of final place for all sets of LFR benchmark networks

| Method | # of 1^st^ place | # of 2^st^ place | **Final place** |
| --- | --- | --- | --- |
| Louvain | - | 1 | 4 |
| CNM | - | - | - |
| DS | - | 2 | 3 |
| INFOMAP | 8 | - | **1** |
| HAM_Cosine_ | - | 8 | **2** |
