# Supplementary material for: A community detection algorithm using network topologies and rule-based hierarchical arc-merging strategies
Source: PLoS One. 2017 Nov 9;12(11):e0187603. doi: 10.1371/journal.pone.0187603 (PMC5679540; doi:10.1371/journal.pone.0187603)
Supplement: S6 File — (DOCX) [file pone.0187603.s006.docx]

**S6 File. Execution time results of LFR benchmark networks.**

Table S6-1. Avg. execution time for LFR benchmark network 1000S.

| Mixing  parameter$\mu$ | 0.1 | | 0.15 | | 0.2 | | 0.25 | | | 0.3 | | 0.35 | | 0.4 | | 0.45 | |
| --- | --- | --- | --- | --- | --- | --- | --- | --- | --- | --- | --- | --- | --- | --- | --- | --- | --- |
|  | Avg. | Std. | Avg. | Std. | Avg. | Std. | Avg. | Std. | | Avg. | Std. | Avg. | Std. | Avg. | Std. | Avg. | Std. |
| Louvain | **0.0406** | 0.0086 | **0.0411** | 0.0085 | **0.0395** | 0.0088 | **0.0432** | 0.0066 | | **0.0432** | 0.0066 | **0.0458** | 0.0056 | **0.0468** | 0.0057 | **0.0473** | 0.0049 |
| CNM | 0.4103 | 0.0372 | 0.4846 | 0.0554 | 0.5928 | 0.0293 | 0.6666 | 0.0353 | | 0.7514 | 0.0503 | 0.8242 | 0.0441 | 0.8928 | 0.0373 | 0.9828 | 0.0450 |
| DS | 0.5351 | 0.0217 | 0.5668 | 0.0207 | 0.6084 | 0.0245 | 0.6375 | 0.0257 | | 0.6620 | 0.0223 | 0.6833 | 0.0268 | 0.7082 | 0.0226 | 0.7275 | 0.0211 |
| INFOMAP | 0.2153 | 0.0195 | 0.2148 | 0.0165 | 0.2106 | 0.0119 | 0.2301 | 0.0149 | | 0.2322 | 0.0158 | 0.2304 | 0.0138 | 0.2366 | 0.0121 | 0.2501 | 0.0124 |
| HAM_Cosine_ | ***0.0796*** | 0.0116 | ***0.0790*** | 0.0133 | ***0.0785*** | 0.0103 | ***0.0801*** | 0.0112 | | ***0.0858*** | 0.0119 | ***0.0832*** | 0.0109 | ***0.0874*** | 0.0118 | ***0.0874*** | 0.0076 |
|  |  |  |  |  |  |  |  |  | |  |  |  |  |  |  |  |  |
| Mixing  parameter$\mu$ | 0.5 | | 0.55 | | 0.6 | | 0.65 | | 0.7 | | | 0.75 | | 0.8 | | ~ | |
|  | Avg. | Std. | Avg. | Std. | Avg. | Std. | Avg. | Std. | | Avg. | Std. | Avg. | Std. | Avg. | Std. | ~ | ~ |
| Louvain | **0.0504** | 0.0066 | **0.0520** | 0.0074 | **0.0536** | 0.0077 | **0.0655** | 0.0094 | | **0.0733** | 0.0190 | **0.0671** | 0.0082 | **0.0697** | 0.0078 | ~ | ~ |
| CNM | 1.0218 | 0.0496 | 1.0852 | 0.0593 | 1.1383 | 0.0531 | 1.1653 | 0.0616 | | 1.1726 | 0.0449 | 1.1736 | 0.0558 | 1.0592 | 0.2164 | ~ | ~ |
| DS | 0.7160 | 0.0286 | 0.7072 | 0.0300 | 0.6822 | 0.0332 | 0.6230 | 0.0325 | | 0.5741 | 0.0402 | 0.5314 | 0.0304 | 0.5018 | 0.0264 | ~ | ~ |
| INFOMAP | 0.2626 | 0.0176 | 0.2798 | 0.0193 | 0.3172 | 0.0318 | 0.3526 | 0.0822 | | 0.2408 | 0.0634 | 0.2434 | 0.0359 | 0.2496 | 0.0282 | ~ | ~ |
| HAM_Cosine_ | ***0.0946*** | 0.0161 | ***0.0931*** | 0.0124 | ***0.0972*** | 0.0112 | ***0.1009*** | 0.0088 | | ***0.1014*** | 0.0097 | ***0.1061*** | 0.0130 | ***0.1050*** | 0.0113 | ~ | ~ |
| **Bold**, best result.  **Bold** and *italics*, second best result. | | | | | | | | | | | | | | | | | |

Table S6-2. Avg. execution time for LFR benchmark network 1000B.

| Mixing  parameter $\mu$ | 0.1 | | 0.15 | | 0.2 | | 0.25 | | 0.3 | | 0.35 | | 0.4 | | 0.45 | |
| --- | --- | --- | --- | --- | --- | --- | --- | --- | --- | --- | --- | --- | --- | --- | --- | --- |
|  | Avg. | Std. | Avg. | Std. | Avg. | Std. | Avg. | Std. | Avg. | Std. | Avg. | Std. | Avg. | Std. | Avg. | Std. |
| Louvain | **0.0390** | 0.0078 | **0.0426** | 0.0069 | **0.0437** | 0.0084 | **0.0426** | 0.0069 | **0.0432** | 0.0077 | **0.0468** | 0.0057 | **0.0463** | 0.0063 | **0.0494** | 0.0071 |
| CNM | 0.4404 | 0.0377 | 0.5652 | 0.0360 | 0.6438 | 0.0356 | 0.7348 | 0.0415 | 0.8143 | 0.0352 | 0.8944 | 0.0469 | 0.9823 | 0.0341 | 1.0494 | 0.0517 |
| DS | 0.6048 | 0.0169 | 0.6438 | 0.0235 | 0.6734 | 0.0255 | 0.6932 | 0.0244 | 0.7129 | 0.0296 | 0.7171 | 0.0297 | 0.7072 | 0.0259 | 0.6932 | 0.0301 |
| INFOMAP | 0.2220 | 0.0096 | 0.2267 | 0.0132 | 0.2402 | 0.0331 | 0.2397 | 0.0124 | 0.2444 | 0.0136 | 0.2517 | 0.0112 | 0.2595 | 0.0110 | 0.2746 | 0.0159 |
| HAM_Cosine_ | ***0.0754*** | 0.0121 | 0.***0754*** | 0.0115 | ***0.0780*** | 0.0099 | ***0.0770*** | 0.0113 | ***0.0801*** | 0.0112 | ***0.0816*** | 0.0104 | ***0.0853*** | 0.0126 | ***0.0879*** | 0.0085 |
|  |  |  |  |  |  |  |  |  |  |  |  |  |  |  |  |  |
| Mixing  parameter $\mu$ | 0.5 | | 0.55 | | 0.6 | | 0.65 | | 0.7 | | 0.75 | | 0.8 | | ~ | |
|  | Avg. | Std. | Avg. | Std. | Avg. | Std. | Avg. | Std. | Avg. | Std. | Avg. | Std. | Avg. | Std. | ~ | ~ |
| Louvain | **0.0520** | 0.0084 | **0.0582** | 0.0098 | **0.0655** | 0.0110 | **0.0686** | 0.0095 | **0.0718** | 0.0095 | **0.0728** | 0.0093 | **0.0738** | 0.0120 | ~ | ~ |
| CNM | 1.0712 | 0.0529 | 1.1341 | 0.0358 | 1.1596 | 0.0484 | 1.1482 | 0.0528 | 1.1476 | 0.0618 | 1.1534 | 0.0452 | 1.1320 | 0.0677 | ~ | ~ |
| DS | 0.6588 | 0.0381 | 0.6178 | 0.0316 | 0.5736 | 0.0366 | 0.5288 | 0.0379 | 0.5034 | 0.0261 | 0.5018 | 0.0279 | 0.4820 | 0.0352 | ~ | ~ |
| INFOMAP | 0.2995 | 0.0211 | 0.3427 | 0.0414 | 0.2844 | 0.0808 | 0.2512 | 0.0246 | 0.2584 | 0.0368 | 0.2454 | 0.0282 | 0.2517 | 0.0329 | ~ | ~ |
| HAM_Cosine_ | ***0.0915*** | 0.0112 | ***0.0926*** | 0.0089 | ***0.1009*** | 0.0105 | ***0.1019*** | 0.0088 | ***0.1050*** | 0.0089 | ***0.1082*** | 0.0127 | ***0.1082*** | 0.0113 | ~ | ~ |
| **Bold**, best result.  **Bold** and *italics*, second best result. | | | | | | | | | | | | | | | | |

Table S6-3. Avg. execution time for LFR benchmark network 5000S.

| Mixing  parameter $\mu$ | 0.1 | | 0.15 | | 0.2 | | 0.25 | | 0.3 | | 0.35 | | 0.4 | | 0.45 | |
| --- | --- | --- | --- | --- | --- | --- | --- | --- | --- | --- | --- | --- | --- | --- | --- | --- |
|  | Avg. | Std. | Avg. | Std. | Avg. | Std. | Avg. | Std. | Avg. | Std. | Avg. | Std. | Avg. | Std. | Avg. | Std. |
| Louvain | **0.0957** | 0.0105 | **0.1035** | 0.0085 | **0.1134** | 0.0098 | **0.1196** | 0.0116 | **0.1295** | 0.0146 | **0.1414** | 0.0139 | **0.1633** | 0.0160 | **0.1799** | 0.0149 |
| CNM | 1.7815 | 0.0943 | 3.2770 | 0.5543 | 5.2842 | 0.2169 | 6.8318 | 0.2273 | 8.1801 | 0.2681 | 8.4001 | 1.5187 | 11.2128 | 0.2379 | 12.5034 | 0.2061 |
| DS | 3.4034 | 0.0616 | 4.1860 | 0.0947 | 5.0154 | 0.1148 | 5.8573 | 0.1342 | 6.6898 | 0.1143 | 7.5151 | 0.1516 | 8.2613 | 0.1340 | 8.8608 | 0.1714 |
| INFOMAP | 0.9599 | 0.0223 | 1.0010 | 0.0276 | 1.0499 | 0.0438 | 1.1003 | 0.0498 | 1.1579 | 0.0678 | 1.2085 | 0.0717 | 1.2844 | 0.0628 | 1.3728 | 0.0639 |
| HAM_Cosine_ | ***0.4711*** | 0.0158 | ***0.5101*** | 0.0128 | ***0.5278*** | 0.0166 | ***0.5647*** | 0.0302 | ***0.5850*** | 0.0155 | ***0.6084*** | 0.0221 | ***0.6292*** | 0.0163 | ***0.6458*** | 0.0204 |
|  |  |  |  |  |  |  |  |  |  |  |  |  |  |  |  |  |
| Mixing  parameter $\mu$ | 0.5 | | 0.55 | | 0.6 | | 0.65 | | 0.7 | | 0.75 | | 0.8 | | ~ | |
|  | Avg. | Std. | Avg. | Std. | Avg. | Std. | Avg. | Std. | Avg. | Std. | Avg. | Std. | Avg. | Std. | ~ | ~ |
| Louvain | **0.1867** | 0.0164 | **0.2106** | 0.0183 | **0.2257** | 0.0170 | **0.2486** | 0.0189 | **0.2964** | 0.0330 | **0.3921** | 0.0566 | **0.3656** | 0.0529 | ~ | ~ |
| CNM | 13.6791 | 0.2236 | 14.6822 | 0.2420 | 15.4185 | 0.2028 | 15.9713 | 0.2354 | 15.7092 | 1.2348 | 15.9079 | 0.3244 | 14.1409 | 0.5018 | ~ | ~ |
| DS | 9.1671 | 0.1868 | 9.0927 | 0.2130 | 8.6263 | 0.2408 | 7.5093 | 0.3105 | 5.7294 | 0.2403 | 3.9198 | 0.1842 | 2.5496 | 0.1490 | ~ | ~ |
| INFOMAP | 1.4700 | 0.0743 | 1.6058 | 0.0418 | 1.7722 | 0.0402 | 2.0467 | 0.1419 | 2.5938 | 0.1139 | 3.9577 | 0.3632 | 10.7661 | 1.6751 | ~ | ~ |
| HAM_Cosine_ | ***0.6578*** | 0.0140 | ***0.6718*** | 0.0150 | ***0.6984*** | 0.0183 | ***0.7171*** | 0.0142 | ***0.7405*** | 0.0170 | ***0.7675*** | 0.0158 | ***0.7514*** | 0.0162 | ~ | ~ |
| **Bold**, best result.  **Bold** and *italics*, second best result. | | | | | | | | | | | | | | | | |

Table S6-4. Avg. execution time for LFR benchmark network 5000B.

| Mixing  parameter $\mu$ | 0.1 | | 0.15 | | 0.2 | | 0.25 | | 0.3 | | 0.35 | | 0.4 | | 0.45 | |
| --- | --- | --- | --- | --- | --- | --- | --- | --- | --- | --- | --- | --- | --- | --- | --- | --- |
|  | Avg. | Std. | Avg. | Std. | Avg. | Std. | Avg. | Std. | Avg. | Std. | Avg. | Std. | Avg. | Std. | Avg. | Std. |
| Louvain | **0.0952** | 0.0084 | **0.1030** | 0.0125 | **0.1076** | 0.0084 | **0.1196** | 0.0116 | **0.1279** | 0.0136 | **0.1404** | 0.0166 | **0.1570** | 0.0166 | **0.1721** | 0.0153 |
| CNM | 2.9598 | 0.1800 | 4.7986 | 0.1983 | 6.4652 | 0.2178 | 7.8141 | 0.1854 | 9.2196 | 0.2289 | 10.6928 | 0.2763 | 12.1956 | 0.2696 | 13.2772 | 0.2957 |
| DS | 4.4621 | 0.0761 | 5.3248 | 0.1106 | 6.0996 | 0.0998 | 6.7127 | 0.1107 | 7.2972 | 0.1637 | 7.6503 | 0.1286 | 7.8083 | 0.1796 | 7.9123 | 0.1809 |
| INFOMAP | 0.9656 | 0.0186 | 1.0130 | 0.0201 | 1.0605 | 0.0311 | 1.0910 | 0.0235 | 1.1315 | 0.0278 | 1.2212 | 0.0567 | 1.2480 | 0.0285 | 1.3229 | 0.0333 |
| HAM_Cosine_ | ***0.4436*** | 0.0132 | ***0.4685*** | 0.0187 | ***0.4857*** | 0.0119 | ***0.5028*** | 0.0119 | ***0.5236*** | 0.0160 | ***0.5403*** | 0.0182 | ***0.5652*** | 0.0169 | ***0.5834*** | 0.0183 |
|  |  |  |  |  |  |  |  |  |  |  |  |  |  |  |  |  |
| Mixing  parameter $\mu$ | 0.5 | | 0.55 | | 0.6 | | 0.65 | | 0.7 | | 0.75 | | 0.8 | | ~ | |
|  | Avg. | Std. | Avg. | Std. | Avg. | Std. | Avg. | Std. | Avg. | Std. | Avg. | Std. | Avg. | Std. | ~ | ~ |
| Louvain | **0.1877** | 0.0233 | **0.1981** | 0.0202 | **0.2262** | 0.0223 | **0.2740** | 0.0319 | **0.3780** | 0.0793 | **0.3255** | 0.0493 | **0.3011** | 0.0318 | ~ | ~ |
| CNM | 14.3744 | 0.2243 | 15.3197 | 0.2024 | 15.7877 | 0.1749 | 16.0269 | 0.1768 | 15.6983 | 0.4369 | 13.9977 | 1.7813 | 11.4993 | 2.0559 | ~ | ~ |
| DS | 7.6440 | 0.2458 | 7.2187 | 0.2897 | 6.2593 | 0.3211 | 4.8027 | 0.2478 | 3.5204 | 0.2220 | 2.5288 | 0.1556 | 2.0270 | 0.1256 | ~ | ~ |
| INFOMAP | 1.4550 | 0.0362 | 1.6624 | 0.0431 | 1.8684 | 0.0572 | 2.3431 | 0.1751 | 3.1642 | 0.2447 | 14.2579 | 4.0823 | 8.6169 | 1.2455 | ~ | ~ |
| HAM_Cosine_ | ***0.6011*** | 0.0165 | ***0.6282*** | 0.0175 | ***0.6552*** | 0.0205 | ***0.6744*** | 0.0192 | ***0.7160*** | 0.0173 | ***0.7332*** | 0.0180 | ***0.7415*** | 0.0188 | ~ | ~ |
| **Bold**, best result.  **Bold** and *italics*, second best result. | | | | | | | | | | | | | | | | |

Table S6-5. Avg. execution time for LFR benchmark network 10000S.

| Mixing  parameter $\mu$ | 0.1 | | 0.15 | | 0.2 | | 0.25 | | 0.3 | | 0.35 | | 0.4 | | 0.45 | |
| --- | --- | --- | --- | --- | --- | --- | --- | --- | --- | --- | --- | --- | --- | --- | --- | --- |
|  | Avg. | Std. | Avg. | Std. | Avg. | Std. | Avg. | Std. | Avg. | Std. | Avg. | Std. | Avg. | Std. | Avg. | Std. |
| Louvain | **0.1674** | 0.0127 | **0.1862** | 0.0156 | **0.2059** | 0.0147 | **0.2330** | 0.0150 | **0.2704** | 0.0214 | **0.2980** | 0.0195 | **0.3328** | 0.0239 | **0.3536** | 0.0286 |
| CNM | 4.4777 | 0.6550 | 8.5254 | 1.8197 | 15.8995 | 0.6875 | 21.3154 | 0.4128 | 26.6979 | 0.5773 | 32.6644 | 0.5340 | 38.7557 | 0.7688 | 44.1096 | 0.5706 |
| DS | 8.0943 | 0.0978 | 10.6361 | 0.1767 | 13.5736 | 0.2510 | 16.9915 | 0.2686 | 20.0559 | 0.3294 | 23.1759 | 0.3830 | 25.6958 | 0.5636 | 27.8320 | 0.4642 |
| INFOMAP | 1.9162 | 0.0291 | 2.0207 | 0.0196 | 2.1091 | 0.0243 | 2.2168 | 0.0294 | 2.2672 | 0.0387 | 2.3655 | 0.0366 | 2.4846 | 0.0386 | 2.6520 | 0.0951 |
| HAM_Cosine_ | ***1.0286*** | 0.0276 | ***1.1216*** | 0.0243 | ***1.1861*** | 0.0410 | ***1.2724*** | 0.0350 | ***1.3229*** | 0.0271 | ***1.3666*** | 0.0295 | ***1.4394*** | 0.0238 | ***1.4602*** | 0.0308 |
|  |  |  |  |  |  |  |  |  |  |  |  |  |  |  |  |  |
| Mixing  parameter $\mu$ | 0.5 | | 0.55 | | 0.6 | | 0.65 | | 0.7 | | 0.75 | | 0.8 | | ~ | |
|  | Avg. | Std. | Avg. | Std. | Avg. | Std. | Avg. | Std. | Avg. | Std. | Avg. | Std. | Avg. | Std. | ~ | ~ |
| Louvain | **0.4020** | 0.0321 | **0.4420** | 0.0379 | **0.4841** | 0.0431 | **0.5450** | 0.0485 | **0.6204** | 0.0556 | **0.9256** | 0.1666 | **0.7961** | 0.1068 | ~ | ~ |
| CNM | 48.4563 | 0.5234 | 52.4879 | 0.5544 | 56.0020 | 0.6601 | 57.8163 | 0.5697 | 58.1704 | 0.6839 | 56.0140 | 1.4146 | 47.2800 | 2.9149 | ~ | ~ |
| DS | 28.5980 | 0.5920 | 28.3635 | 0.8315 | 26.4857 | 0.9691 | 19.7834 | 0.6729 | 12.6859 | 0.8729 | 6.9077 | 0.4834 | 4.3612 | 0.1544 | ~ | ~ |
| INFOMAP | 2.8579 | 0.1165 | 3.1730 | 0.1317 | 3.5802 | 0.1794 | 4.2297 | 0.2015 | 5.4678 | 0.3957 | 8.2561 | 0.5979 | 42.5496 | 9.3185 | ~ | ~ |
| HAM_Cosine_ | ***1.5044*** | 0.0251 | ***1.5590*** | 0.0307 | ***1.6063*** | 0.0289 | ***1.6520*** | 0.0361 | ***1.7176*** | 0.0280 | ***1.7820*** | 0.0284 | ***1.8008*** | 0.0343 | ~ | ~ |
| **Bold**, best result.  **Bold** and *italics*, second best result. | | | | | | | | | | | | | | | | |

Table S6-6. Avg. execution time for LFR benchmark network 10000B.

| Mixing  parameter $\mu$ | 0.1 | | 0.15 | | 0.2 | | 0.25 | | 0.3 | | 0.35 | | 0.4 | | 0.45 | |
| --- | --- | --- | --- | --- | --- | --- | --- | --- | --- | --- | --- | --- | --- | --- | --- | --- |
|  | Avg. | Std. | Avg. | Std. | Avg. | Std. | Avg. | Std. | Avg. | Std. | Avg. | Std. | Avg. | Std. | Avg. | Std. |
| Louvain | **0.1700** | 0.0163 | **0.1950** | 0.0192 | **0.2288** | 0.0172 | **0.2444** | 0.0225 | **0.2803** | 0.0215 | **0.3063** | 0.0237 | **0.3500** | 0.0321 | **0.3760** | 0.0474 |
| CNM | 13.3146 | 2.4531 | 21.1828 | 0.9709 | 27.6058 | 0.6817 | 35.6549 | 0.7457 | 42.5548 | 0.4626 | 49.3039 | 0.8169 | 55.5434 | 0.6351 | 60.3893 | 0.6437 |
| DS | 35.1047 | 0.3508 | 44.6598 | 0.4105 | 53.0453 | 0.7136 | 60.8760 | 0.4946 | 68.0021 | 0.4955 | 74.4439 | 0.6099 | 79.9611 | 0.6154 | 83.8449 | 0.7151 |
| INFOMAP | 3.0919 | 0.0372 | 3.1658 | 0.0447 | 3.2271 | 0.0371 | 3.2937 | 0.0336 | 3.3821 | 0.0500 | 3.3961 | 0.0580 | 3.5672 | 0.0521 | 3.6670 | 0.1444 |
| HAM_Cosine_ | ***2.1252*** | 0.0433 | ***2.0998*** | 0.0377 | ***2.0665*** | 0.0418 | ***2.0441*** | 0.0472 | ***2.0431*** | 0.0387 | ***2.0800*** | 0.0510 | ***2.0935*** | 0.0321 | ***2.1689*** | 0.0484 |
|  |  |  |  |  |  |  |  |  |  |  |  |  |  |  |  |  |
| Mixing  parameter $\mu$ | 0.5 | | 0.55 | | 0.6 | | 0.65 | | 0.7 | | 0.75 | | 0.8 | | ~ | |
|  | Avg. | Std. | Avg. | Std. | Avg. | Std. | Avg. | Std. | Avg. | Std. | Avg. | Std. | Avg. | Std. | ~ | ~ |
| Louvain | **0.4222** | 0.0500 | **0.4618** | 0.0444 | **0.5299** | 0.0666 | **0.5954** | 0.0624 | **0.6750** | 0.0777 | **0.7488** | 0.1121 | **1.6515** | 0.3410 | ~ | ~ |
| CNM | 64.7776 | 0.7311 | 68.5335 | 0.4544 | 70.6972 | 1.6071 | 73.3908 | 0.4647 | 74.7761 | 0.4980 | 75.2915 | 1.1477 | 72.3377 | 2.8943 | ~ | ~ |
| DS | 86.6998 | 0.9227 | 87.3269 | 0.9399 | 86.6197 | 1.1691 | 82.5387 | 1.1024 | 70.7373 | 1.6822 | 50.1130 | 2.1133 | 32.7169 | 1.4761 | ~ | ~ |
| INFOMAP | 3.8813 | 0.1341 | 4.0612 | 0.0991 | 4.2708 | 0.0857 | 4.6290 | 0.1745 | 5.2697 | 0.2021 | 6.5089 | 0.2563 | 10.1265 | 1.6447 | ~ | ~ |
| HAM_Cosine_ | ***2.2448*** | 0.0374 | ***2.3572*** | 0.0345 | ***2.4580*** | 0.0314 | ***2.5813*** | 0.0341 | ***2.6894*** | 0.0308 | ***2.8475*** | 0.0360 | ***3.0046*** | 0.0464 | ~ | ~ |
| **Bold**, best result.  **Bold** and *italics*, second best result. | | | | | | | | | | | | | | | | |

Table S6-7. Avg. execution time for LFR benchmark network 50000S.

| Mixing  parameter $\mu$ | 0.1 | | 0.15 | | 0.2 | | 0.25 | | 0.3 | | 0.35 | | 0.4 | | 0.45 | |
| --- | --- | --- | --- | --- | --- | --- | --- | --- | --- | --- | --- | --- | --- | --- | --- | --- |
|  | Avg. | Std. | Avg. | Std. | Avg. | Std. | Avg. | Std. | Avg. | Std. | Avg. | Std. | Avg. | Std. | Avg. | Std. |
| Louvain | **0.9859** | 0.1343 | **1.2532** | 0.0632 | **1.5964** | 0.1184 | **1.8075** | 0.0917 | **1.9864** | 0.0949 | **2.3088** | 0.2177 | **2.4970** | 0.2021 | **2.8501** | 0.2478 |
| CNM | - | - | - | - | - | - | - | - | - | - | - | - | - | - | - | - |
| DS | * | * | * | * | * | * | * | * | * | * | * | * | * | * | * | * |
| INFOMAP | 16.9676 | 0.1201 | 17.8319 | 0.0876 | 18.6238 | 0.1242 | 19.5292 | 0.0995 | 20.3045 | 0.1348 | 21.2150 | 0.1216 | 22.1364 | 0.1153 | 23.1536 | 0.4808 |
| HAM_Cosine_ | ***12.4561*** | 0.2133 | ***12.8165*** | 0.1927 | ***13.4467*** | 0.3022 | ***13.9173*** | 0.1314 | ***14.0203*** | 0.0955 | ***14.3479*** | 0.2372 | ***14.6942*** | 0.2187 | ***15.1897*** | 0.0602 |
|  |  |  |  |  |  |  |  |  |  |  |  |  |  |  |  |  |
| Mixing  parameter $\mu$ | 0.5 | | 0.55 | | 0.6 | | 0.65 | | 0.7 | | 0.75 | | 0.8 | | ~ | |
|  | Avg. | Std. | Avg. | Std. | Avg. | Std. | Avg. | Std. | Avg. | Std. | Avg. | Std. | Avg. | Std. | ~ | ~ |
| Louvain | **3.2500** | 0.2733 | **3.5188** | 0.2571 | **3.9021** | 0.3341 | **4.4242** | 0.3856 | **4.9145** | 0.2977 | **5.7632** | 0.5116 | **6.6415** | 0.4630 | ~ | ~ |
| CNM | - | - | - | - | - | - | - | - | - | - | - | - | - | - | ~ | ~ |
| DS | * | * | * | * | * | * | * | * | * | * | * | * | * | * | ~ | ~ |
| INFOMAP | 24.1962 | 0.4710 | 25.4634 | 0.1799 | 27.1326 | 0.5892 | 29.4248 | 1.1220 | 33.3809 | 1.2654 | 40.6058 | 1.7775 | 55.3286 | 3.2796 | ~ | ~ |
| HAM_Cosine_ | ***15.6484*** | 0.1185 | ***16.1569*** | 0.1119 | ***16.6827*** | 0.0739 | ***17.2594*** | 0.0737 | ***17.9884*** | 0.0789 | ***18.6883*** | 0.0690 | ***19.8562*** | 0.0929 | ~ | ~ |
| **Bold**, best result.  **Bold** and *italics*, second best result.  -, runs exceeded at least 1 hours for 30 networks and would cost many hours (or days) for all sets of networks.  *, memory allocation error (e.g., “std::bad_alloc”). | | | | | | | | | | | | | | | | |

Table S6-8. Avg. execution time for LFR benchmark network 50000B.

| Mixing  parameter $\mu$ | 0.1 | | 0.15 | | 0.2 | | 0.25 | | 0.3 | | 0.35 | | 0.4 | | 0.45 | |
| --- | --- | --- | --- | --- | --- | --- | --- | --- | --- | --- | --- | --- | --- | --- | --- | --- |
|  | Avg. | Std. | Avg. | Std. | Avg. | Std. | Avg. | Std. | Avg. | Std. | Avg. | Std. | Avg. | Std. | Avg. | Std. |
| Louvain | **0.9022** | 0.1147 | **0.9558** | 0.0744 | **1.2839** | 0.0790 | **1.3582** | 0.1526 | **1.5912** | 0.1593 | **1.7269** | 0.1829 | **1.9594** | 0.1859 | **2.2807** | 0.2434 |
| CNM | - | - | - | - | - | - | - | - | - | - | - | - | - | - | - | - |
| DS | * | * | * | * | * | * | * | * | * | * | * | * | * | * | * | * |
| INFOMAP | 16.2693 | 0.2841 | 16.9531 | 0.1168 | 17.4382 | 0.0989 | 17.9811 | 0.1376 | 18.3410 | 0.0956 | 18.9213 | 0.3205 | 19.6118 | 0.3329 | 20.3809 | 0.3604 |
| HAM_Cosine_ | ***12.2575*** | 0.0807 | ***12.3687*** | 0.0989 | ***12.4878*** | 0.1317 | ***12.5440*** | 0.1033 | ***12.4899*** | 0.0795 | ***12.5726*** | 0.1018 | ***12.6443*** | 0.1412 | ***12.9823*** | 0.1142 |
|  |  |  |  |  |  |  |  |  |  |  |  |  |  |  |  |  |
| Mixing  parameter $\mu$ | 0.5 | | 0.55 | | 0.6 | | 0.65 | | 0.7 | | 0.75 | | 0.8 | | ~ | |
|  | Avg. | Std. | Avg. | Std. | Avg. | Std. | Avg. | Std. | Avg. | Std. | Avg. | Std. | Avg. | Std. | ~ | ~ |
| Louvain | **2.5116** | 0.3315 | **2.6239** | 0.2988 | **2.9401** | 0.1971 | **3.3821** | 0.3855 | **3.5854** | 0.3966 | **4.1662** | 0.5069 | **8.5369** | 1.8905 | ~ | ~ |
| CNM | - | - | - | - | - | - | - | - | - | - | - | - | - | - | ~ | ~ |
| DS | * | * | * | * | * | * | * | * | * | * | * | * | * | * | ~ | ~ |
| INFOMAP | 20.9810 | 0.4459 | 21.9414 | 0.3755 | 23.7271 | 0.8878 | 26.4088 | 1.0972 | 30.0488 | 1.2834 | 36.2019 | 1.3622 | 46.3950 | 2.9394 | ~ | ~ |
| HAM_Cosine_ | ***13.1441*** | 0.0741 | ***13.5367*** | 0.0799 | ***14.0842*** | 0.1092 | ***14.6879*** | 0.0666 | ***15.4404*** | 0.0948 | ***16.2063*** | 0.0658 | ***16.9068*** | 0.0930 | ~ | ~ |
| **Bold**, best result.  **Bold** and *italics*, second best result.  -, runs exceeded at least 1 hours for 30 networks and would cost many hours (or days) for all sets of networks.  *, memory allocation error (e.g., “std::bad_alloc”). | | | | | | | | | | | | | | | | |
