# Supplementary material for: A community detection algorithm using network topologies and rule-based hierarchical arc-merging strategies
Source: PLoS One. 2017 Nov 9;12(11):e0187603. doi: 10.1371/journal.pone.0187603 (PMC5679540; doi:10.1371/journal.pone.0187603)
Supplement: S7 File — (DOCX) [file pone.0187603.s007.docx]

**S7 File. Summarized results of large-scale real metworks.**

Table A. Summary of each method for large-scale real networks.

| Method | Q | | | Time | | |
| --- | --- | --- | --- | --- | --- | --- |
|  | # of 1^st^ place | # of 2^st^ place | **Final place** | # of 1^st^ place | # of 2^st^ place | **Final place** |
| Louvain | 4 | 2 | **1** | 8 | - | **1** |
| CNM | - | 1 | 4 | - | - | - |
| DS | - | 1 | 4 | - | - | - |
| INFOMAP | 1 | - | 3 | - | - | - |
| HAM_Cosine_ | 3 | 4 | **2** | - | 8 | **2** |

Table B. Summary of final place for large-scale real networks

| Method | # of 1^st^ place | # of 2^st^ place | **Final place** |
| --- | --- | --- | --- |
| Louvain | 2 | - | **1** |
| CNM | - | - | - |
| DS | - | - | - |
| INFOMAP | - | - | - |
| HAM_Cosine_ | - | 2 | **2** |
