# Supplementary material for: A community detection algorithm using network topologies and rule-based hierarchical arc-merging strategies
Source: PLoS One. 2017 Nov 9;12(11):e0187603. doi: 10.1371/journal.pone.0187603 (PMC5679540; doi:10.1371/journal.pone.0187603)
Supplement: S8 File — (DOCX) [file pone.0187603.s008.docx]

**S8 File. Multi-resolution analysis of different similarities for small-scale networks.**

| 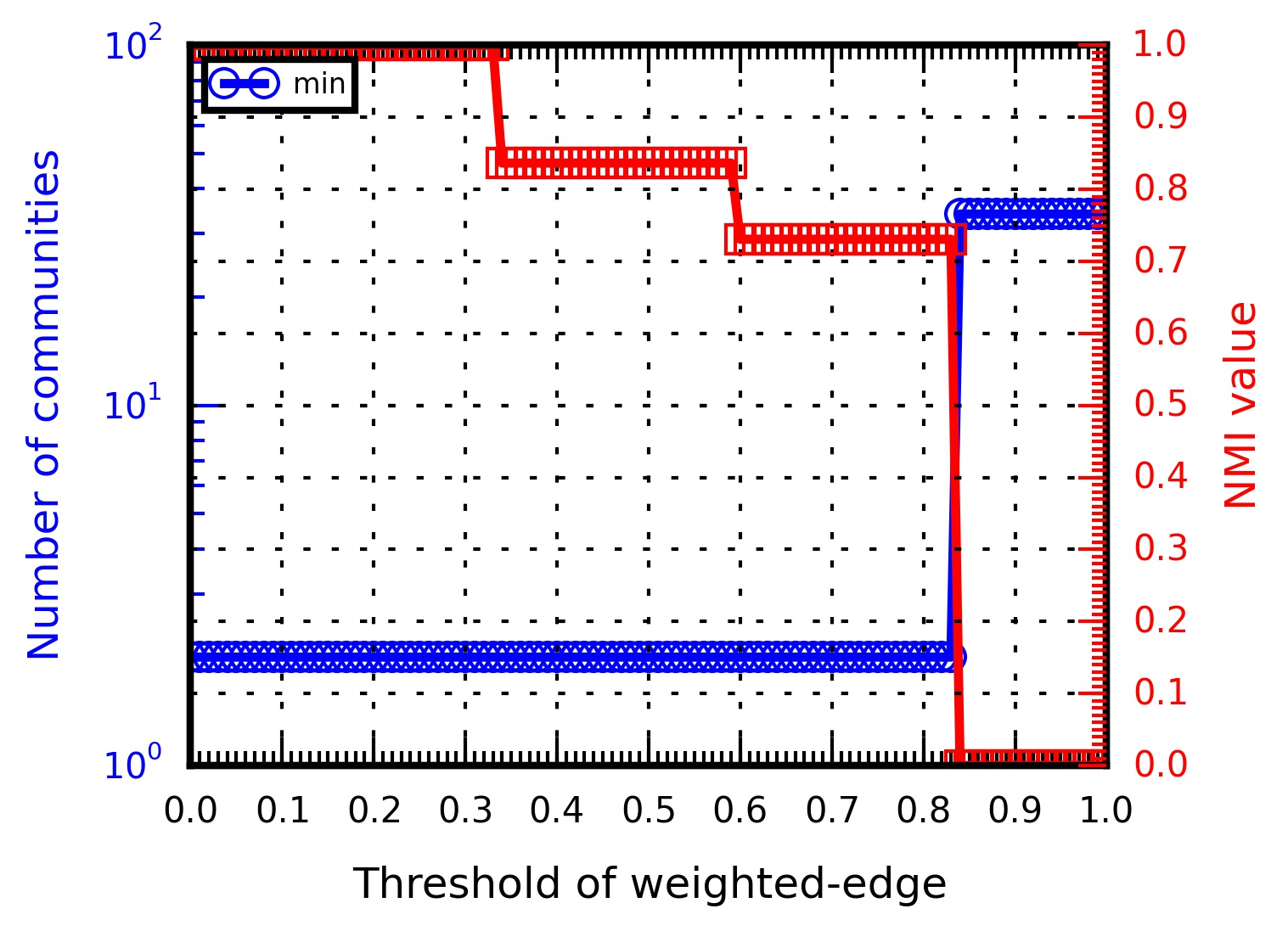 | 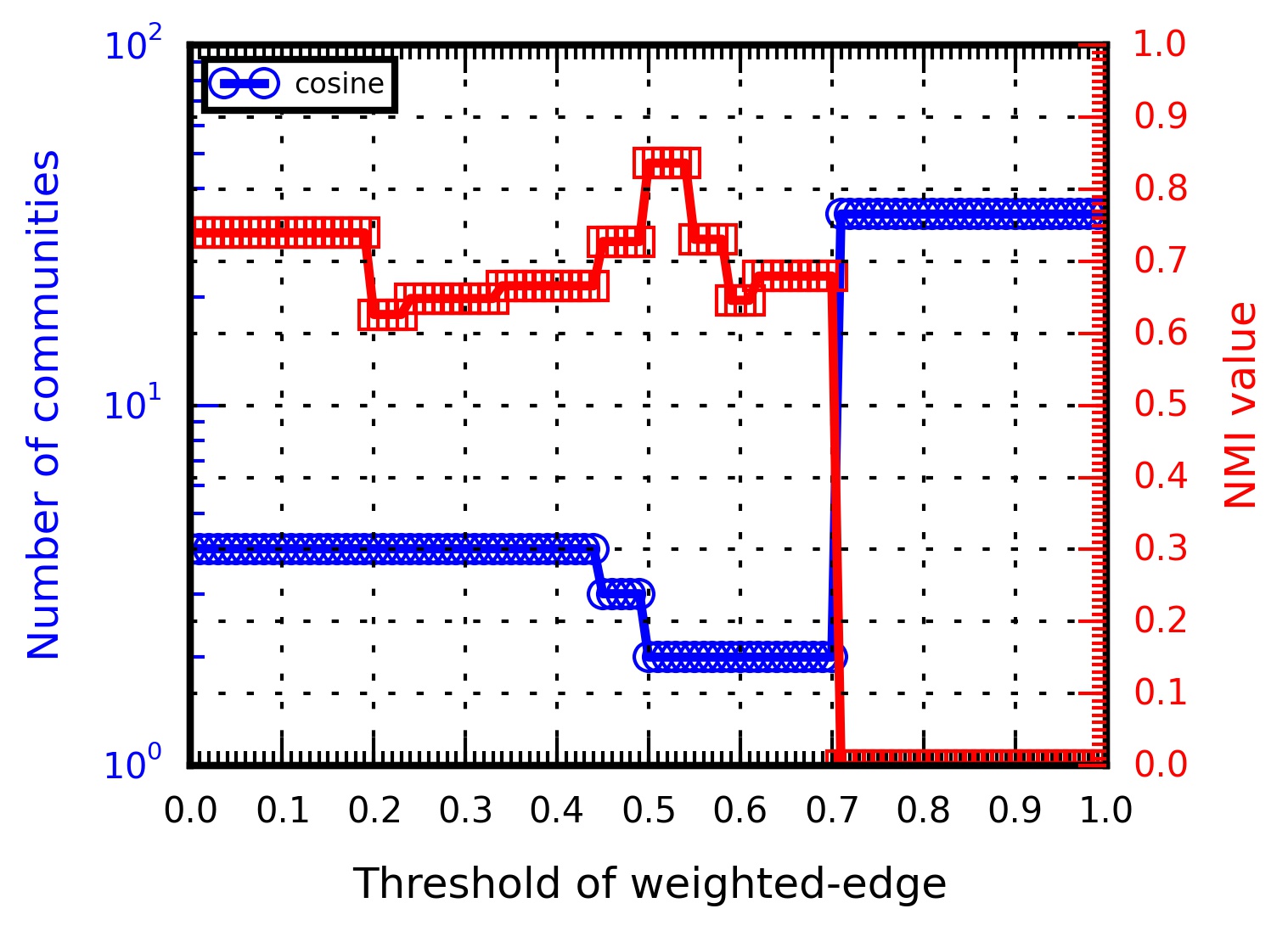 | 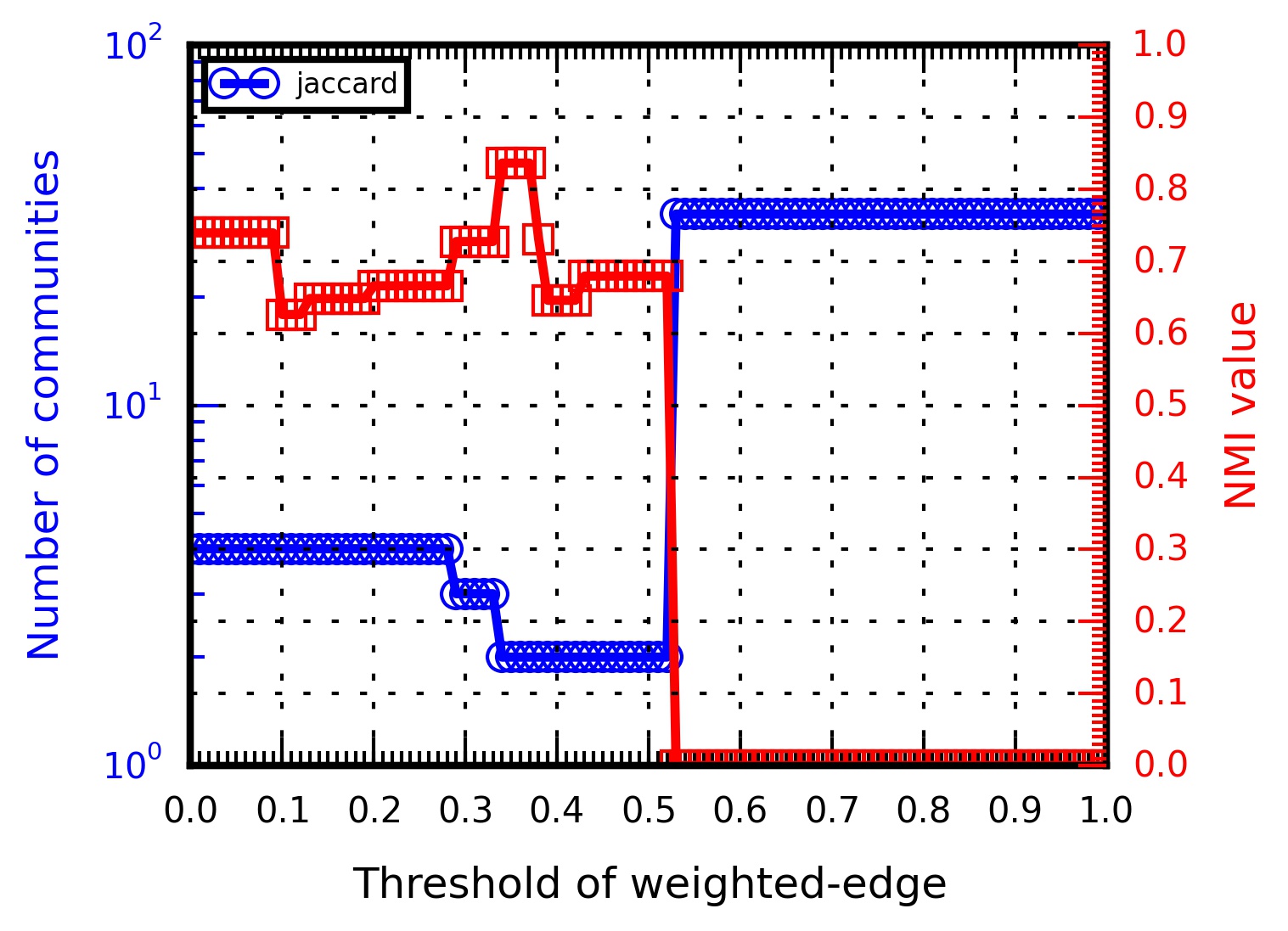 |
| --- | --- | --- |
| (a) Minimum similarity | (b) Cosine similarity | (c) Jaccard index |
| **Fig S8-1. Multi-resolution analysis of different similarities for Karate network.** (a) Minimum similarity, (b) Cosine similarity, (c) Jaccard index. | | |

| 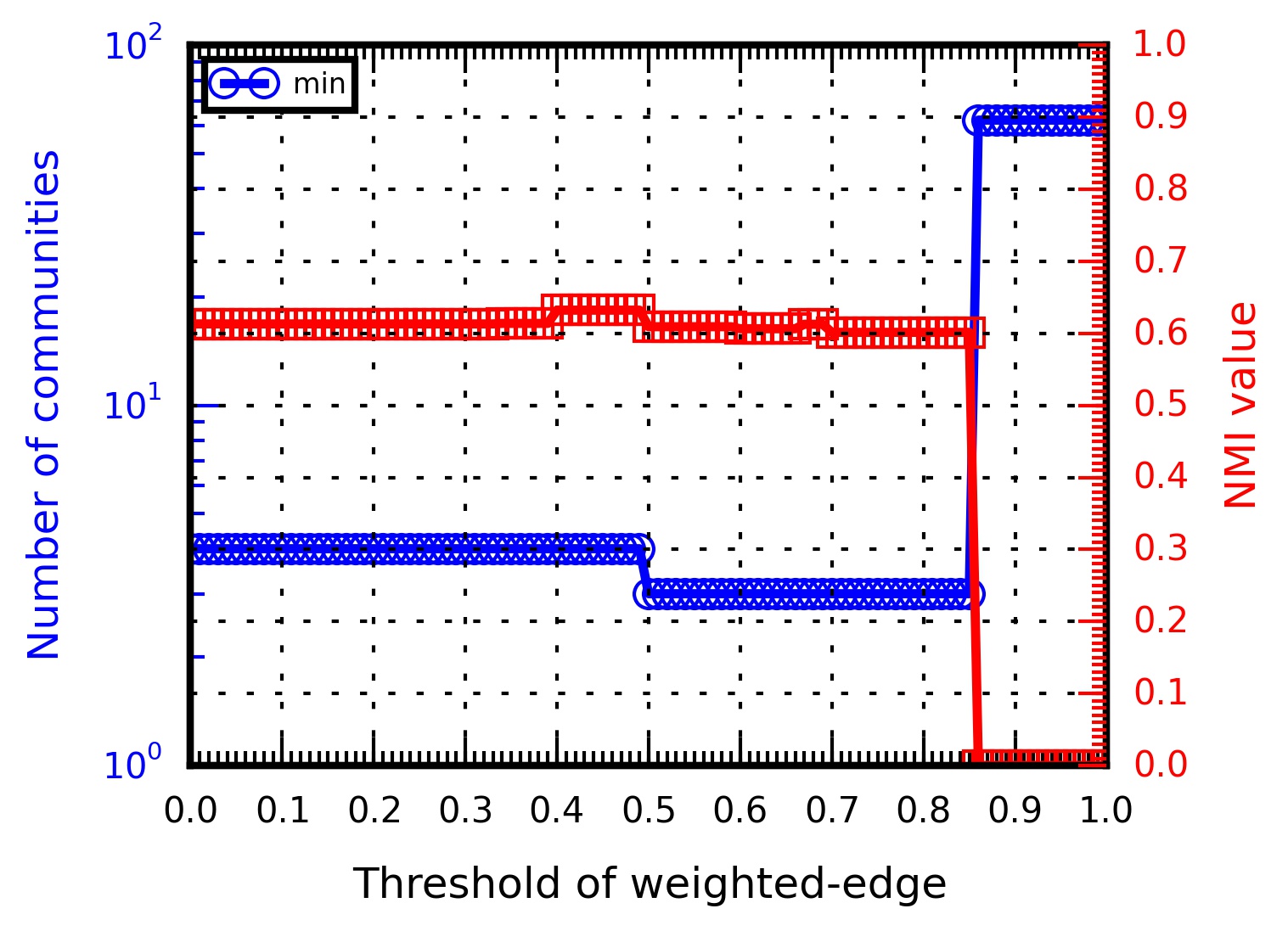 | 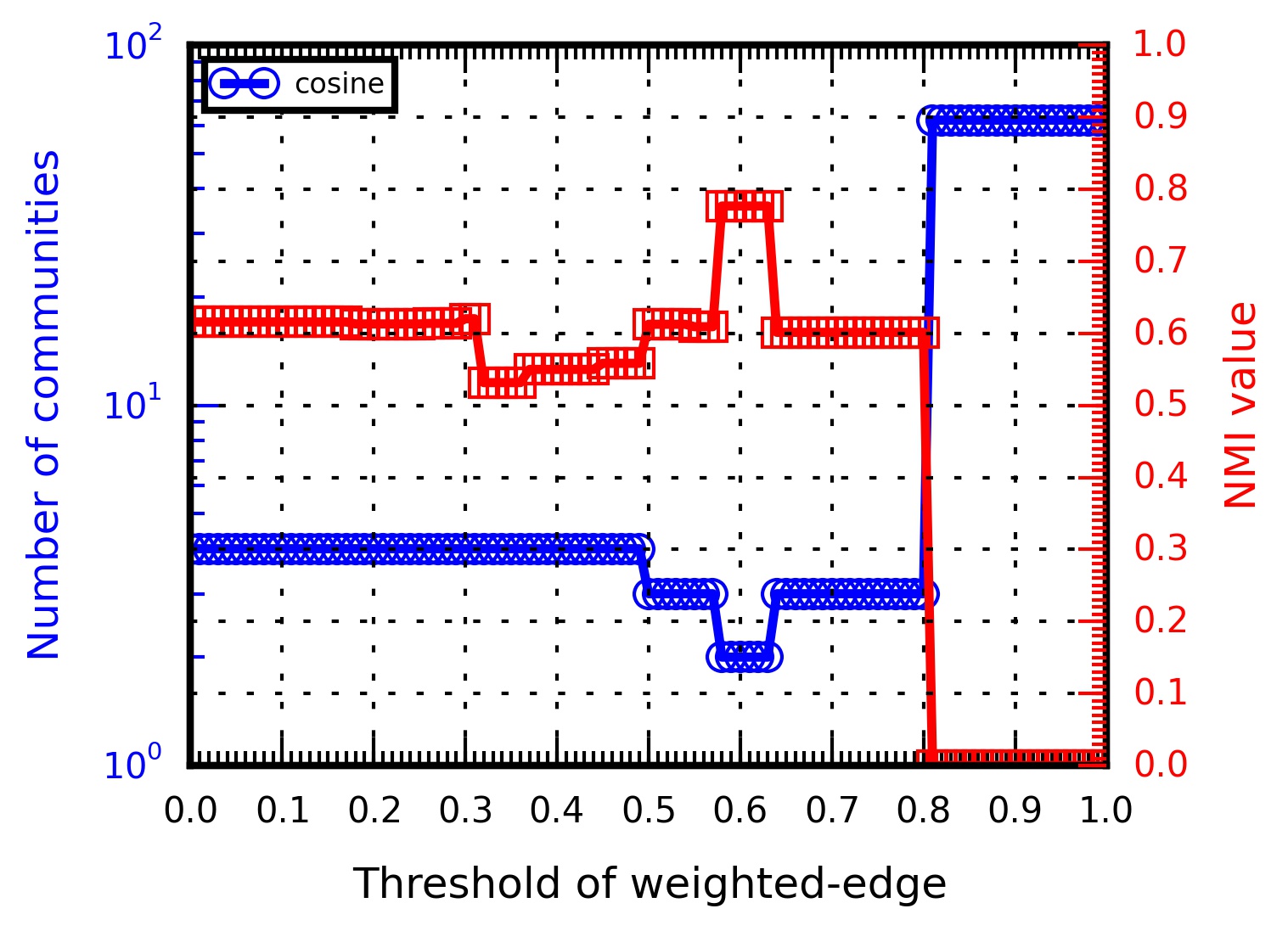 | 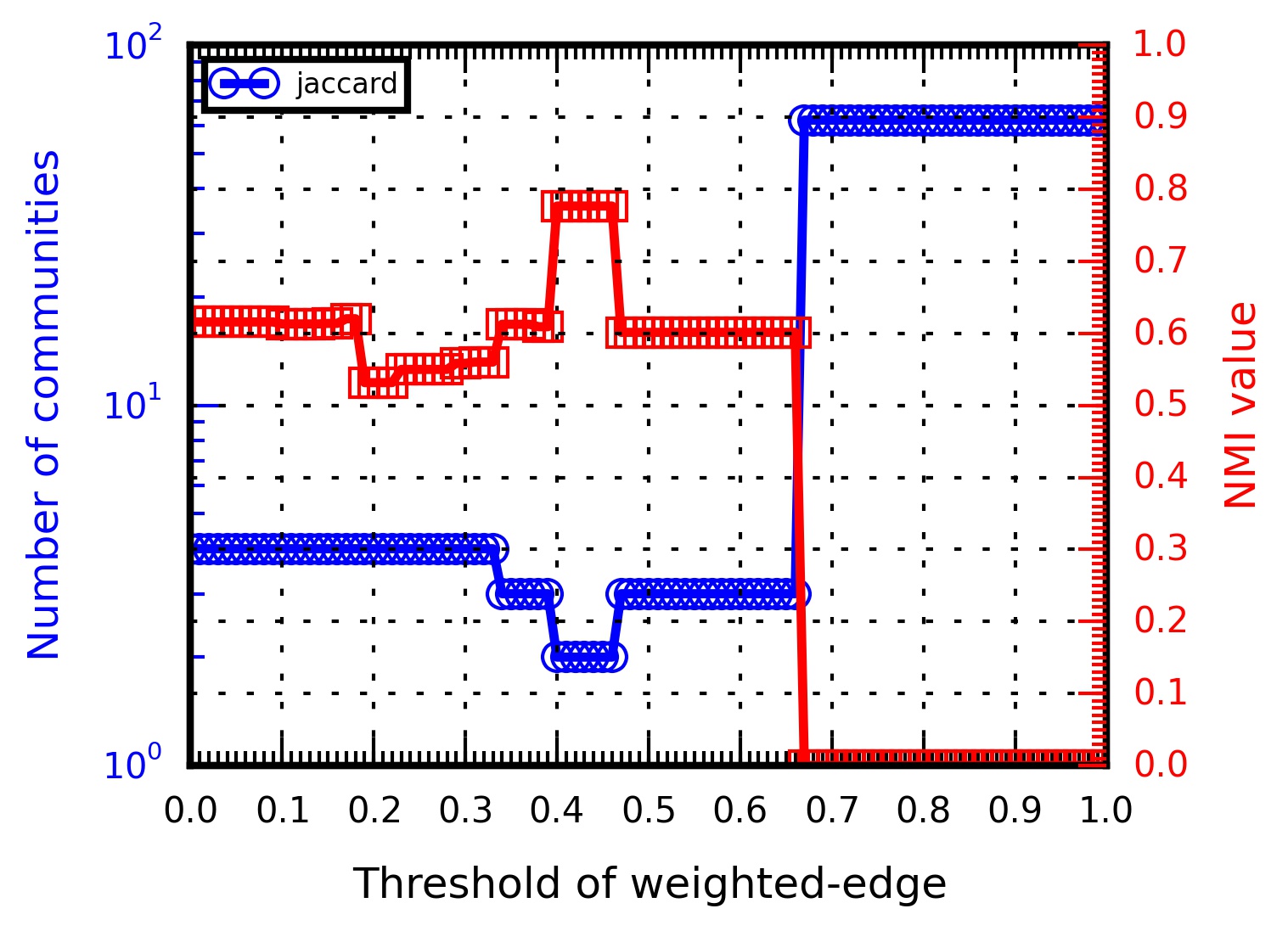 |
| --- | --- | --- |
| (a) Minimum similarity | (b) Cosine similarity | (c) Jaccard index |
| **Fig S8-2. Multi-resolution analysis of different similarities for Dolphins network.** (a) Minimum similarity, (b) Cosine similarity, (c) Jaccard index. | | |

| 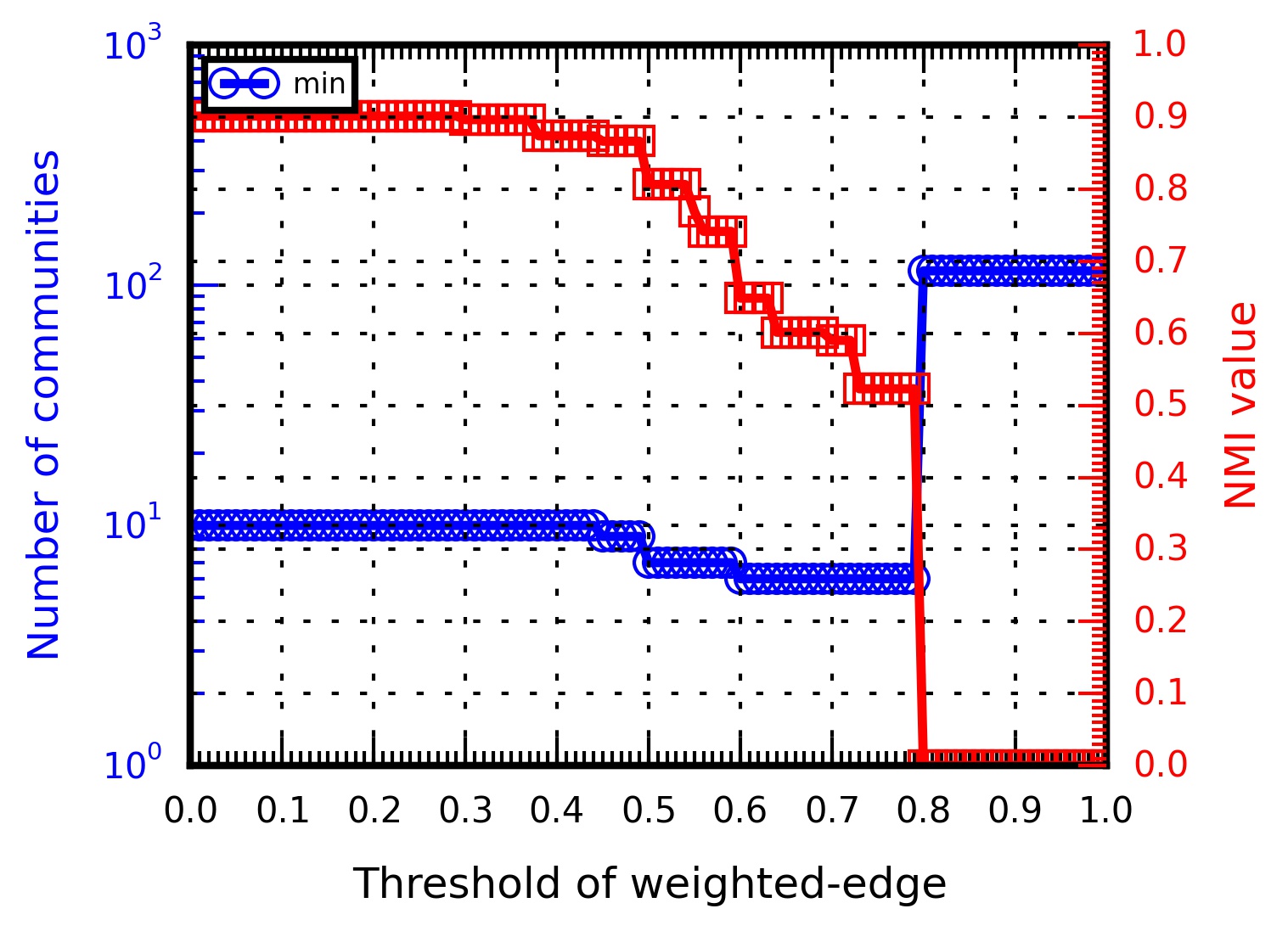 | 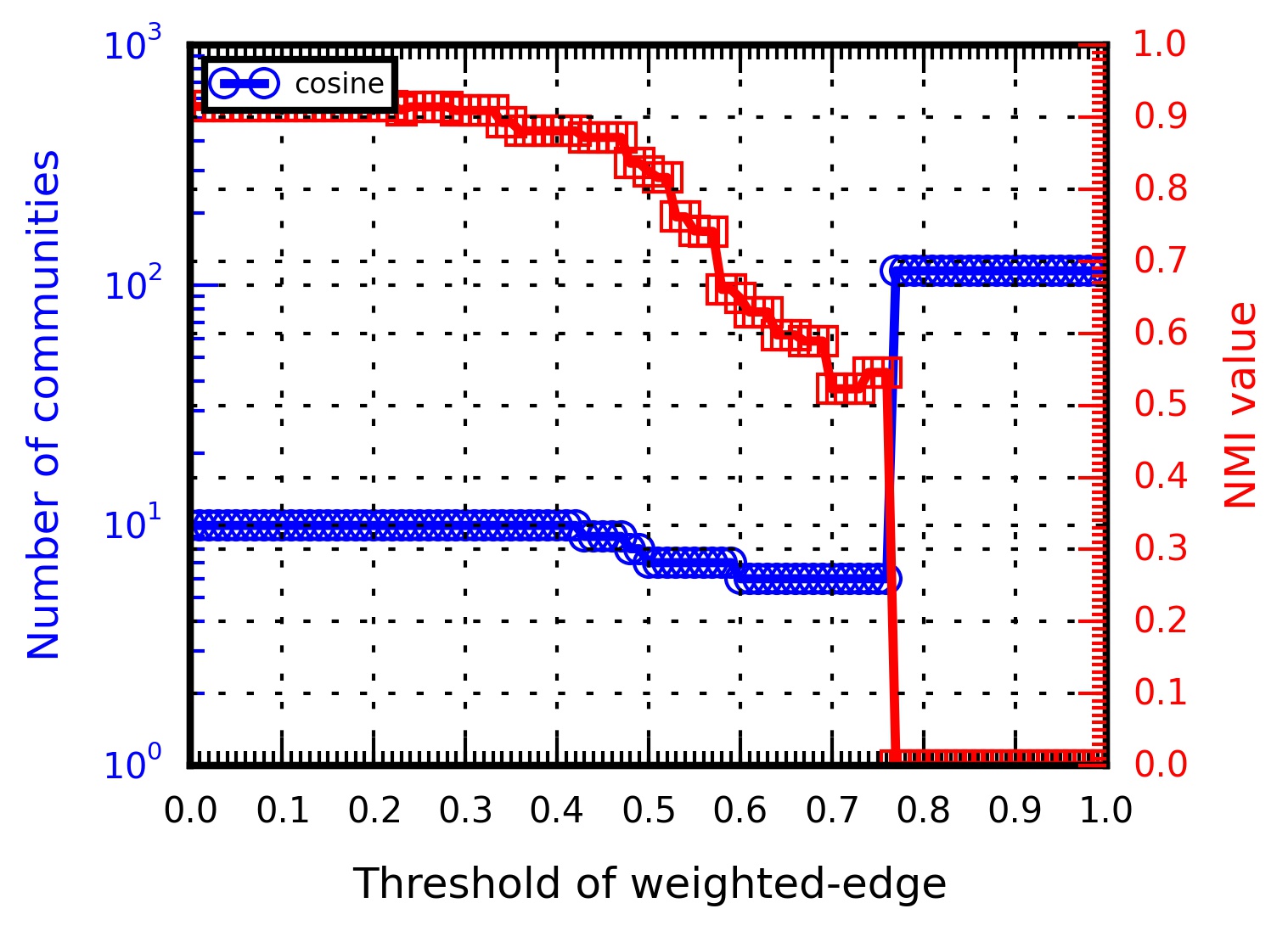 | 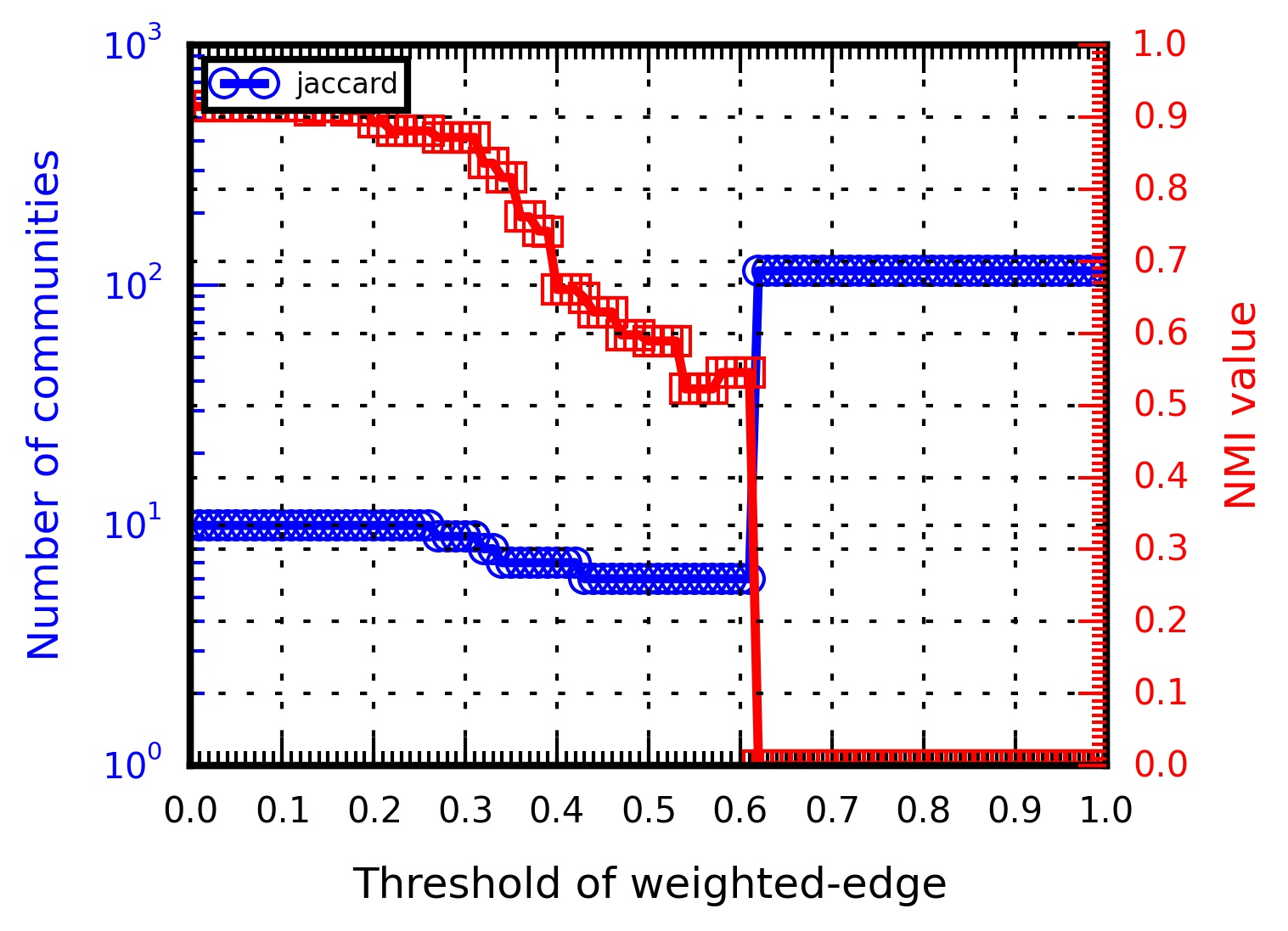 |
| --- | --- | --- |
| (a) Minimum similarity | (b) Cosine similarity | (c) Jaccard index |
| **Fig S8-3. Multi-resolution analysis of different similarities for Football network.** (a) Minimum similarity, (b) Cosine similarity, (c) Jaccard index. | | |

| 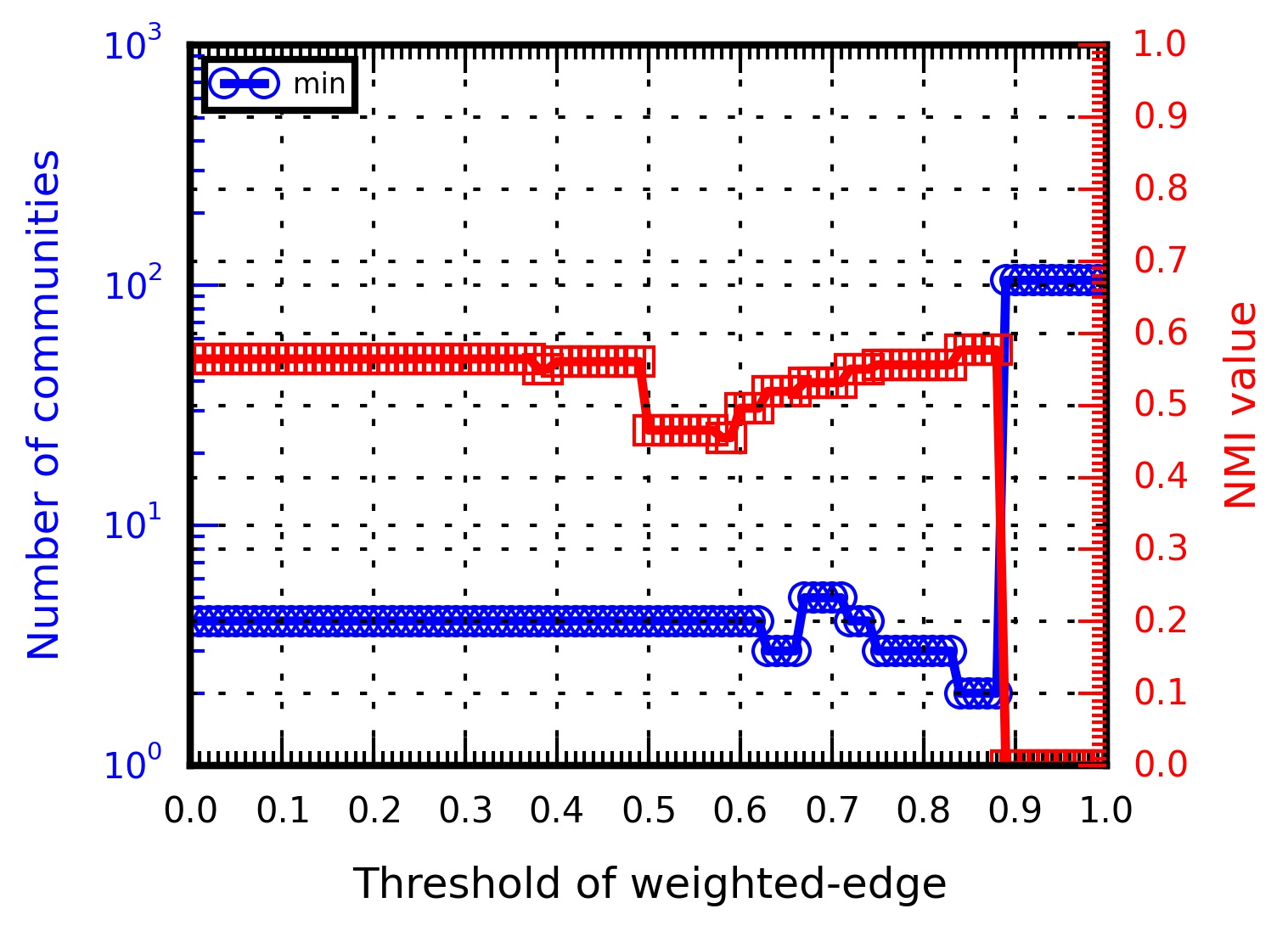 | 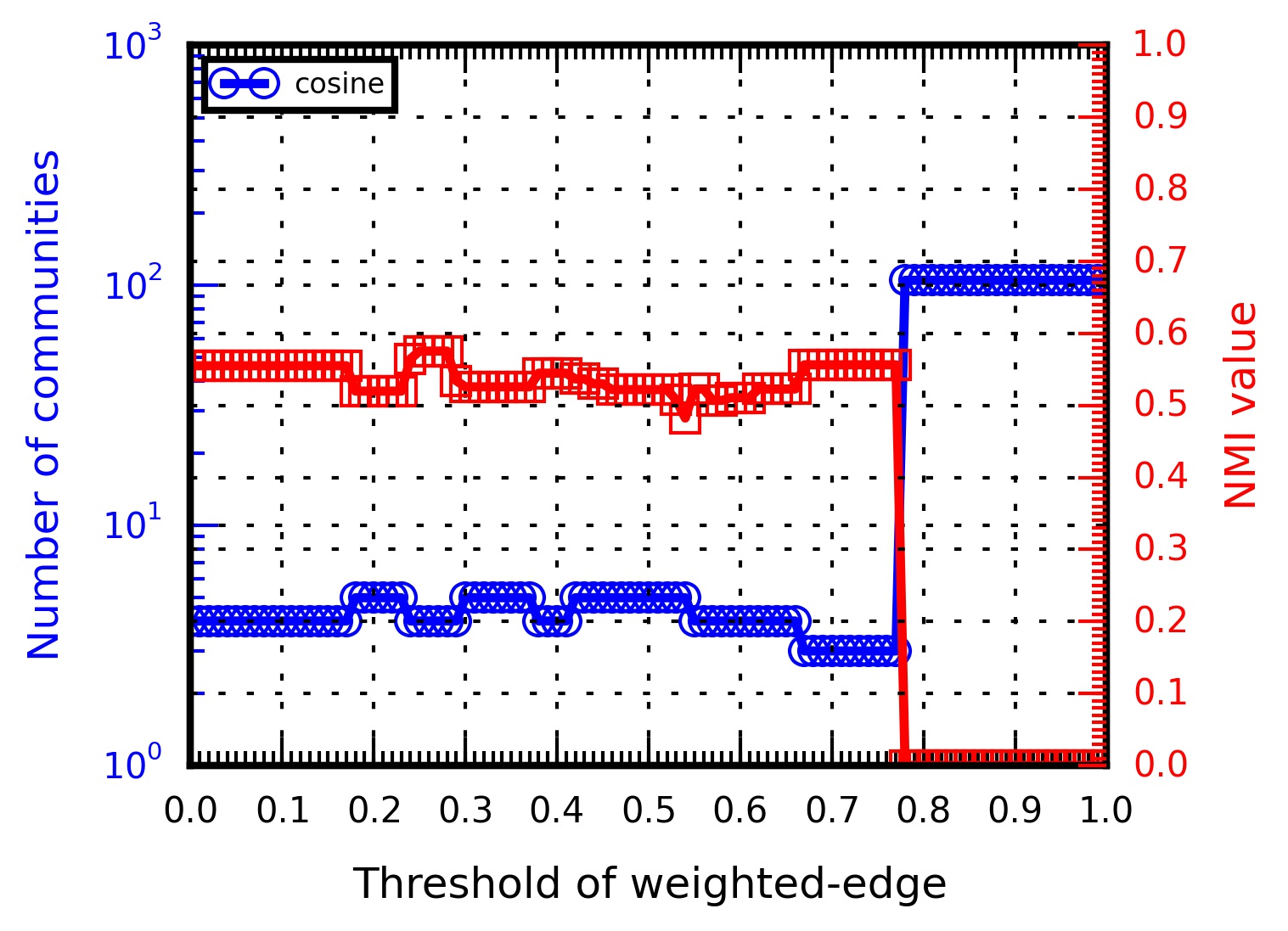 | 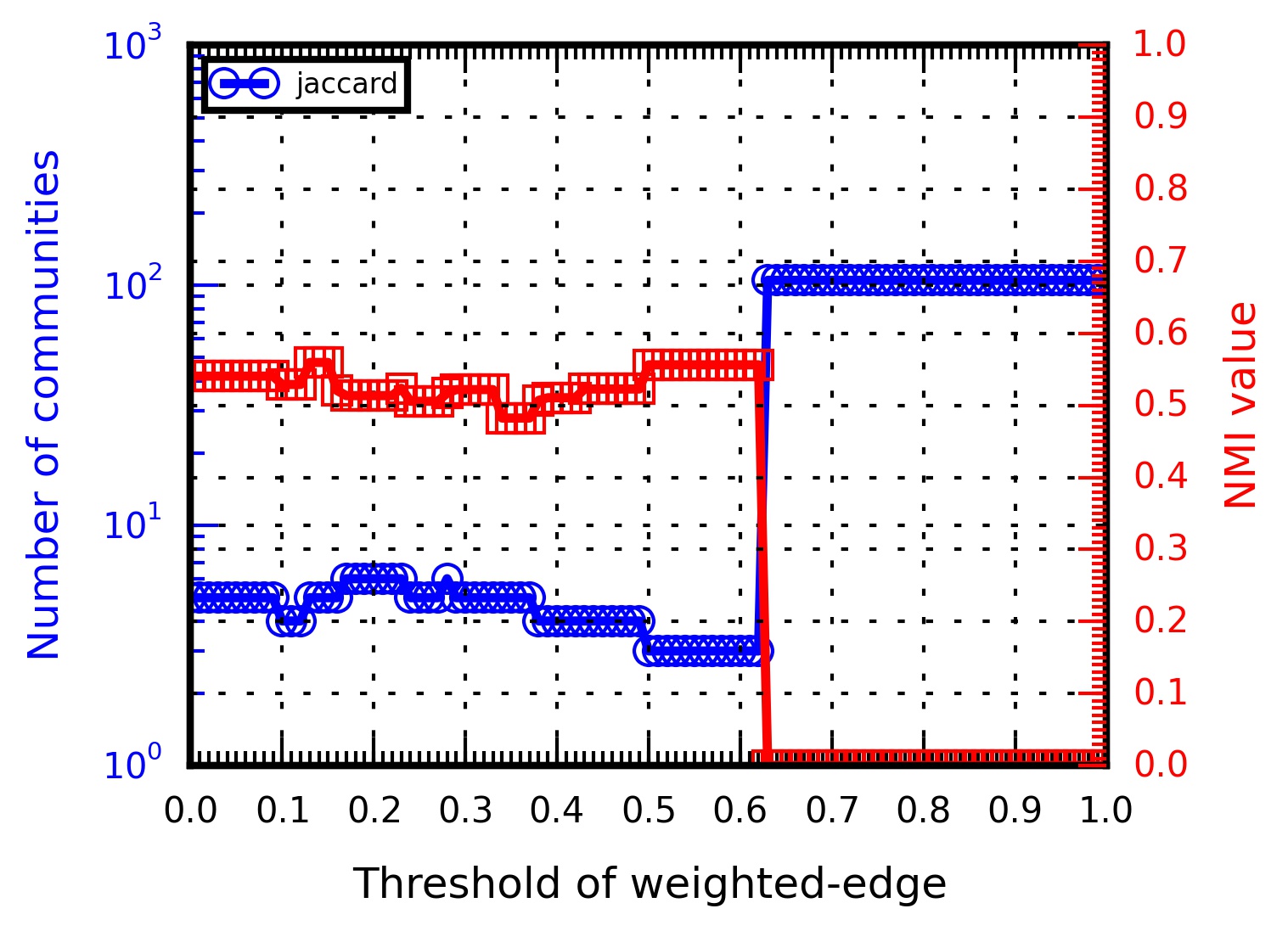 |
| --- | --- | --- |
| (a) Minimum similarity | (b) Cosine similarity | (c) Jaccard index |
| Fig S8-4. Multi-resolution analysis of different similarities for Polbooks network. | | |

| 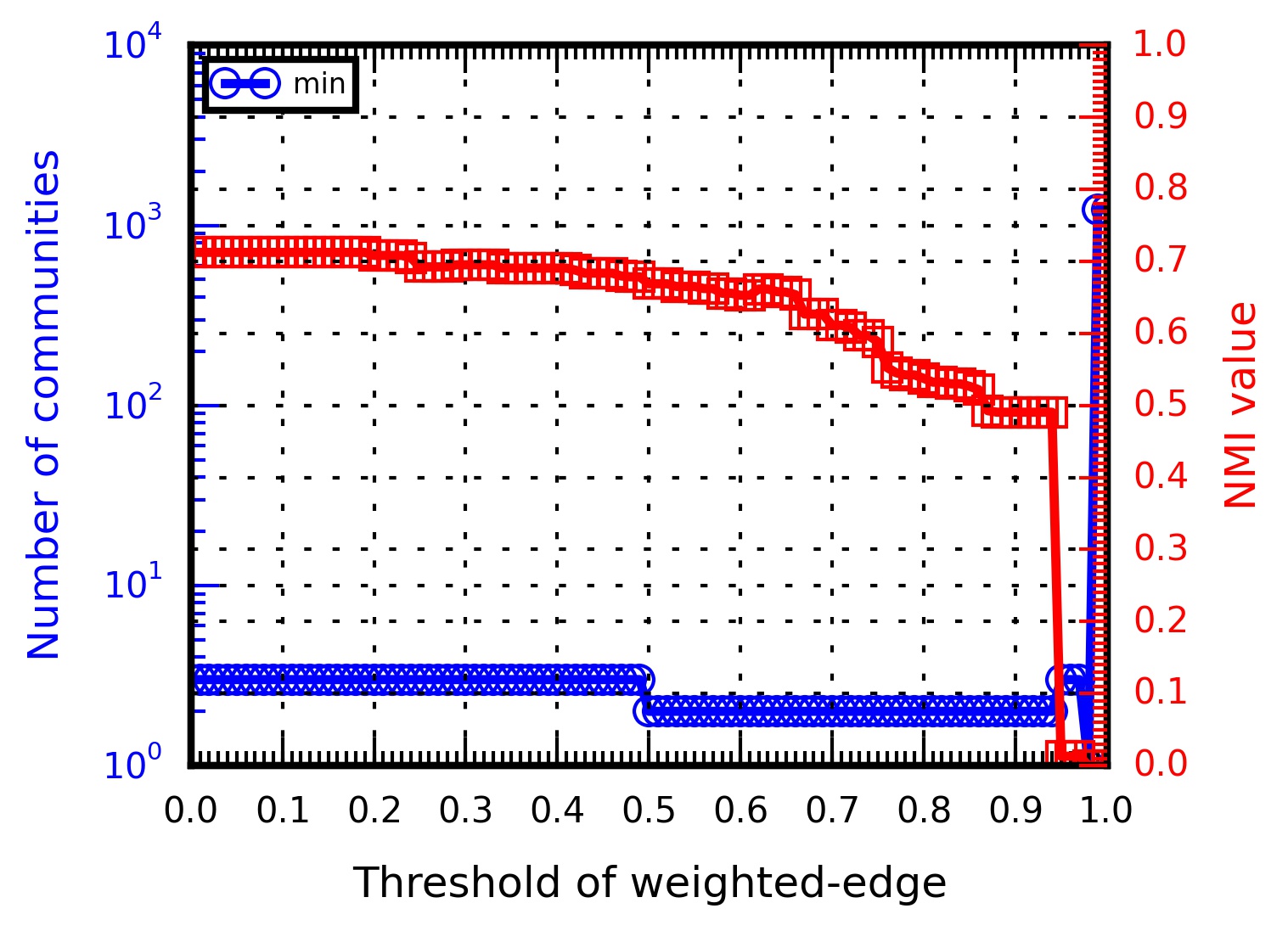 | 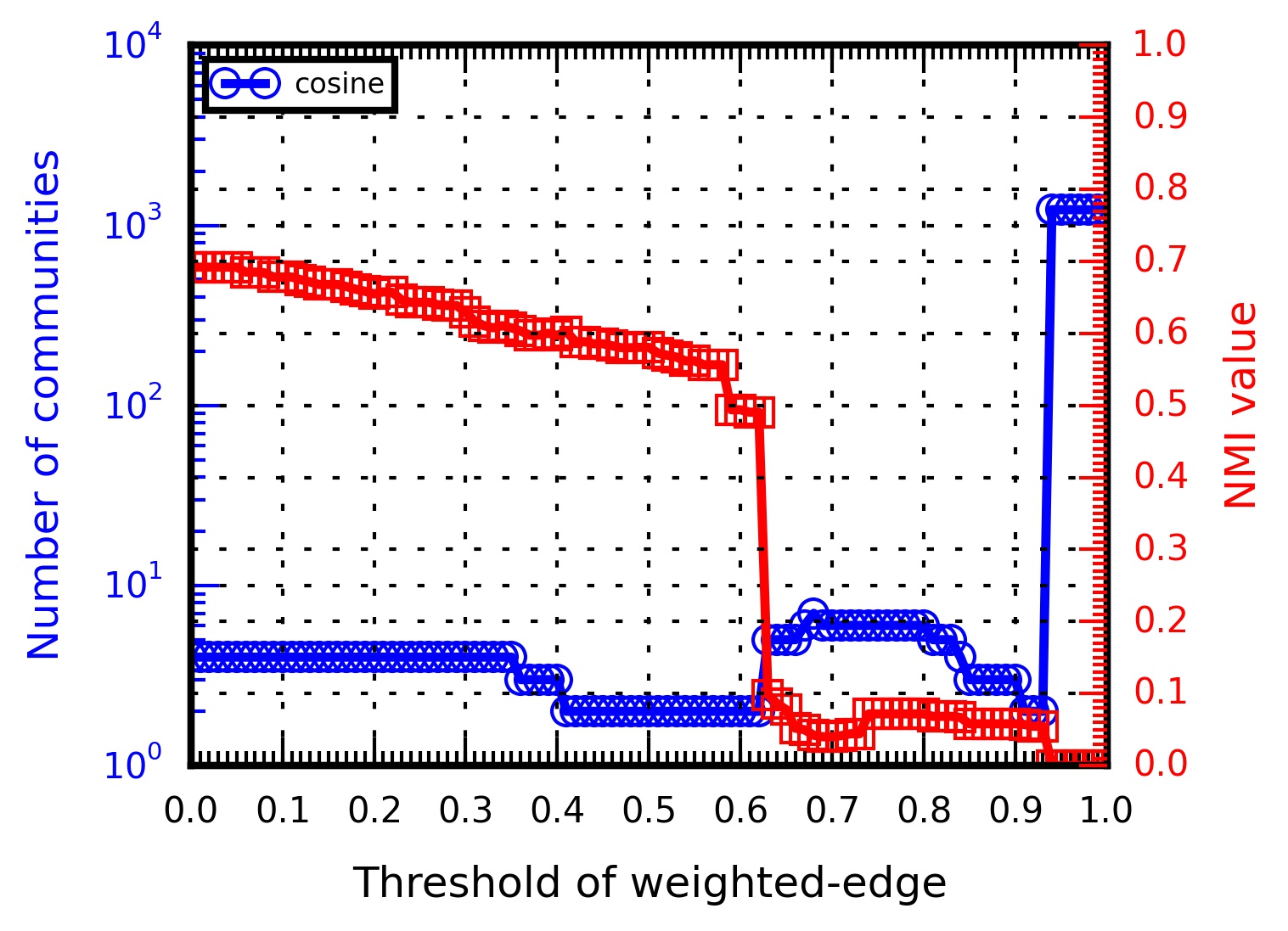 | 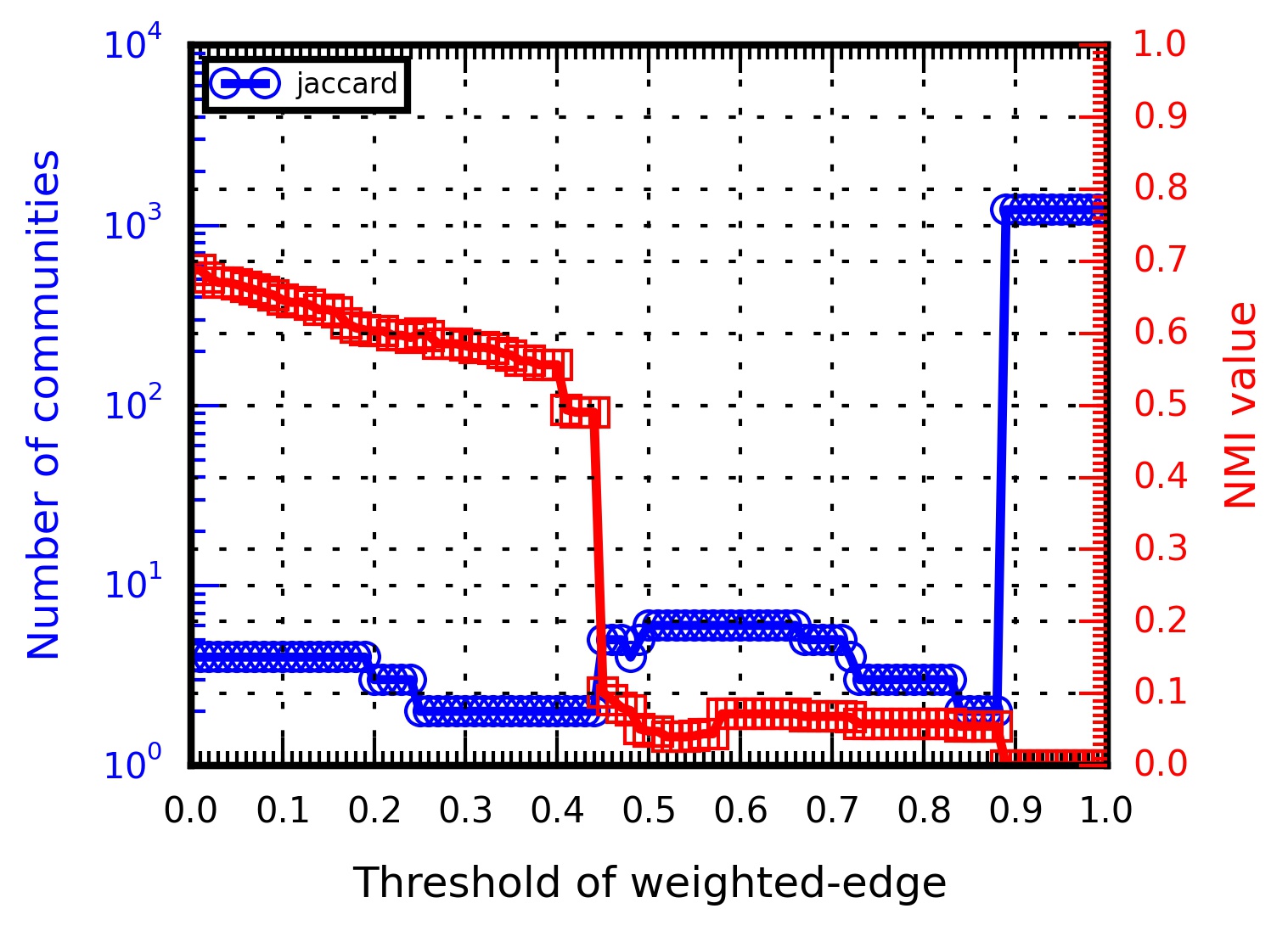 |
| --- | --- | --- |
| (a) Minimum similarity | (b) Cosine similarity | (c) Jaccard index |
| **Fig S8-5. Multi-resolution analysis of different similarities for Polblogs network.** (a) Minimum similarity, (b) Cosine similarity, (c) Jaccard index. | | |

| 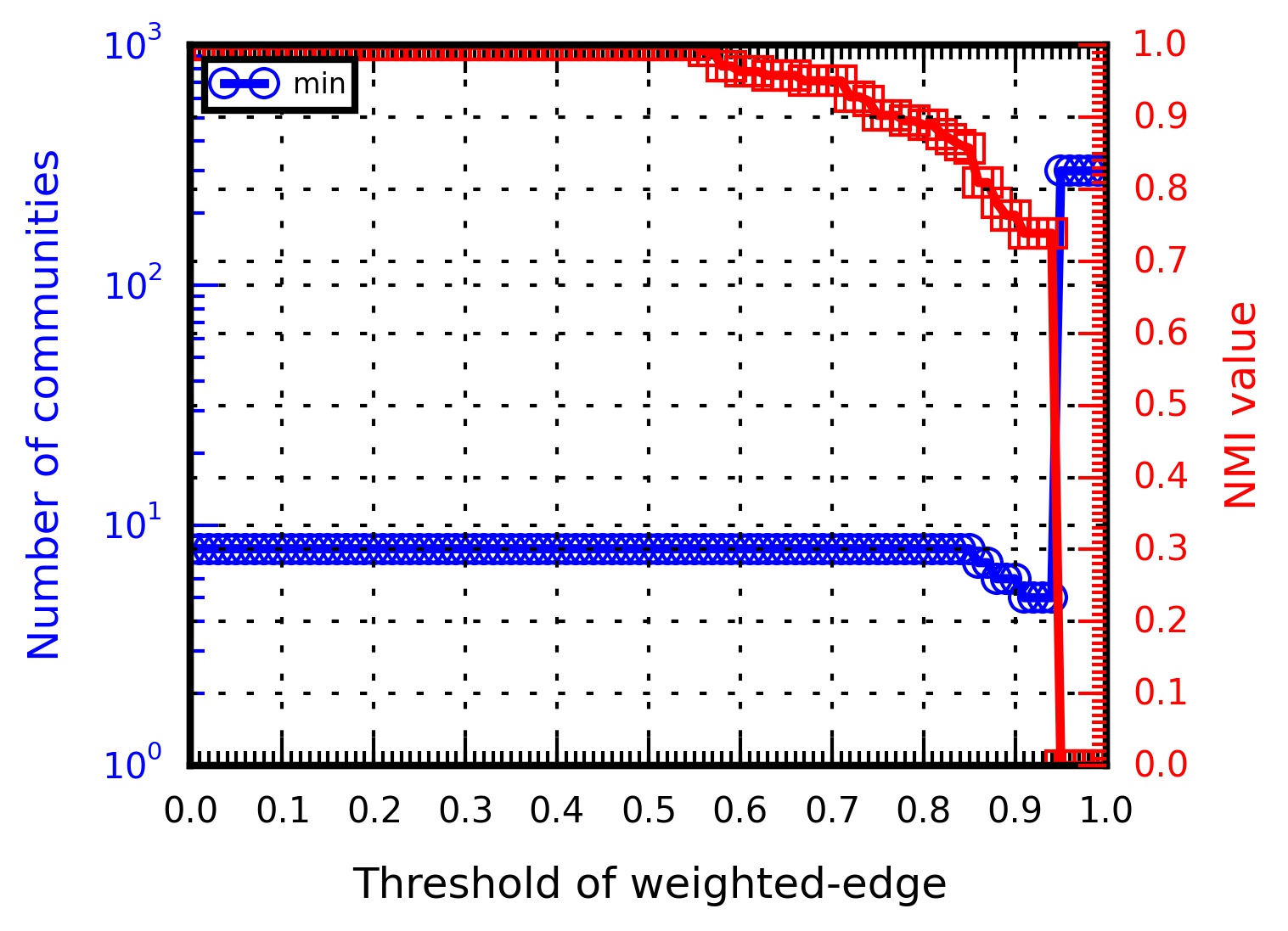 | 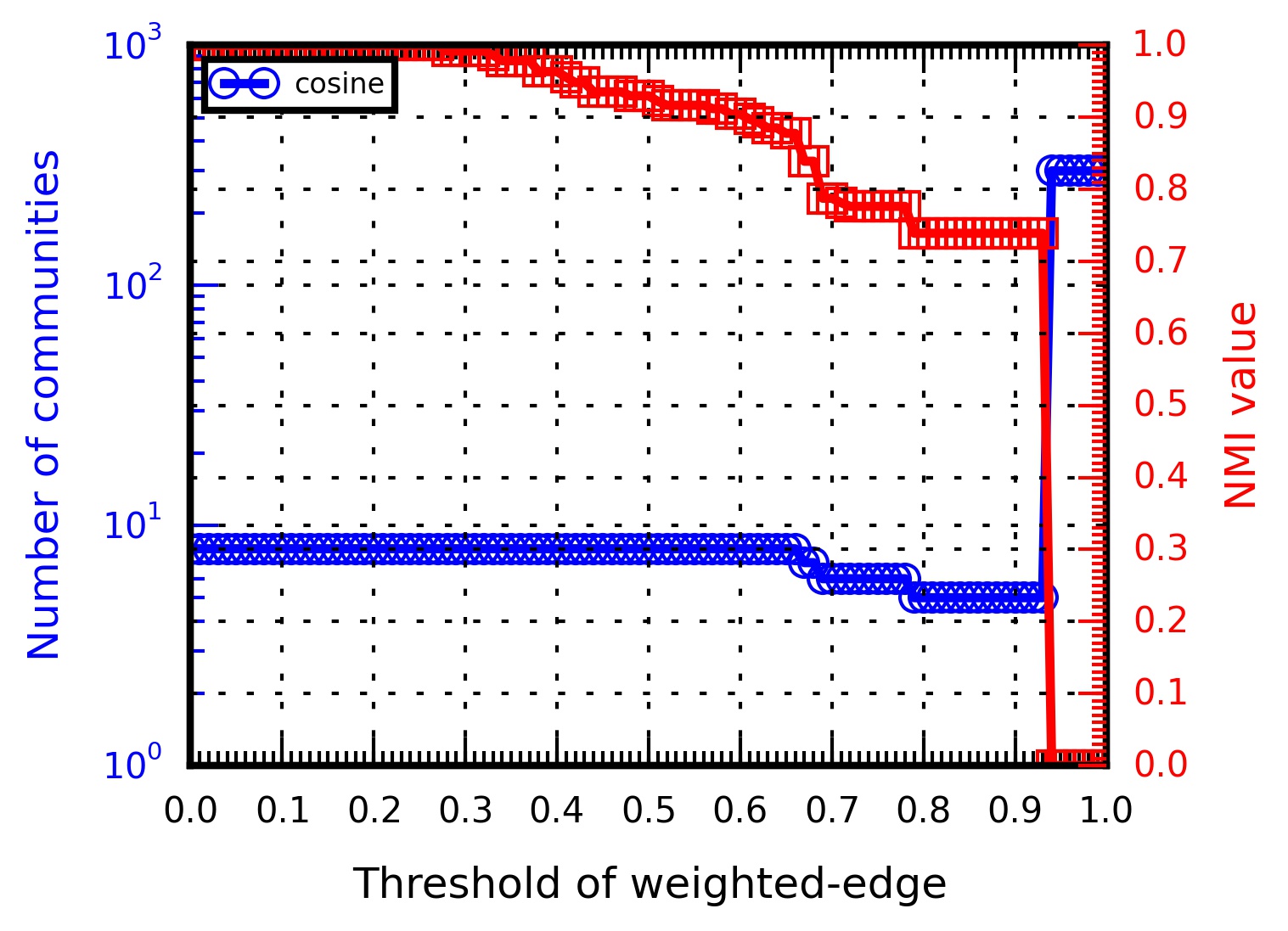 | 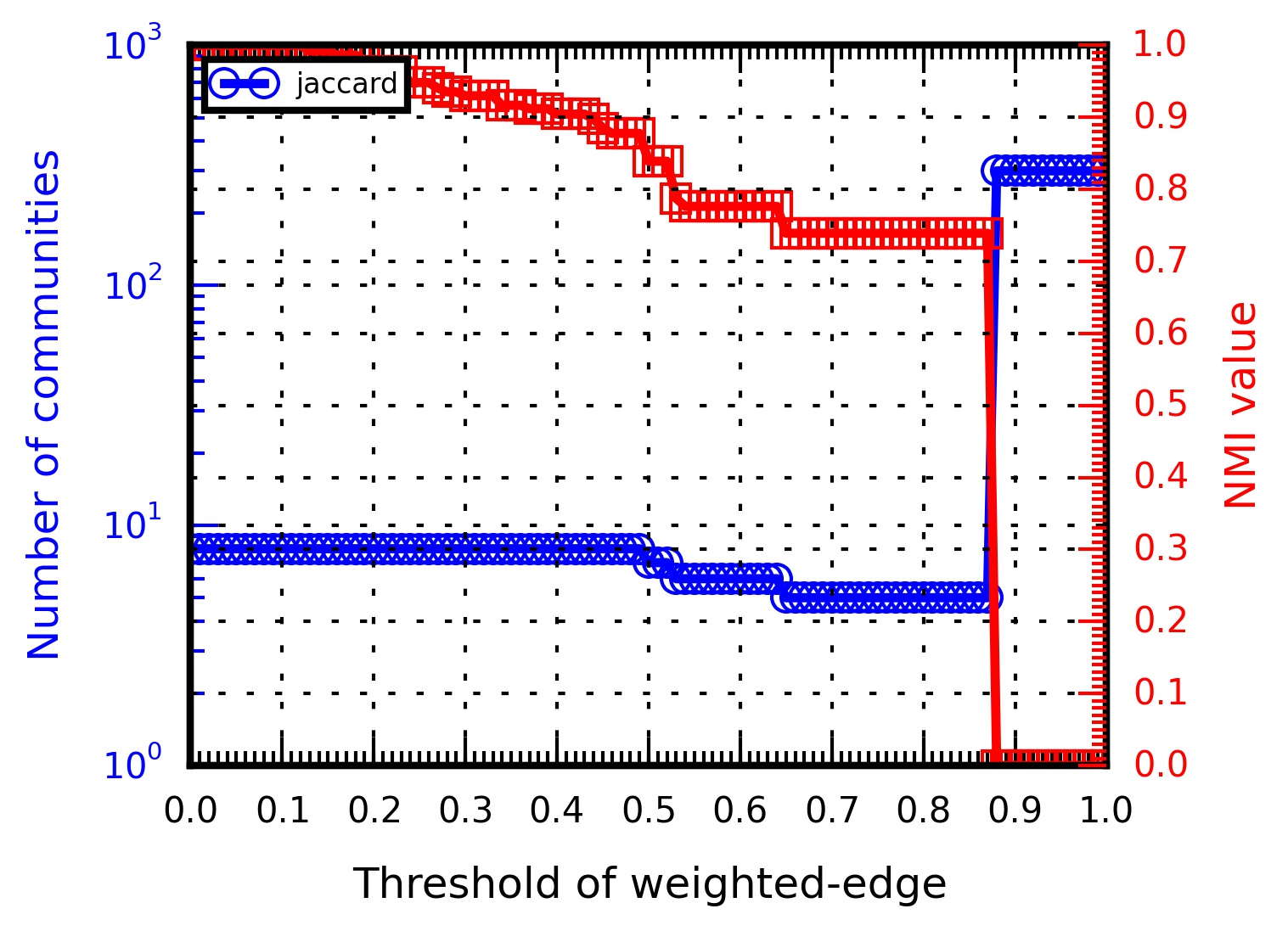 |
| --- | --- | --- |
| (a) Minimum similarity | (b) Cosine similarity | (c) Jaccard index |
| Fig S8-6. Multi-resolution analysis of different similarities for LFR-benchmark-n=300-u=0.05 network. | | |

| 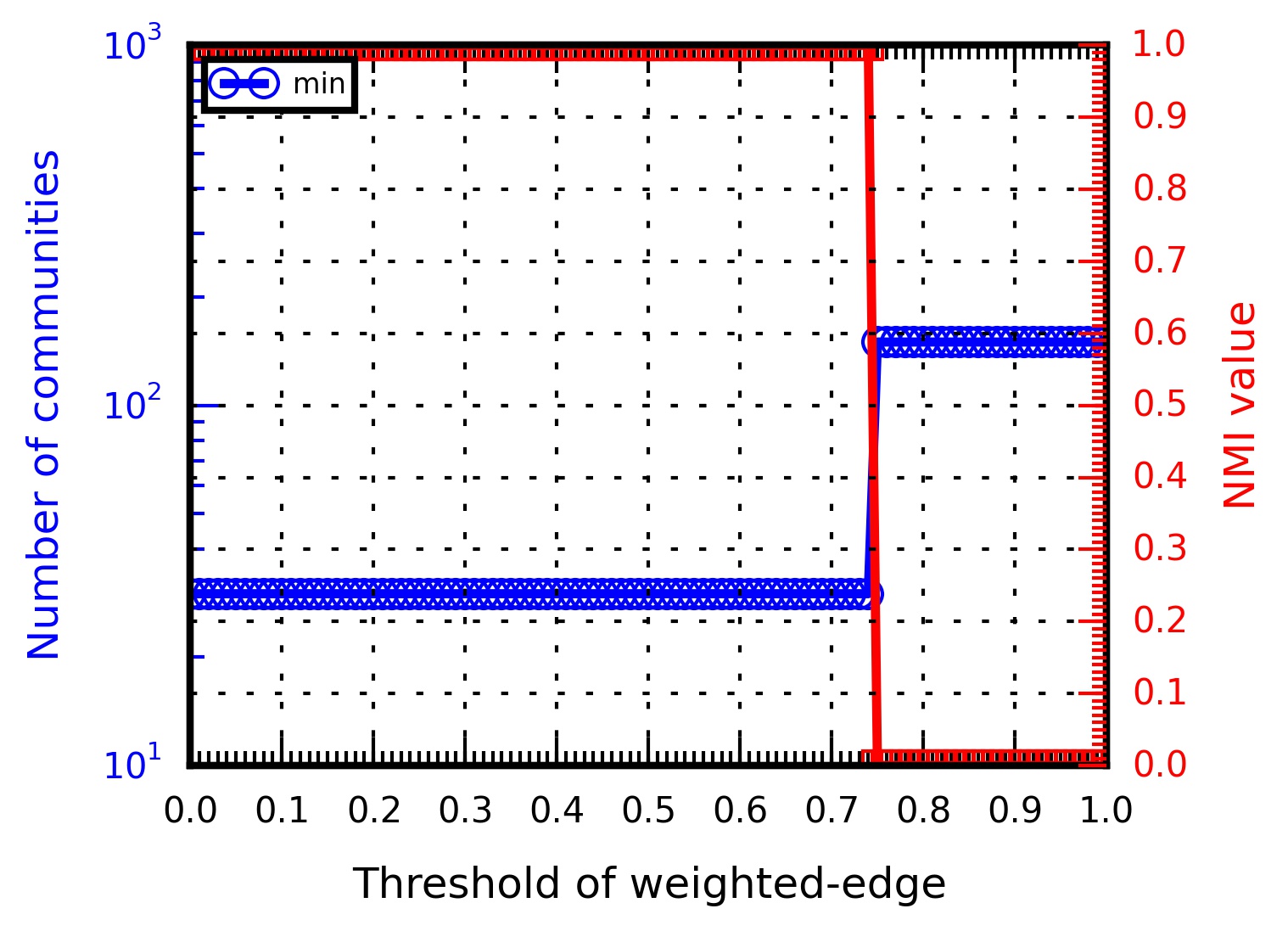 | 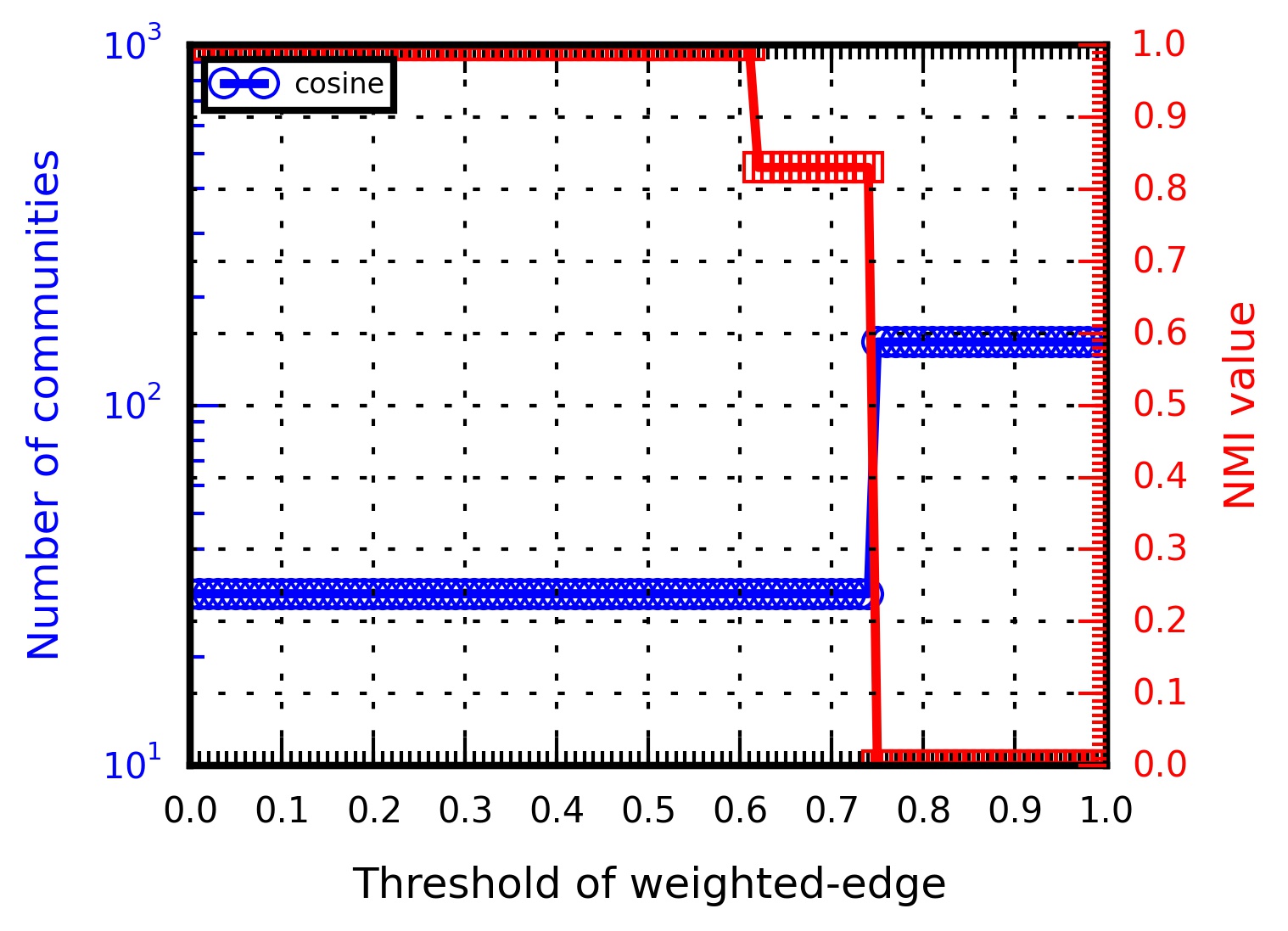 | 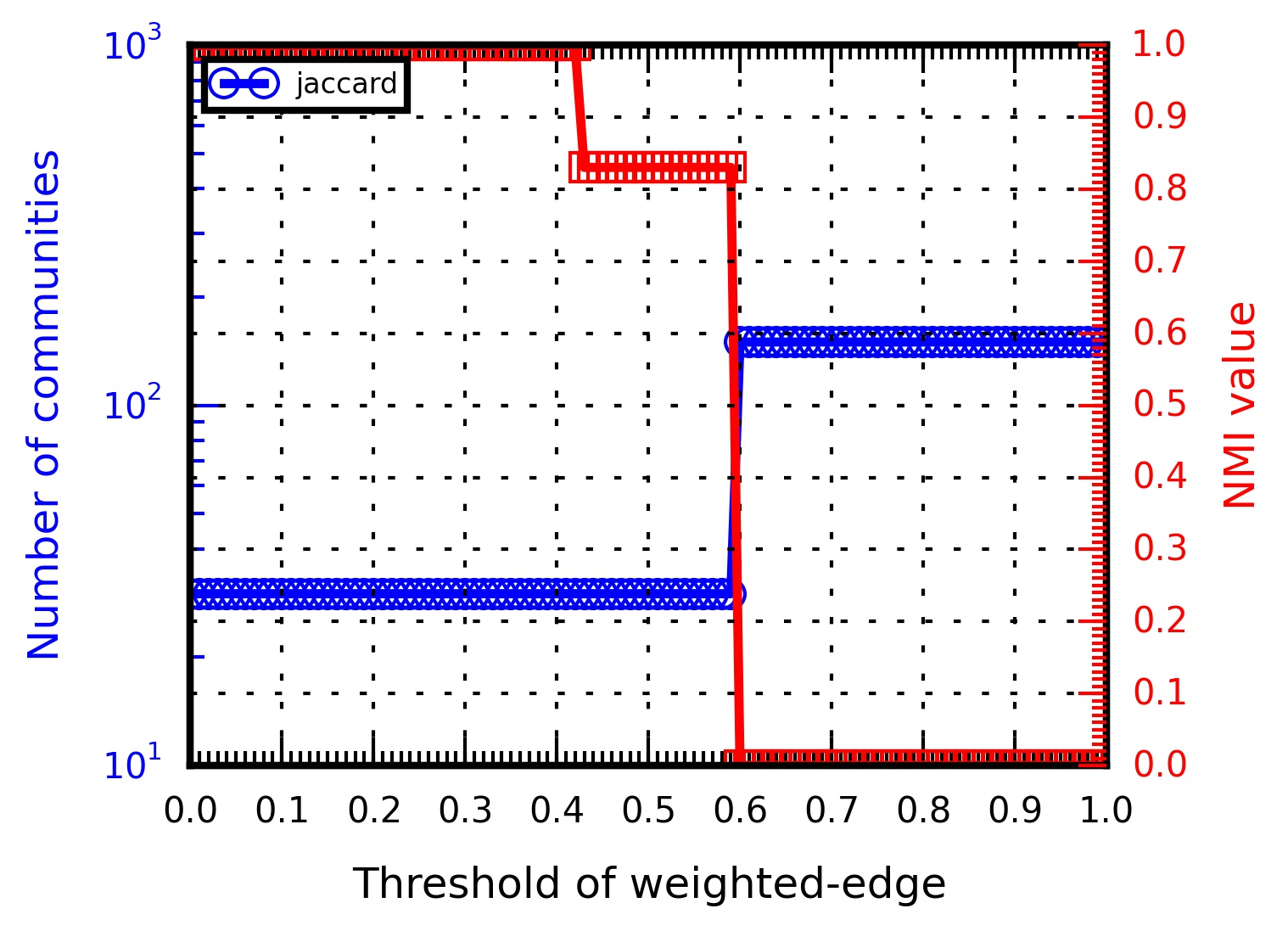 |
| --- | --- | --- |
| (a) Minimum similarity | (b) Cosine similarity | (c) Jaccard index |
| **Fig S8-7. Multi-resolution analysis of different similarities for Clique-ring-m=5-n=30 network.** (a) Minimum similarity, (b) Cosine similarity, (c) Jaccard index. | | |

| 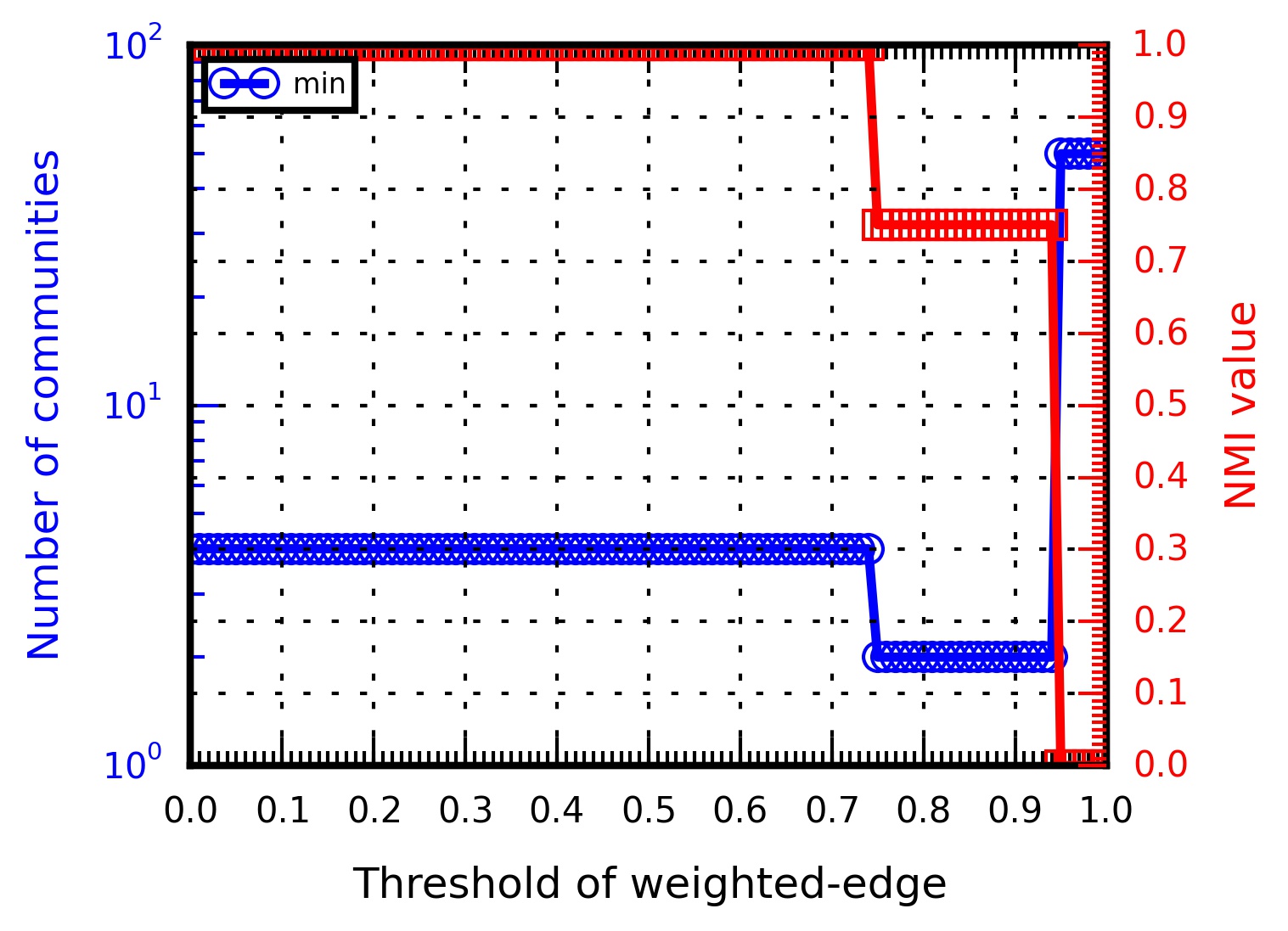 | 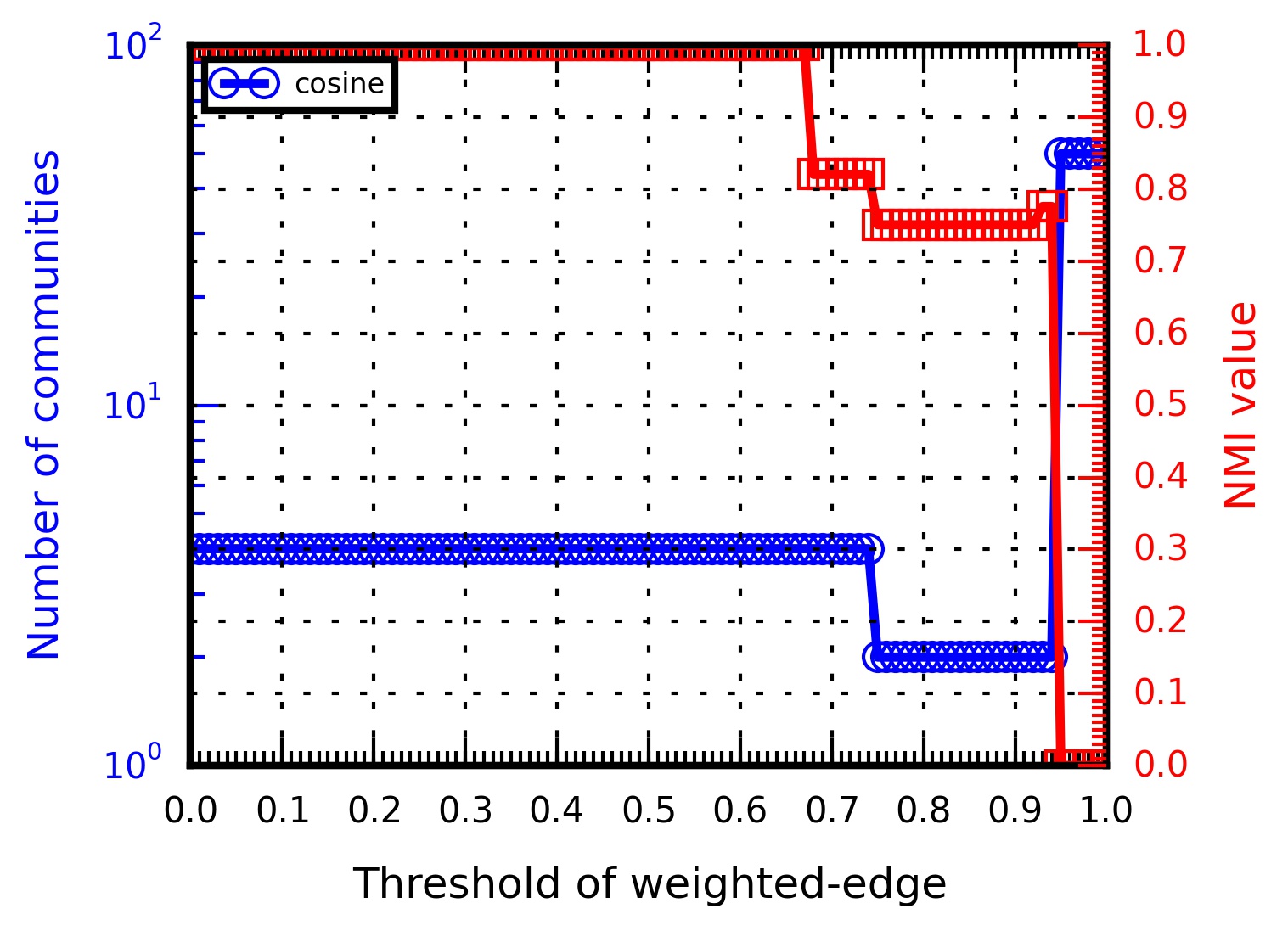 | 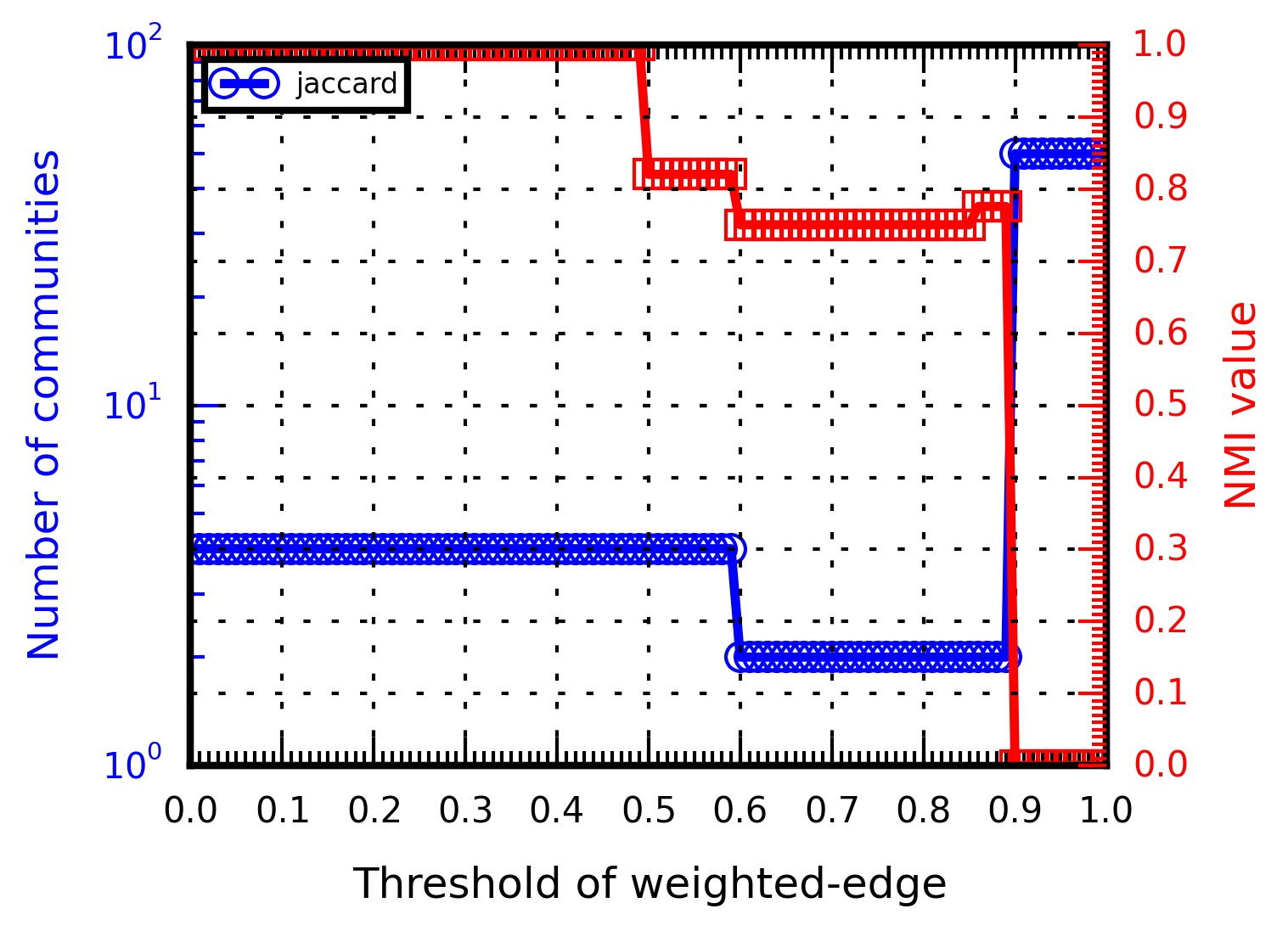 |
| --- | --- | --- |
| (a) Minimum similarity | (b) Cosine similarity | (c) Jaccard index |
| **Fig S8-8. Multi-resolution analysis of different similarities for Clique-pair-m=20_p=5 network.** (a) Minimum similarity, (b) Cosine similarity, (c) Jaccard index. | | |
